# Reconstruction of ancient microbial genomes from the human gut

**DOI:** 10.1038/s41586-021-03532-0

**Published:** 2021-05-12

**Authors:** Marsha C. Wibowo, Zhen Yang, Maxime Borry, Alexander Hübner, Kun D. Huang, Braden T. Tierney, Samuel Zimmerman, Francisco Barajas-Olmos, Cecilia Contreras-Cubas, Humberto García-Ortiz, Angélica Martínez-Hernández, Jacob M. Luber, Philipp Kirstahler, Tre Blohm, Francis E. Smiley, Richard Arnold, Sonia A. Ballal, Sünje Johanna Pamp, Julia Russ, Frank Maixner, Omar Rota-Stabelli, Nicola Segata, Karl Reinhard, Lorena Orozco, Christina Warinner, Meradeth Snow, Steven LeBlanc, Aleksandar D. Kostic

**Affiliations:** 1 Section on Pathophysiology and Molecular Pharmacology, Joslin Diabetes Center, Boston, MA USA; 2 Department of Microbiology, Harvard Medical School, Boston, MA USA; 3 Department of Combinatorics and Optimization, University of Waterloo, Waterloo, Ontario Canada; 4 Department of Archaeogenetics, Max Planck Institute for the Science of Human History, Jena, Germany; 5 CIBIO Department, University of Trento, Trento, Italy; 6 Research and Innovation Centre, Fondazione Edmund Mach, San Michele all’Adige, Italy; 7 Department of Biomedical Informatics, Harvard Medical School, Boston, MA USA; 8 Immunogenomics and Metabolic Diseases Laboratory, Secretaría de Salud, Instituto Nacional de Medicina Genómica, Mexico City, Mexico; 9 Center for Cancer Research, National Cancer Institute, National Institutes of Health, Bethesda, MD USA; 10 Research Group for Genomic Epidemiology, National Food Institute, Technical University of Denmark, Kongens Lyngby, Denmark; 11 Department of Anthropology, University of Montana, Missoula, MT USA; 12 Department of Anthropology, Northern Arizona University, Flagstaff, AZ USA; 13 Pahrump Paiute Tribe and Consolidated Group of Tribes and Organizations, Pahrump, NV USA; 14 Department of Gastroenterology, Hepatology and Nutrition, Boston Children’s Hospital, Boston, MA USA; 15 Morrison Microscopy Core Research Facility, Center for Biotechnology, University of Nebraska-Lincoln, Lincoln, NE USA; 16 Institute for Mummy Studies, EURAC Research, Bolzano, Italy; 17 Center Agriculture Food Environment (C3A), University of Trento, Trento, Italy; 18 School of Natural Resources, University of Nebraska-Lincoln, Lincoln, NE USA; 19 Department of Anthropology, Harvard University, Cambridge, MA USA; 20 Faculty of Biological Sciences, Friedrich-Schiller University, Jena, Germany; 21 Peabody Museum of Archaeology and Ethnology, Harvard University, Cambridge, MA USA

**Keywords:** Archaeology, Metagenomics, Microbiome

## Abstract

Loss of gut microbial diversity^1–6^ in industrial populations is associated with chronic diseases^7^, underscoring the importance of studying our ancestral gut microbiome. However, relatively little is known about the composition of pre-industrial gut microbiomes. Here we performed a large-scale de novo assembly of microbial genomes from palaeofaeces. From eight authenticated human palaeofaeces samples (1,000–2,000 years old) with well-preserved DNA from southwestern USA and Mexico, we reconstructed 498 medium- and high-quality microbial genomes. Among the 181 genomes with the strongest evidence of being ancient and of human gut origin, 39% represent previously undescribed species-level genome bins. Tip dating suggests an approximate diversification timeline for the key human symbiont *Methanobrevibacter smithii*. In comparison to 789 present-day human gut microbiome samples from eight countries, the palaeofaeces samples are more similar to non-industrialized than industrialized human gut microbiomes. Functional profiling of the palaeofaeces samples reveals a markedly lower abundance of antibiotic-resistance and mucin-degrading genes, as well as enrichment of mobile genetic elements relative to industrial gut microbiomes. This study facilitates the discovery and characterization of previously undescribed gut microorganisms from ancient microbiomes and the investigation of the evolutionary history of the human gut microbiota through genome reconstruction from palaeofaeces.

## Abstract (web summary)

Ancient microbiomes from palaeofaeces are more similar to non-industrialized than industrialized human gut microbiomes regardless of geography, but 39% of their de novo reconstructed genomes represent previously undescribed microbial species.

## Main

Previous studies have shown that industrial lifestyles are correlated with both a lower diversity in the gut microbiome^1–6^ and increased incidence of chronic diseases, such as obesity and autoimmune diseases^7^. Examining our ancestral gut microbiome may provide insights into aspects of human–microbiome symbioses that have become altered in the present-day industrialized world^8^.

Reconstruction of metagenome-assembled genomes (MAGs) is an emerging approach to recover high-quality genomes and previously undescribed species-level genome bins (SGBs) from shotgun metagenomics data. Sequencing reads are de novo assembled into contiguous sequences (contigs), and contigs are binned to form draft genomes^9^. The first large-scale initiative to de novo assemble genomes from metagenomic samples in 2017 recovered almost 8,000 MAGs^10^. In 2019, three studies separately reconstructed around 60,000 (ref. ^11^), 90,000 (ref. ^12^) and 150,000 (ref. ^13^) MAGs—including many previously undescribed SGBs (that is, SGBs not assigned to any previously discovered species)—from human microbiome samples.

Despite the potential of de novo assembly to discover previously undescribed SGBs, this method has not been applied to palaeofaeces because of the challenges posed by highly damaged DNA. Therefore, previous studies have focused on describing the taxonomic composition of ancient microbiomes using reference-based approaches^14–16^ or the enrichment of sequences that match specific species and the reconstruction of genomes within that species^6,17–19^. These approaches enable the recovery of microorganisms that belong to, or are closely related to, species that are present in the reference database, but not the discovery of new species. In this study, we performed a large-scale de novo assembly of microbial genomes from palaeofaeces.

## Ethics

Although palaeofaeces are not subject to the Native American Graves Protection and Repatriation Act (NAGPRA) or other regulations, we engaged in consultation with living communities who maintain strong cultural ties to the palaeofaeces. This included involvement of the Robert S. Peabody Institute of Archaeology, which distributed correspondence to Southwest Tribal Historic Preservation Officers (THPOs) and tribal government offices to promote transparency and provide an opportunity to discuss the study. Consultation consisted of interactive short presentations to provide an overview of the research with time to respond to questions, as well as follow-up materials and opportunities for expanded dialogue to ensure topics of interest and concerns were addressed. We anticipate this process will continue, despite the constraints of the COVID-19 pandemic. Additional information is provided in the Supplementary Information.

## Overview of samples

**Fig. 1:**
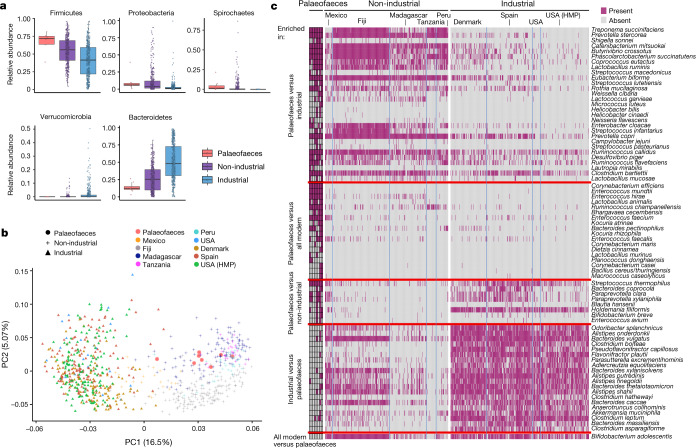
Phylum, family and species compositions of the palaeofaeces samples are similar to the gut microbiomes of present-day non-industrial individuals. **a**, Differentially abundant phyla (one-tailed Wilcoxon rank-sum test with FDR correction) as identified by MetaPhlAn2^20^ (palaeofaeces, *n* = 8; non-industrial, *n* = 370; industrial, *n* = 418). Data are presented as box plots (middle line, median; lower hinge, first quartile; upper hinge, third quartile; lower whisker, the smallest value at most 1.5× the interquartile range from the hinge; upper whisker, the largest value no further than 1.5× the interquartile range from the hinge; data beyond the whiskers are outlying points). **b**, Principal component analysis of the species composition as identified by MetaPhlAn2^20^. HMP, Human Microbiome Project. **c**, Presence–absence heat map (fuchsia, present; grey, absent) for differentially enriched species (two-tailed Fisher’s test, FDR correction). Species without fully specified species names are not shown (a complete list is included in Supplementary Table 3).

**Extended Data Fig. 1:**
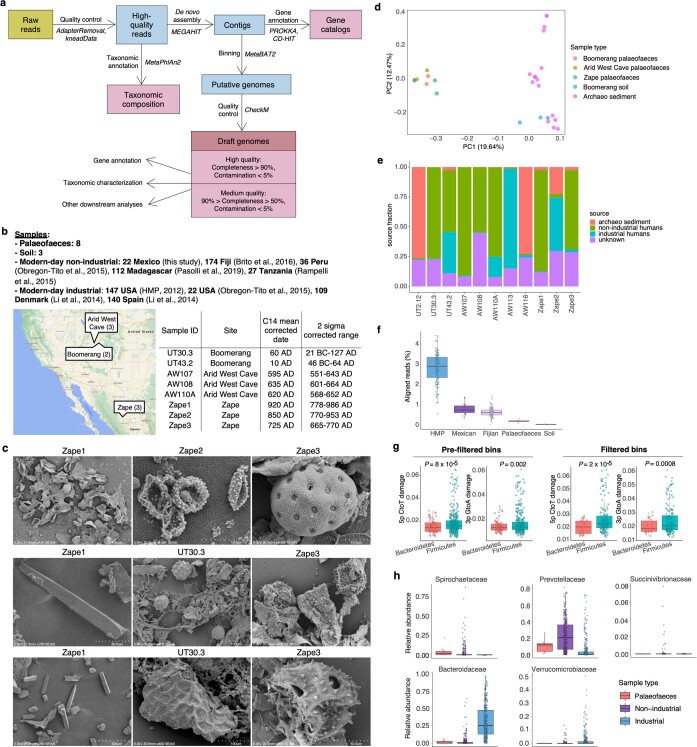
Overview of samples, study design and quality measures to validate the authenticity of the palaeofaeces. **a**, Schematic of gene-catalogue and genome-reconstruction pipelines. **b**, Samples used in this study, archaeological sites and ^14^C dating. Data were obtained from this study (Mexico) and previous studies: Fiji^31^, Peru^4^, Madagascar^13^, Tanzania^57^, USA^4,55^, Denmark^56^ and Spain^56^. Map data are from Google Maps (2021 Google, INEGI). **c**, Scanning electron microscopy images of dietary remains in the palaeofaeces. Zape1, maize pollen grains (more than 191,000 grains per gram) (top) and agave phytoliths (middle and bottom). Zape2, *U. maydis* spores (hundreds of millions per gram). Zape3, Chenopod or amaranth foliage and/or buds (smaller pollen) and squash (larger pollen with spines). UT30.3, druse phytoliths, annular xylem vessel secondary wall thickenings and epidermis of Cactaceae. A complete description is provided in Supplementary Information section 2. Reproducibility and independently repeated experiments are described in the Methods. **d**, Principal component analysis of the species composition of palaeofaeces, soil samples and publicly available archaeological sediment samples^70,71^. Species were identified by MetaPhlAn2^20^. **e**, Prediction of source of microbial communities by SourceTracker2^72^ using the species abundance matrix from MetaPhlAn2 as input. Archaeological sediment samples included three soil samples collected in this study, seven Holocene human-associated sediments from CoproID^71^ and 40 Pleistocene sediment samples^70^. **f**, The percentage of reads aligned to the MetaPhlAn2 database per sample (HMP, *n* = 146; Mexican, *n* = 22; Fijian, *n* = 174; palaeofaeces, *n* = 8; soil, *n* = 3) (Supplementary Information section 4). **g**, aDNA damage levels of Firmicutes and Bacteroidetes genomes for medium-quality and high-quality pre-filtered and filtered bins (two-tailed Wilcoxon rank-sum test; pre-filtered bins Bacteroidetes, *n* = 69 MAGs; pre-filtered bins Firmicutes, *n* = 359 MAGs; filtered bins Bacteroidetes, *n* = 24 MAGs; filtered bins Firmicutes, *n* = 161 MAGs) (Supplementary Information section 5). 5p, 5′ end; 3p, 3′ end. **h**, Abundances of VANISH^21^ and BloSSUM^22^ families as identified by MetaPhlAn2^20^ (palaeofaeces *n* = 8; non-industrial *n* = 370; industrial *n* = 418). In **f**–**h**, data are presented as box plots (middle line, median; lower hinge, first quartile; upper hinge, third quartile; upper whisker extends from the hinge to the largest value no further than 1.5× the interquartile range from the hinge; lower whisker extends from the hinge to the smallest value at most 1.5× the interquartile range from the hinge; data beyond the end of the whiskers are individually plotted outlying points).

**Extended Data Fig. 2:**
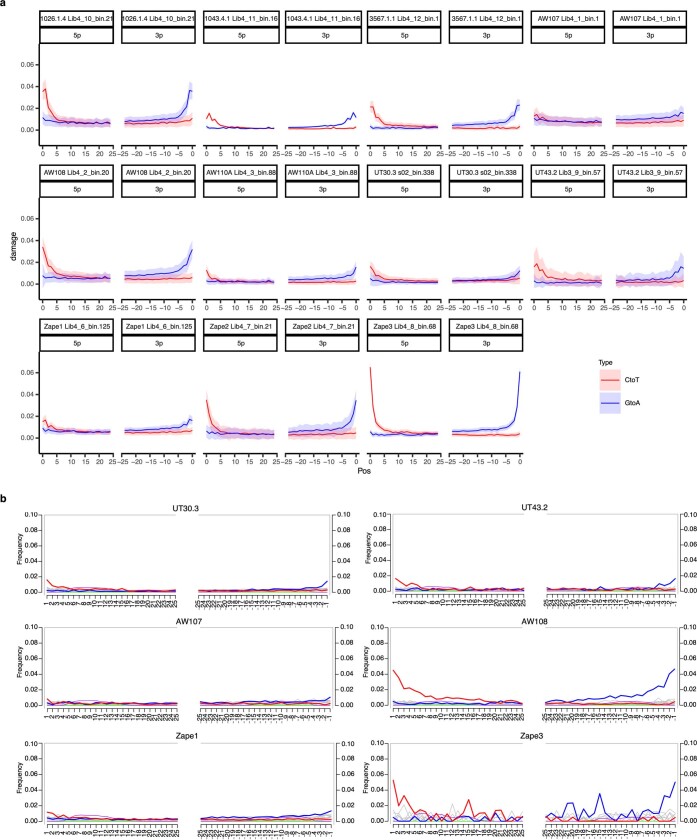
Microbial DNA and mtDNA damage patterns. **a**, Microbial damage patterns of the palaeofaeces and the Boomerang soil samples as identified by DamageProfiler^88^. A medium-quality or high-quality reconstructed genome was used as reference for its respective sample. All MAGs used as reference genomes for the palaeofaeces are of known gut microbial species. The red line indicates the average frequency of C-to-T substitutions across all contigs per bin and the blue line indicates the average frequency of G-to-A substitutions across all contigs per bin. The shaded areas show the s.d. (1026.1.4 Lib4_10_bin.21, *n* = 488 contigs; 1043.4.1 Lib4_11_bin.16, *n* = 133 contigs; 3567.1.1 Lib4_12_bin.1, *n* = 278 contigs; AW107 Lib4_1_bin.1, *n* = 208 contigs; AW108 Lib4_2_bin.20, *n* = 337 contigs; AW110A Lib4_3_bin.88, *n* = 210 contigs; UT30.3 s02_bin.338, *n* = 74 contigs; UT43.2 Lib3_9_bin.57, *n* = 174 contigs; Zape1 Lib4_6_bin.125, *n* = 212 contigs; Zape2 Lib4_7_bin.21, *n* = 241 contigs; Zape3 Lib4_8_bin.68, *n* = 324 contigs). Contigs with fewer than 1,000 reads aligned were removed from the analysis. **b**, mtDNA damage patterns of the palaeofaeces as identified by mapDamage2.0^87^. Human mtDNA (rCRS) was used as reference. The red line indicates the average frequency of C-to-T substitutions and the blue line indicates the average frequency of G-to-A substitutions. Samples AW110A and Zape2 did not have enough mtDNA reads for mtDNA damage assessment.

**Extended Data Fig. 3:**
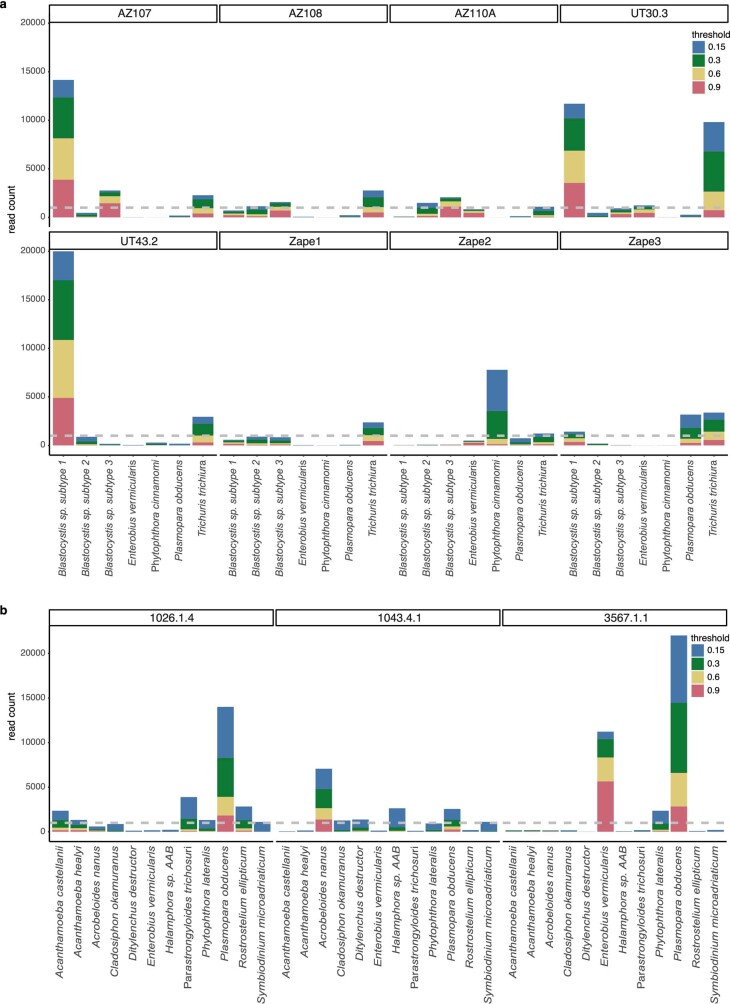
Parasites in the palaeofaeces and the soil samples classified using Kraken 2. The bars represent the reads assigned with a Kraken^74^ confidence threshold between 0.15 and 0.9. The value specifies the fraction of *k*-mers needed for the specific classification level. The grey dotted line indicates the 1,000 reads cut-off. The displayed parasites were detected above the cut-off in at least one sample. **a**, Parasites in the palaeofaeces. In six out of eight palaeofaeces samples, *Blastocystis* is above the cut-off. Subtype 1 is the dominant subtype in samples AW107, UT30.3, UT43.2 and Zape3, whereas subtype 3 is the dominant subtype in AW108 and AW110A. Other parasites do not meet the cut-off requirements described in the Methods. **b**, Parasites in the soil samples include *Acanthamoeba* (a parasite frequently found in soil) in sample 1026.1.4 and *Enterobius vermicularis* (human pinworm) in sample 3567.1.1.

**Extended Data Fig. 4:**
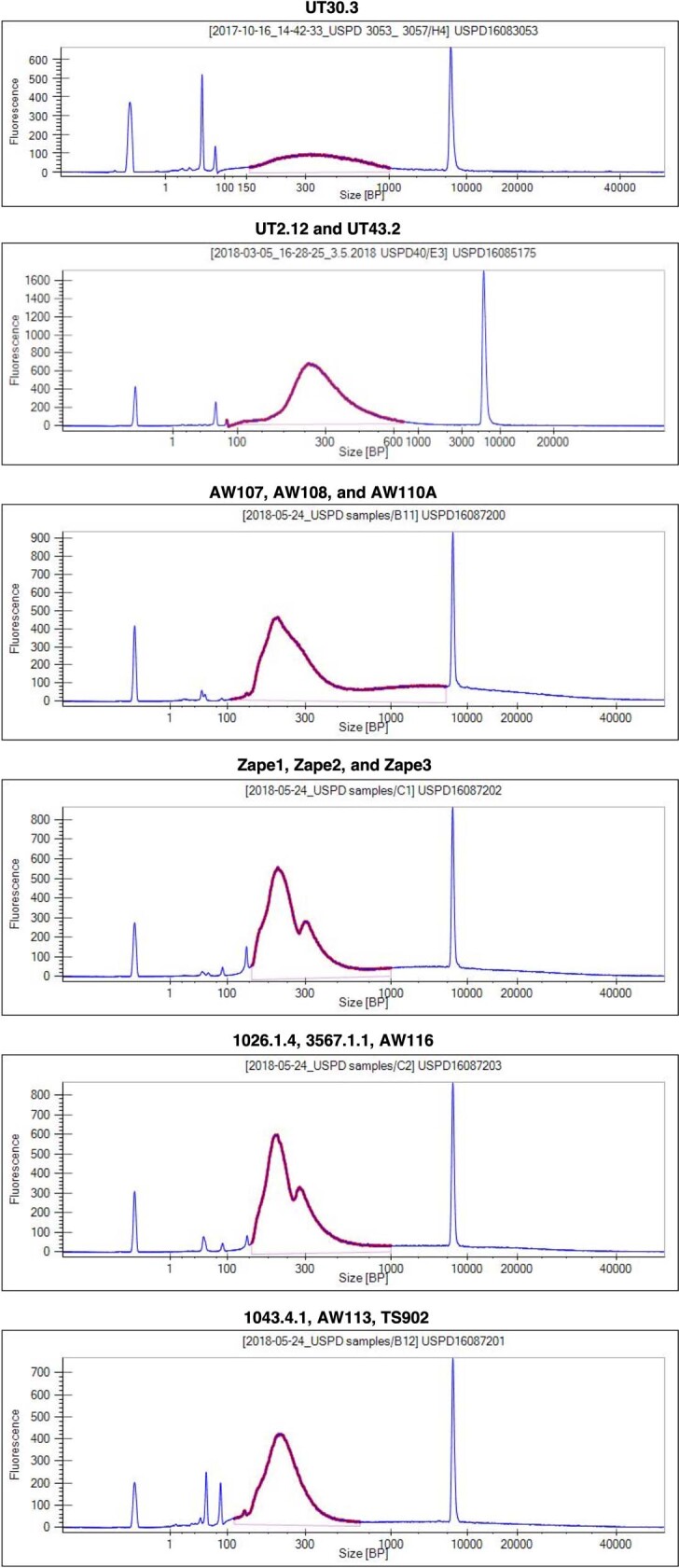
BioAnalyzer results showing the length distribution of DNA fragments per library. The libraries contain 120-bp adapters.

**Extended Data Fig. 5:**
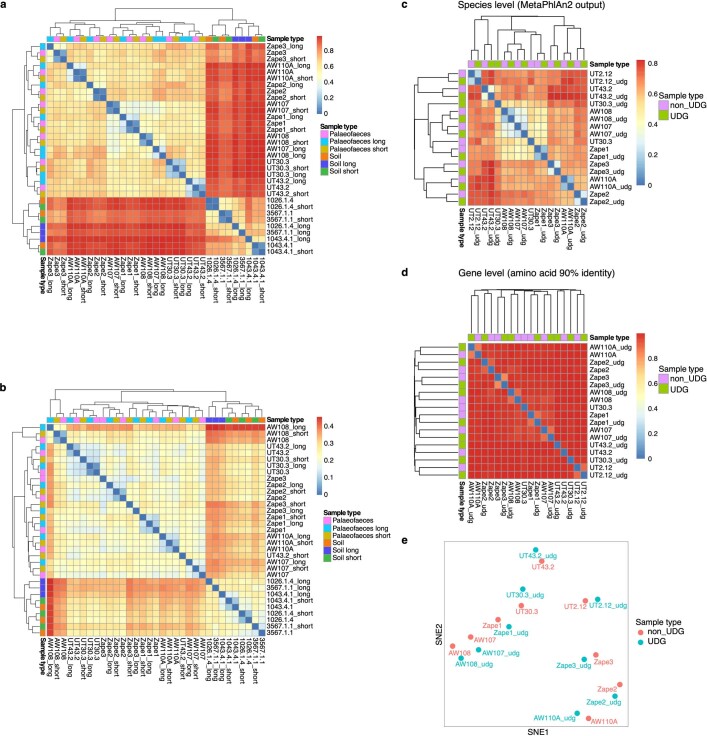
Species and gene content of long versus short DNA fragments and UDG-treated versus non-UDG-treated samples. **a**, **b**, Pairwise comparison between whole samples, only subsets containing short reads and only subsets with long reads. **a**, Heat map of species-level pairwise Jaccard distances for whole samples, short-read subsets (reads ≤ 145 bp) and long-read subsets (reads > 145 bp). Species were identified by MetaPhlAn2^20^. The groups cluster together by sample. **b**, Heat map of gene-level pairwise Jaccard distances for whole samples, short-read subsets and long-read subsets. Genes were identified by PROKKA^38^ and a count matrix was built from PROKKA output files. Groups from the same sample cluster together. **c**–**e**, Species and gene content comparison between UDG-treated libraries and non-UDG-treated libraries (Supplementary Information section 12 and Supplementary Table 10). **c**, Heat map of species-level pairwise Jaccard distances between each pair of all UDG-treated and non-UDG-treated samples. Species were identified by MetaPhlAn2^20^. Each UDG-treated library clusters with non-UDG-treated library from the same sample. **d**, Heat map of gene-level pairwise Jaccard distances between each pair of all UDG-treated and non-UDG-treated samples. Genes were identified by PROKKA^38^ and non-redundant gene catalogues were generated by collapsing genes within 10% amino acid identity distance. Each UDG-treated library clusters with non-UDG-treated library from the same sample. **e**, *t*-Distributed stochastic neighbour embedding (*t*-SNE) analysis at the species level shows clustering of each UDG-treated library with the non-UDG-treated library from the same sample.

We performed shotgun metagenomic sequencing on 15 palaeofaeces samples (Supplementary Table 1). The samples and authentication methods are described in Supplementary Information section 1. In brief, we excluded seven palaeofaeces samples because of poor de novo assembly results (Supplementary Table 1), evidence of archaeological soil contamination (Extended Data Fig. 1e) or a nonhuman host source (Supplementary Table 1). The remaining eight samples came from three sites (Boomerang Shelter, Arid West Cave and Zape) (Extended Data Fig. 1b). Their authenticity was extensively validated (Supplementary Information section 1), including their ancient origin (Extended Data Fig. 2) and human source (Extended Data Fig. 1c, Supplementary Tables 1, 2 and Supplementary Information section 2). Our results support that the palaeofaeces are faecal samples with minimal soil contamination (Fig. 1b, Extended Data Figs. 1d, e, 3 and Supplementary Tables 3, 4). The final eight samples are well-preserved and have long average DNA fragment sizes (average mode length = 174 base pairs (bp), s.d. = 30.15) (Extended Data Fig. 4). We confirmed that these long DNA fragments are not from contamination by modern DNA (Extended Data Fig. 5 and Supplementary Table 5).

As a comparison to the ancient gut microbiome, we analysed 789 present-day stool samples from both industrial and non-industrial populations across eight countries (Extended Data Fig. 1b and Supplementary Table 1). These include publicly available gut metagenomes and samples that we collected from 22 individuals living in a rural Mazahua farming community in central Mexico.

## Reference-based taxonomic composition

We analysed the taxonomic composition with MetaPhlAn2^20^ (Supplementary Table 3), which is a reference-based tool. Consistent with previous observations^15^, the taxonomic composition of the palaeofaeces is more similar to that of the non-industrial samples than the industrial samples (Fig. 1). None of the phyla is significantly different between the palaeofaeces and the non-industrial samples. By contrast, Bacteroidetes and Verrucomicrobia are enriched in the industrial samples compared to the palaeofaeces (one-tailed Wilcoxon rank-sum test with false-discovery rate (FDR) correction, *P* = 0.0003 and *P* = 0.009, respectively) and the non-industrial samples (*P* = 4.6 × 10^−37^ and *P* = 1.1 × 10^−31^, respectively) (Fig. 1a and Supplementary Table 3). Firmicutes, Proteobacteria and Spirochaetes are significantly less abundant in the industrial samples relative to the palaeofaeces (*P* = 0.003, *P* = 0.002 and *P* = 2.8 × 10^−45^, respectively) and the non-industrial samples (*P* = 2.5 × 10^−16^, *P* = 1.7 × 10^−30^ and *P* = 3.6 × 10^−93^, respectively).

At the family level, members of the VANISH (volatile and/or associated negatively with industrialized societies of humans) taxa^21^ are significantly enriched in the palaeofaeces samples relative to the industrial samples (Spirochaetaceae, *P* = 1.8 × 10^−92^; Prevotellaceae, *P* = 0.003) (Extended Data Fig. 1h and Supplementary Table 3). By contrast, members of the BloSSUM (bloom or selected in societies of urbanization/modernization) taxa^22^ are more abundant in the industrial samples compared to both the non-industrial samples and the palaeofaeces samples (Bacteroidaceae, *P* = 1.6 × 10^−106^ and *P* = 0.0004, respectively; Verrucomicrobiaceae, *P* = 2.0 × 10^−31^ and *P* = 0.02, respectively). In comparison to the non-industrial samples, only Spirochaetaceae is enriched in the palaeofaeces (*P* = 0.004).

The species composition of the palaeofaeces also reflects the present-day non-industrial gut microbiome (a complete description is provided in Supplementary Information section 3). Species-level principal component analysis shows that the palaeofaeces samples cluster with the non-industrial samples, and are distinct from the industrial samples (Fig. 1b). Species enriched in the industrial samples relative to both the palaeofaeces and the non-industrial samples include *Akkermansia muciniphila* (two-tailed Fisher’s test with FDR correction, *P* = 2.2 × 10^−2^ and *P* = 9.8 × 10^−30^, respectively) and members of the *Alistipes* and *Bacteroides* genera (Fig. 1c and Supplementary Table 3). On the other hand, *Ruminococcus champanellensis* (*P* = 0.0003 and *P* = 9.6 × 10^−9^, respectively) and members of the *Enterococcus* genus are enriched in the palaeofaeces compared to both the non-industrial and industrial samples. The spirochaete *Treponema succinifaciens* is enriched in both the palaeofaeces and the non-industrial samples relative to the industrial samples (*P* = 2.4 × 10^−14^ and *P* = 1.1 × 10^−117^, respectively). *Treponema succinifaciens* and, more generally, the phylum Spirochaetes (Fig. 1a) have been proposed to be lost in industrial populations^4^. These results support that the industrial human gut microbiome has diverged from its ancestral state^7,8^.

## De novo genome reconstruction

**Fig. 2:**
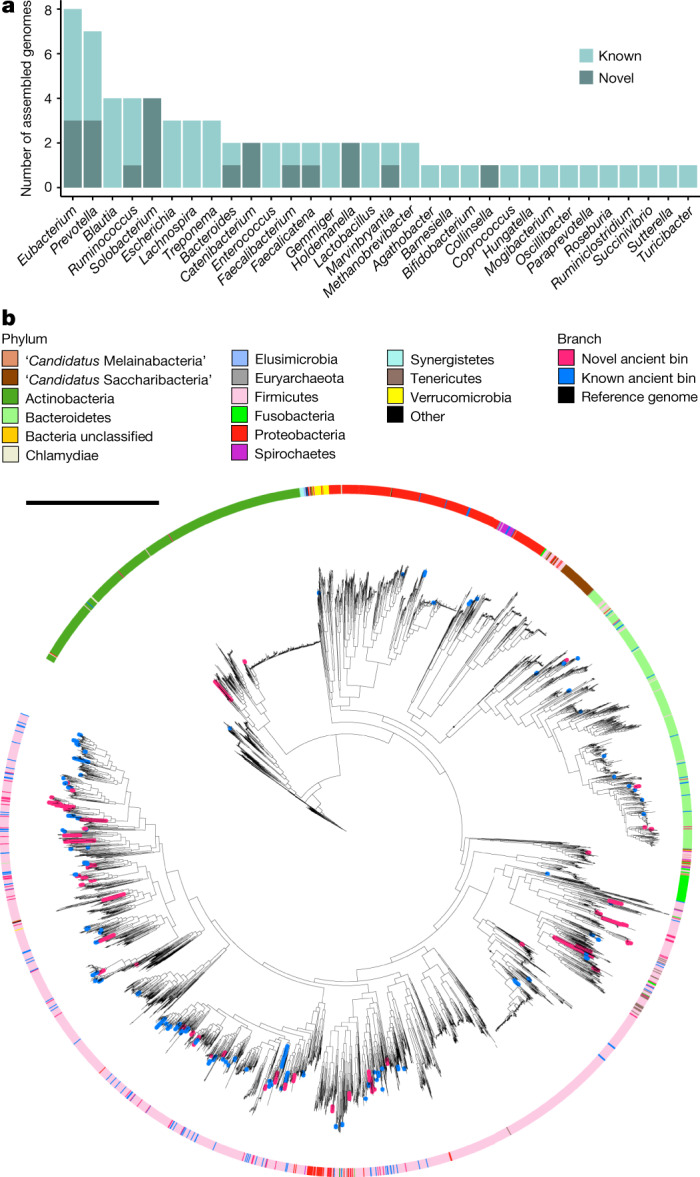
De novo genome reconstruction from palaeofaeces recovers 181 authenticated ancient gut microbial genomes, 39% of which are novel SGBs. **a**, GTDB-Tk^23^ genus estimation for both novel and known species. **b**, Maximum likelihood tree of 178 highly damaged filtered ancient gut bacteria and 4,930 representative human gut microbiome genomes^13^. The tree was constructed using multiple sequence alignment of 120 bacterial marker genes identified by GTDB-Tk^23^. Novel and known ancient bin branches are highlighted in pink and blue, respectively. Tree scale, 1 nucleotide substitution per site.

**Extended Data Fig. 6:**
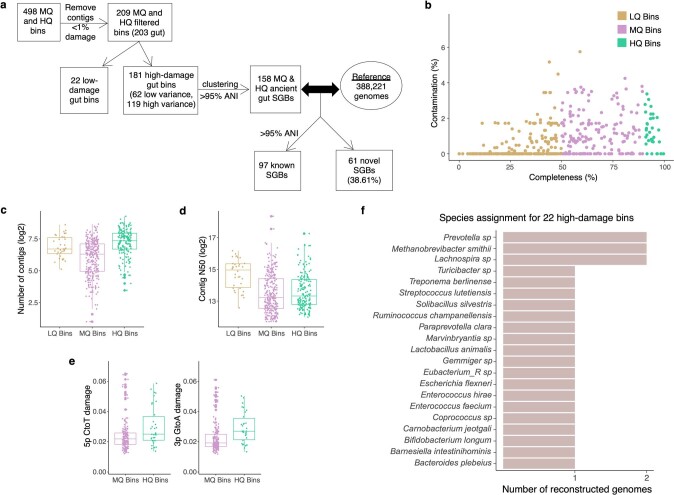
De novo genome reconstruction from palaeofaeces recovers 181 authenticated ancient gut microbial genomes, 39% of which are novel SGBs. Related to Fig. 2. **a**–**d**, CheckM^79^ quality estimation for de novo reconstructed microbial genomes for the 209 filtered bins (low-quality bins, *n* = 285; medium-quality bins, *n* = 175; high-quality bins, *n* = 34). Genomes were classified as low quality (LQ; completeness ≤ 50% or contamination > 5%), medium quality (MQ; 90% ≥ completeness > 50%, contamination < 5%) or high quality (HQ; completeness > 90% and contamination < 5%). **a**, Filtering steps, number of bins that belong to each of the quality categories and classification of novel SGBs. **b**, Contamination and completeness distribution for the filtered bins. **c**, Distribution of the number of contigs for each of the quality categories. **d**, Distribution of contig N50 values for each of the quality categories. **e**, Damage levels, specifically C-to-T substitutions at the 5′ end and G-to-A substitutions at the 3′ end of the reads, for each ancient bin as estimated by DamageProfiler^88^ (medium-quality bins, *n* = 175; high-quality bins, *n* = 34). **f**, GTDB-Tk^23^ species assignment for the known species. In **c**–**e**, data are presented as box plots (middle line, median; lower hinge, first quartile; upper hinge, third quartile; upper whisker extends from the hinge to the largest value no further than 1.5× the interquartile range from the hinge; lower whisker extends from the hinge to the smallest value at most 1.5× the interquartile range from the hinge; data beyond the end of the whiskers are individually plotted outlying points).

**Extended Data Fig. 7:**
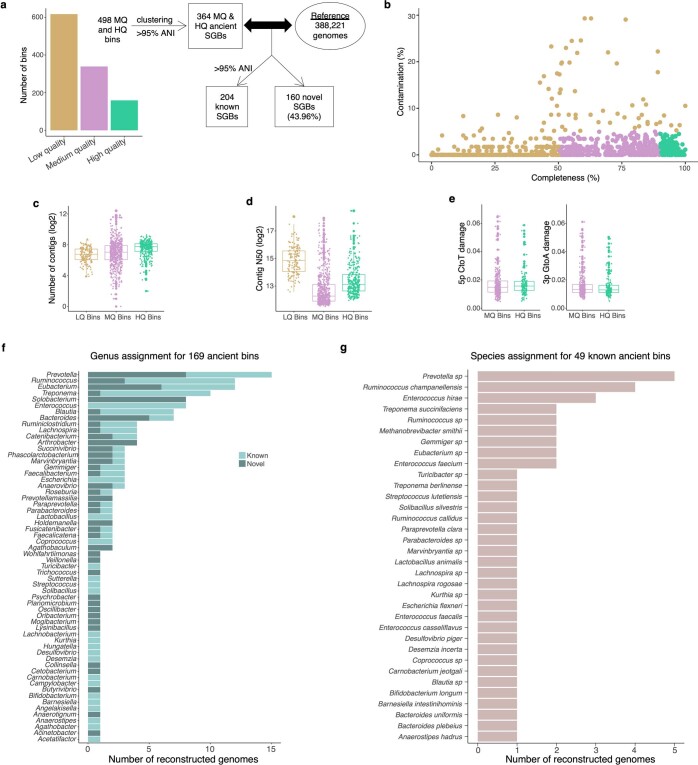
De novo genome reconstruction from palaeofaeces recovers 498 medium- and high-quality microbial genomes, 44% of which are novel SGBs. Related to Fig. 2. **a**–**d**, CheckM^79^ quality estimation of all 498 de novo reconstructed microbial genomes (low-quality bins, *n* = 617; medium-quality bins, *n* = 339; high-quality bins, *n* = 159). Genomes were classified as low quality (completeness ≤ 50% or contamination > 5%), medium quality (90% ≥ completeness > 50% and contamination < 5%) or high quality (completeness > 90% and contamination < 5%). **a**, Number of bins that belong to each of the quality categories and classification of novel SGBs. **b**, Contamination and completeness distribution for the reconstructed genomes. **c**, Distribution of the number of contigs for each of the quality categories. **d**, Distribution of contig N50 values for each of the quality categories. **e**, Damage levels, specifically C-to-T substitutions at the 5′ end and G-to-A substitutions at the 3′ end of the reads, for each bin as estimated by DamageProfiler^88^ (medium-quality bins, *n* = 339; high-quality bins, *n* = 159). **f**, GTDB-Tk^23^ genus estimation for members of both the novel and known SGBs. **g**, GTDB-Tk^23^ species assignment for members of the known SGBs. In **c**–**e**, data are presented as box plots (middle line, median; lower hinge, first quartile; upper hinge, third quartile; upper whisker extends from the hinge to the largest value no further than 1.5× the interquartile range from the hinge; lower whisker extends from the hinge to the smallest value at most 1.5× the interquartile range from the hinge; data beyond the end of the whiskers are individually plotted outlying points).

**Extended Data Fig. 8:**
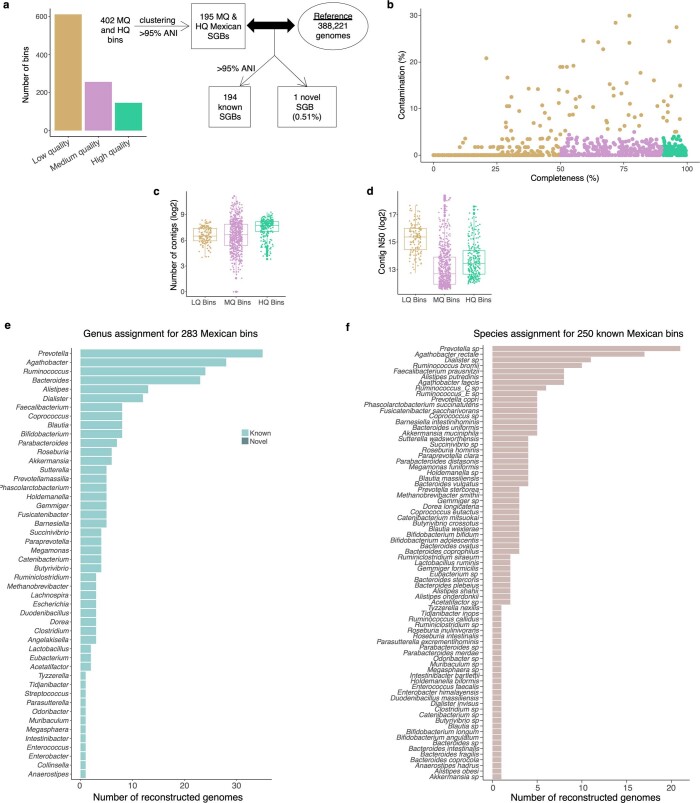
De novo genome reconstruction from present-day individuals of Mexican ancestry recovers 402 medium- and high-quality genomes, only 1 of which is a novel SGB. Related to Fig. 2. **a**–**d**, CheckM^79^ quality estimation of all de novo reconstructed microbial genomes (low-quality bins, *n* = 611; medium-quality bins, *n* = 256; high-quality bins, *n* = 146). Genomes were classified as low quality (completeness ≤ 50% or contamination > 5%), medium quality (90% ≥ completeness > 50% and contamination < 5%) or high quality (completeness > 90% and contamination < 5%). **a**, The number of bins that belong to each of the quality categories and classification of novel SGBs. **b**, Contamination and completeness distribution for the reconstructed genomes. **c**, Distribution of the number of contigs for each of the quality categories. **d**, Distribution of contig N50 values for each of the quality categories. **e**, GTDB-Tk^23^ genus estimation for members of both the novel and the known Mexican SGBs. **f**, GTDB-Tk^23^ species assignment for members of the known Mexican SGBs. In **c**, **d**, data are presented as box plots (middle line, median; lower hinge, first quartile; upper hinge, third quartile; upper whisker extends from the hinge to the largest value no further than 1.5× the interquartile range from the hinge; lower whisker extends from the hinge to the smallest value at most 1.5× the interquartile range from the hinge; data beyond the end of the whiskers are individually plotted outlying points).

**Extended Data Fig. 9:**
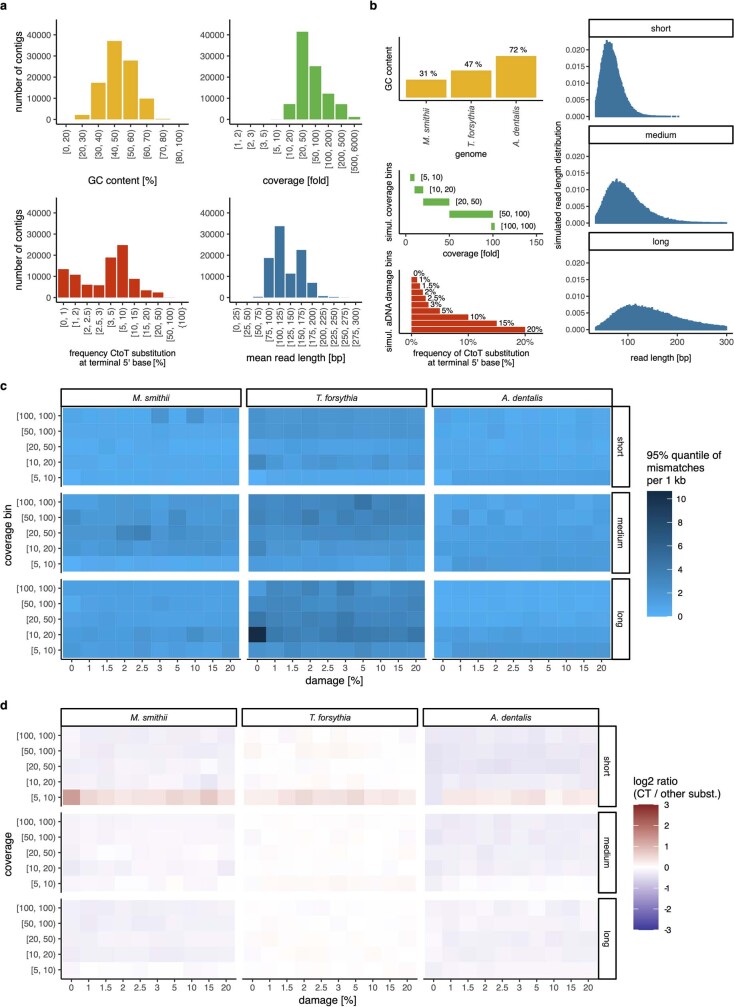
Effect of aDNA damage on the assembly of short-read data. Related to Fig. 2, see Supplementary Information section 6. **a**, Distribution of the values of four sequencing data variables that may have an effect on the assembly of short-read data and were observed in the 498 medium-quality and high-quality MAGs assembled in this study. **b**, Overview of the parameter space of the variables GC content, sequencing depth, observed aDNA damage and read length that was used for simulating short-read sequencing using gargammel^107^. **c**, Number of mismatches per 1 kb of alignable contig sequence with respect to the reference genome as observed at the 95% quantile for all combinations of reference genome, read length distribution, simulated aDNA damage and coverage averaged across the five replicates. **d**, The log_2_-transformed ratio of C-to-T substitutions to the average number of all other substitutions per 1 kb of alignable contig sequence for all combinations of reference genome, read length distribution, simulated aDNA damage and coverage averaged across the five replicates. Positive values indicate an excess of C-to-T substitutions.

The above reference-based analysis identified only taxa present in the database of MetaPhlAn2, which are mostly from industrialized samples. As expected, the palaeofaeces samples have a low percentage of reads mapped to the database (Extended Data Fig. 1f and Supplementary Information section 4). To discover microbial species that were not identifiable using a reference-based approach, we performed de novo genome reconstruction (Methods) from the palaeofaeces and the contemporary Mexican samples (Fig. 2, Extended Data Figs. 6–8 and Supplementary Table 6). Using simulated short-read sequencing data, we show that ancient DNA (aDNA) damage does not significantly affect the simulated assembled genomes (Extended Data Fig. 9 and Supplementary Information section 6).

Following previously used quality-control criteria^13^, we selected medium-quality (90% ≥ completeness > 50%; contamination < 5%) and high-quality (completeness > 90%; contamination < 5%) genomes for a total of 498 genomes from the palaeofaeces samples (Extended Data Figs. 6, 7 and Supplementary Table 6). To exclude contamination with modern DNA, we removed contigs with average read damage of less than 1% on either or both ends of the reads. After this filtering step, 209 medium-quality and high-quality filtered genomes were retained (Extended Data Fig. 6 and Supplementary Table 6).

To determine whether the genomes are gut microorganisms, we measured pairwise genetic distances between the filtered ancient genomes and 388,221 reference microbial genomes (Extended Data Fig. 6a). We labelled each ancient genome as ‘gut’, ‘environmental’ or ‘unsure’ on the basis of the source of isolation of its closest reference genome, and found that 203 out of the 209 filtered genomes are ‘gut’ (Supplementary Table 6), which suggests that there is limited contamination from soil. Out of the 203 filtered gut genomes, 181 are classified as highly damaged (Methods), confirming that they are ancient.

We calculated the pairwise average nucleotide identity (ANI) for the 181 high-damage filtered gut genomes and clustered genomes with more than 95% ANI into SGBs, which resulted in 158 SGBs with one representative genome per SGB (Extended Data Fig. 6a and Supplementary Table 6). SGBs with more than 95% ANI to at least one reference genome were classified as ‘known’ SGBs, and the rest were classified as ‘novel’ SGBs^13^. The results reveal that 61 (39%) of the ancient gut SGBs are novel SGBs (Extended Data Fig. 6a and Supplementary Table 6), 7 of which are shared across multiple palaeofaeces samples. With more than 15% genetic distance from the reference genomes^13^, 18 (11%) of the ancient SGBs belong to novel genera. By contrast, for the Mexican samples, only 1 of the 195 SGBs is novel (Extended Data Fig. 8 and Supplementary Table 6).

We annotated the taxa of the ancient SGBs using GTDB-Tk^23^ and found that the most annotated genera include [*Eubacterium*], *Prevotella*, *Ruminococcus* and *Blautia* (Fig. 2a), which are typical human gut microbiome genera. However, this is an underestimate of the diversity of the SGBs because many could not be confidently assigned to a genus or species. Only 22 genomes were assigned species names (Extended Data Fig. 6f). Results for the 498 pre-filtered bins are shown in Extended Data Fig. 7 and Supplementary Table 6.

To visualize the distribution of the ancient genomes across phylogenies, we built a phylogenetic tree for the high-damage filtered gut bacterial genomes and 4,930 reference genomes that are representative of the human microbiome^13^ (Fig. 2b). The results indicate that the ancient genomes span many human gut microbiome-associated phyla, including Firmicutes, Bacteroidetes, Proteobacteria and Actinobacteria. Phylogenetic trees for *Prevotella* and *Ruminococcus* show that the previously undescribed ancient genomes do not cluster closely with the reference genomes (Supplementary Information section 7). In summary, the 181 reconstructed high-damage ancient microbial genomes belong to various human gut microbiome taxa and include 61 novel SGBs.

## *Methanobrevibacter smithii* tip dating

**Fig. 3:**
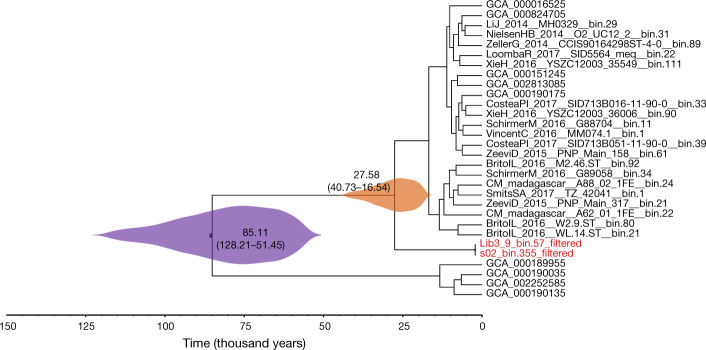
Evolutionary context of a key human gut symbiont. A time-measured phylogenetic tree of *M. smithii* reconstructed on the basis of the core genome using a Bayesian approach under a strict clock model. Purple and orange violin plots illustrate the 95% HPD values (in parentheses) of estimated mean ages for the diversification of *M. smithii* and the split of the lineage leading to ancient *M. smithii* (highlighted in red), respectively.

**Extended Data Fig. 10:**
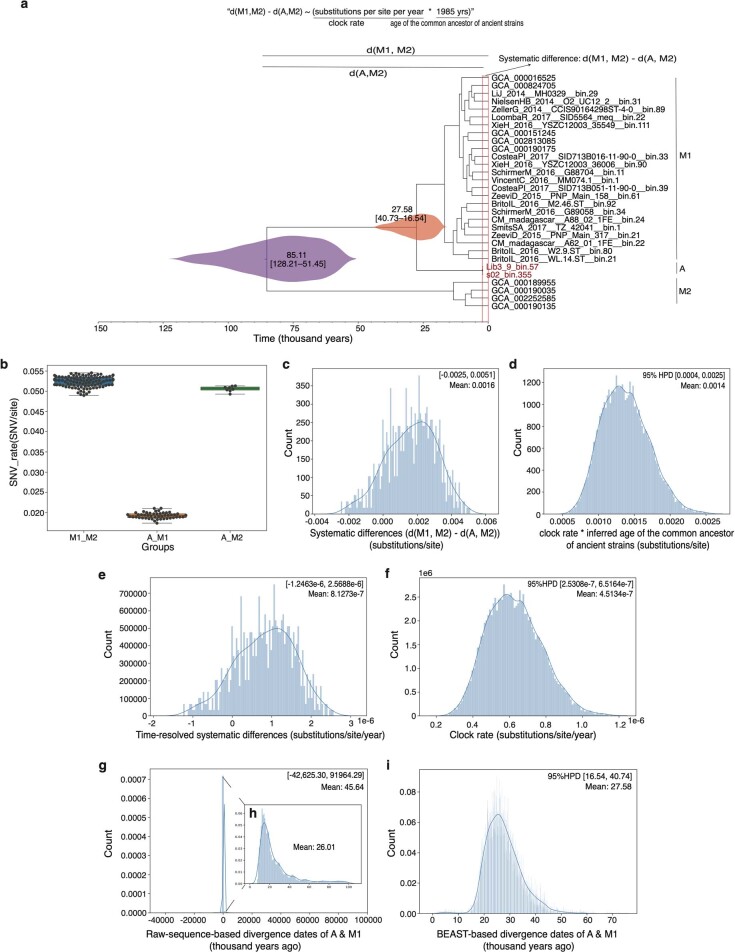
Comparison of *M. smithii* divergence dates from BEAST2 analysis compared with raw genetic distance calculations. Related to Fig. 3, see Supplementary Information section 8. **a**, The different *M. smithii* groups and genetic distances calculated are shown. **b**, Pairwise sequence divergences between M1 and M2 strains (*n* = 96), A and M1 strains (*n* = 48) and A and M2 strains (*n* = 8). Data are presented as box plots (middle line, median; lower hinge, first quartile; upper hinge, third quartile; upper whisker extends from the hinge to the largest value no further than 1.5× the interquartile range from the hinge; lower whisker extends from the hinge to the smallest value at most 1.5× the interquartile range from the hinge; data beyond the end of the whiskers are individually plotted outlying points). **c**, **d**, Comparison of the distribution of systematic differences between M1 and M2 and A and M2 divergences (**c**) and BEAST2 estimates (**d**). **c**, Systematic differences based on pairwise sequence divergences (measured by the single-nucleotide variant rate) between M1 and M2 and A and M2 strains. **d**, Products of the clock rates (substitutions per site per year) inferred using BEAST2^93^ (Supplementary Table 7) and the inferred age of the common ancestor of the ancient strains. **e**, **f**, Comparison of distribution of pairwise time-resolved systematic differences based on raw sequences divergence (**e**) and the distribution of existing inferred clock rates (**f**). **e**, Time-resolved systematic differences calculated by dividing systematic differences (**c**) with the average ^14^C date of the palaeofaeces used in molecular clocking analysis. **f**, Clock rates inferred by BEAST2 analysis (Supplementary Table 7). **g**, Raw-sequence-based divergence dates between A and M1 strains, recalibrated using time-resolved systematic differences. **h**, Distribution of raw-sequence-based divergence dates when low-frequency outliers are excluded. **i**, Distribution of estimated divergence dates between A and M1 strains based on BEAST2 analysis.

Next, we estimated the divergence times of *M. smithii* using two filtered (contigs < 1% damage were removed) ancient *M. smithii* genomes from samples UT30.3 and UT43.2 for tip calibrations (Methods and Supplementary Fig. 3a). Bayesian inference under a strict clock and the most fitting demographic model (Supplementary Table 7) shows that the ancient *M. smithii* genomes fall within the known diversity of contemporary *M. smithii* genomes (Fig. 3 and Supplementary Fig. 3a) and that *M. smithii* began to diversify around 85,000 years ago with a 95% highest posterior density (HPD) interval of 51,000–128,000 years (Fig. 3). This timeline is moderately later than the timeline of its sister species *Methanobrevibacter oralis* (HPD = 112,000–143,000 years)^24^. The two estimates are compatible in terms of HPD overlap, and both occurred within or slightly after the estimated first human migration waves out of Africa around 90,000–194,000 years ago^25,26^. In addition, the origin of the lineage leading to the two ancient *M. smithii* genomes is between 40,000 and 16,000 years ago (mean = 27,000 years ago). These estimates predate (although there is overlap towards the earlier 95% posterior estimates) the accepted age of human entry into North America through the Beringia bridge (20,000–16,000 years ago). The results did not significantly change when potential aDNA damage sites were removed (Supplementary Fig. 3b and Supplementary Information section 8), suggesting that damage did not notably affect our MAGs. We also validated these divergence date estimates using raw sequence divergence calculations (Extended Data Fig. 10 and Supplementary Information section 8). Overall, we show that using ancient genomes for calibrating *M. smithii* phylogenies, we could evolutionarily match previous studies of *M. oralis*^24^. This supports the potential of using ancient MAGs to study the evolutionary history of gut symbionts. However, whether species within the genus actually follow the indicated diversification timeline needs to be investigated with additional ancient *Methanobrevibacter* genomes that span different time periods.

## Functional genomic analysis

**Fig. 4:**
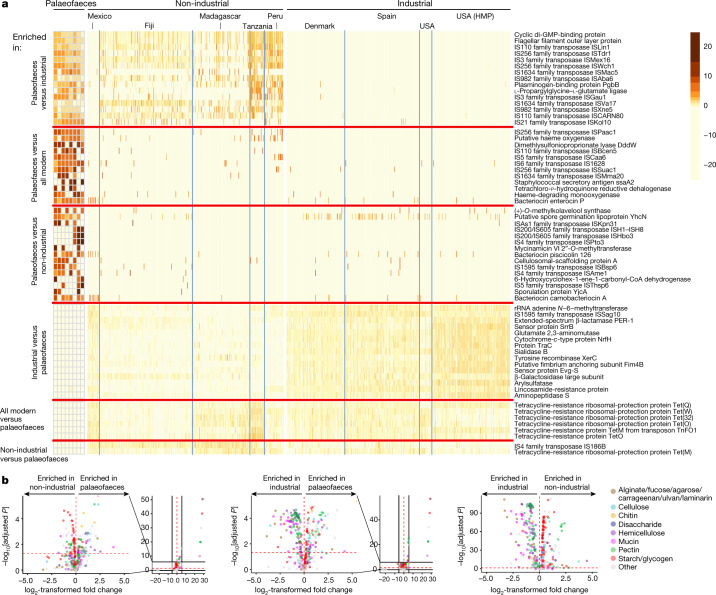
Palaeofaeces exhibit a distinct functional genomic repertoire compared to present-day industrial stool samples. **a**, Heat map of the top-15 genes enriched in the palaeofaeces, industrial and non-industrial samples (complete results in Supplementary Table 8). Functions were annotated using PROKKA^38^ (one-tailed Wilcoxon rank-sum tests with Bonferroni correction). The reads per kilobase per million reads (RPKM) values shown are on a log scale and scaled by row. An unscaled heat map is shown in Extended Data Fig. 12. **b**, Volcano plots showing enriched CAZymes signatures (two-tailed Wilcoxon rank-sum test with FDR correction) comparing palaeofaeces and non-industrial samples (left), palaeofaeces and industrial samples (middle), and non-industrial and industrial samples (right). Each data point represents a CAZy family. CAZymes are colour-coded according to manually annotated broad substrate categories. The horizontal dashed red line indicates adjusted *P* = 0.05. The vertical dashed red line indicates log_2_-transformed fold change = 0. For the left and middle plots, both the entire dataset and a magnified version are shown. For the right plot, the *x*-axis limits were set to −5 and 5 (as a result, eight statistically non-significant CAZymes were removed).

Our functional genomic analysis (Methods) reveals that the palaeofaeces are enriched in transposases (Fig. 4a, Supplementary Tables 8, 11 and Supplementary Information section 9) relative to industrial (two-tailed Fisher’s test, *P* = 3.2 × 10^−9^) and non-industrial samples (*P* = 3.2 × 10^−13^). Transposases are also enriched in the non-industrial samples relative to the industrial samples (*P* = 3.0 × 10^−9^).

**Extended Data Fig. 11:**
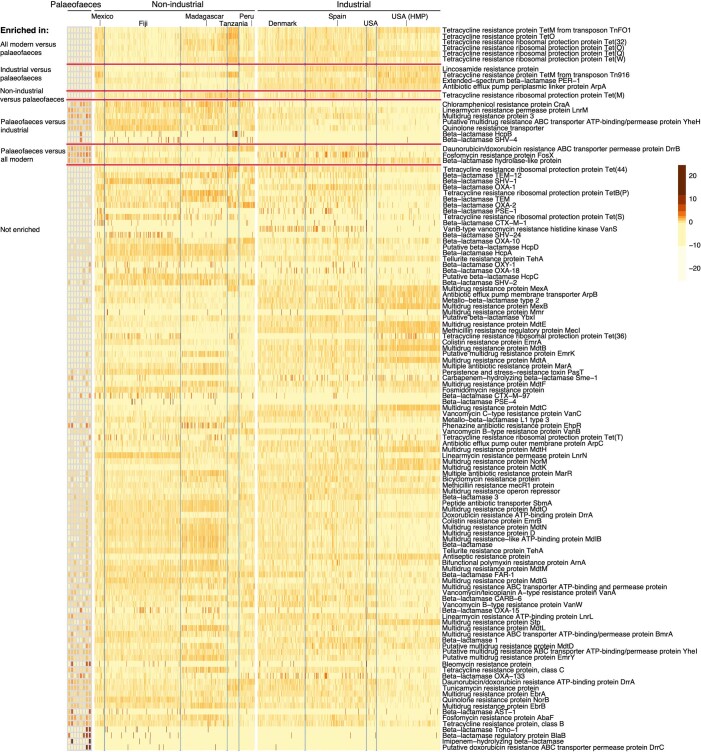
Heat map of 120 antibiotic-resistance genes found in the palaeofaeces, industrial and non-industrial samples. Related to Fig. 4. Functions were annotated using PROKKA^38^ with the UniProtKB database^108^. Enriched genes were identified using one-tailed Wilcoxon rank-sum tests with Bonferroni correction. Non-enriched genes were sorted by fold change. RPKM values are shown on a log scale and scaled by row.

**Extended Data Fig. 12:**
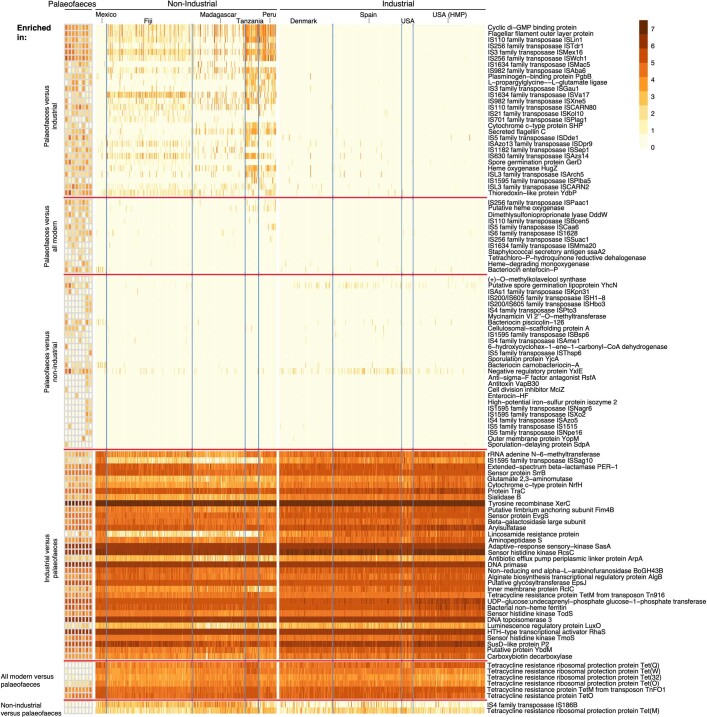
Heat map of the top-40 genes enriched in the palaeofaeces, the industrial and the non-industrial samples. Related to Fig. 4, complete results are provided in Supplementary Table 8. Functions were annotated using PROKKA^38^ with the UniProtKB database^108^. Enriched genes were identified using one-tailed Wilcoxon rank-sum tests with Bonferroni correction. RPKM values are shown on a log scale without scaling.

On the other hand, both the industrial and the non-industrial samples are enriched in antibiotic-resistance genes (many of which are tetracycline-resistance genes) relative to the palaeofaeces (Fig. 4a, Extended Data Fig. 11 and Supplementary Table 8), consistent with the palaeofaeces being dated to the pre-antibiotic era^27^. In the present-day samples, multiple tetracycline-resistance genes are present in *Streptococcus mitis* and *Collinsella* SGBs (Supplementary Information section 10). Our analysis suggests that these tetracycline-resistance genes are encoded chromosomally rather than on plasmids (Supplementary Information section 11). Moreover, several glycan degradation genes (endo-4-*O*-sulfatase and three SusD-like proteins) are enriched in the industrial samples compared to the palaeofaeces (Extended Data Fig. 12 and Supplementary Table 8). These genes are mostly found in Bacteroidetes SGBs, including *Bacteroides* and *Prevotella* species (Supplementary Information section 10).

Analysis of CAZymes (carbohydrate-active enzymes)^28^ reveals similar enrichment patterns in the palaeofaeces and the non-industrial samples compared to the industrial samples (Fig. 4b). For instance, starch- and glycogen-degrading CAZymes are enriched in the palaeofaeces and the non-industrial samples, whereas mucin- and alginate-related CAZymes are enriched in the industrial samples. Chitin-degrading CAZymes are enriched in the palaeofaeces relative to both the non-industrial and industrial samples. This is in accordance with our microscopic dietary analysis that identified chitin sources (*Ustilago maydis*, mushrooms and insects) in the palaeofaeces (Supplementary Information section 2). These foods were commonly part of ancient Pueblo and Great Basin diets^29^. These chitin CAZymes are prevalent in MAGs within Oscillospiraceae, Lachnospiraceae and Clostridiaceae families (Supplementary Information section 10). Taken together, the palaeofaeces share more features with non-industrial samples than with industrial samples.

## Discussion

To date, it is not known to what extent the human microbiome has evolved over long time spans. Our analysis supports that present-day non-industrial human gut microbiomes more closely resemble the palaeofaeces, whereas the industrial gut microbiome has diverged from the ancient gut microbiome. Some species, such as *Ruminococcus callidus*, *Butyrivibrio crossotus* and *T. succinifaciens*, are more prevalent in the palaeofaeces and non-industrial samples than industrial samples (Fig. 1c and Supplementary Table 3). Furthermore, the industrial samples are enriched in mucin-degrading genes (Fig. 4) that are mostly found in our *Bacteroides* and *Prevotella* SGBs (Supplementary Information section 10). This is in line with the higher abundance of Bacteroidetes in the industrial samples (Fig. 1a), previous findings that members of the Bacteroidetes phylum possess many glycan-degrading genes^30^ and the enrichment of mucin-using enzymes in the industrialized gut microbiome^1^. By contrast, the palaeofaeces and the non-industrial samples are enriched in starch- and/or glycogen-degrading CAZymes (Fig. 4b; probably because of a higher consumption of complex carbohydrates relative to simple sugars) and mobile genetic elements (Fig. 4a). This is in agreement with a previous observation of a higher abundance of mobile genetic elements in agrarian Fiji islanders compared to North American individuals^31^. Our finding supports the hypothesis that mobile genes are important for the colonization of the gut of non-industrial populations, perhaps for adaptation to an environment with greater variation, such as seasonal variation^1^.

Moreover, we report the reconstruction of 181 authenticated ancient gut microbial genomes, 39% of which are novel SGBs (Fig. 2 and Extended Data Fig. 6). The highly degraded nature of aDNA is an obstacle to recovering MAGs from ancient samples. However, a recent study indicates that MAG recovery from mammalian dental calculus is possible with deeper sequencing^32^. Here, we show that large-scale de novo assembly and recovery of previously undescribed microorganisms from palaeofaeces are attainable. The reconstructed ancient microorganisms are of high quality and could be used for phylogenetic analysis and tip-based dating (Figs. 2b, 3), shedding light on the evolutionary relationships between the ancient genomes and their modern relatives. These analyses were possible due to the extraordinary preservation of the palaeofaeces, use of aDNA extraction methods suited for palaeofaeces^33^, high sequencing depth (100,000,000–400,000,000 read pairs per sample) and advances in de novo genome reconstruction methodology^13^.

Although long DNA fragments are usually excluded from aDNA analysis, our findings suggest that some well-preserved palaeofaeces contain longer DNA fragments. Preservation of aDNA in palaeofaeces is relatively understudied, and known kinetics of DNA damage is largely based on mineralized tissues^34–36^. Post-mortem decomposition of DNA is driven by the presence of water and because palaeofaeces are preserved only under extreme cases of desiccation or freezing with the absence or immobilization of water^33^, they are expected to exhibit lower levels of hydrolytic damage. Furthermore, there is variation in the preservation of DNA across archaeological sites^37^. Palaeofaeces from Zape are known to have well-preserved aDNA^6,14,15^. Two of our palaeofaeces samples were from Boomerang Shelter, which is further north compared to Zape. The extreme aridity and lower temperature of the site probably contributed to the preservation of the samples. In addition, seasonality is relevant to the decomposition of palaeofaeces^37^. Microbotanical analysis reveals that most of the palaeofaeces from Boomerang Shelter were deposited in the spring, summer or autumn, except for UT30.3, which was deposited in late autumn or early winter (Supplementary Table 2). This is the ideal environment for preservation owing to lack of decomposers^37^ and might explain the low damage levels of UT30.3.

In this study, we establish that palaeofaeces with well-preserved DNA are abundant sources of microbial genomes, including previously undescribed microbial species, that may elucidate the evolutionary histories of human microbiomes. Similar future studies tapping into the richness of palaeofaeces will not only expand our knowledge of the human microbiome, but may also lead to the development of approaches to restore present-day gut microbiomes to their ancestral state.

## Methods

### Data reporting

No statistical methods were used to predetermine sample size and the experiments were not randomized. Metagenomic library construction, dietary analysis and seasonality interpretation were performed blindly. Blinding is not applicable to the metagenomic analysis; all samples were analysed computationally in a uniform manner.

### Archaeological samples and sites

The eight palaeofaeces analysed in detail were collected from Boomerang Shelter, Arid West Cave and Zape as described below. Three soil samples were collected from Boomerang Shelter. Palaeofaeces from Boomerang Shelter are curated at the Edge of the Cedars State Park Museum, Blanding, Utah, USA. Samples from Arid West Cave are curated at The Robert S. Peabody Institute of Archaeology, Andover, Massachusetts, USA. The collection from Zape is curated at the Anthropology Department of the University of Nevada, Las Vegas, USA.

All samples are from dry rock shelters, sometimes called caves or alcoves. These are neither dark nor deep but have naturally eroded openings in the sides of cliffs that are only tens of metres wide at most. However, the palaeofaeces remain dry with exceptional preservation. Such rock shelters often even preserve feathers and other such material after a thousand or more years. Palaeofaeces, once deposited, would have been covered by windblown soil or human activity. As these shelters were used repeatedly over many years, some palaeofaeces could have been re-exposed and moved beyond the dry portion and become wet then once again moved and dried; or in a dry location exposed to dumped cooking water and so on. Those palaeofaeces samples seemed to have considerable evidence of fungi based on macroscopic evidence. Thus, we included only samples that do not appear to have been negatively affected by such events. Furthermore, such post-depositional movement can change the initial stratigraphic location of the specimens. We carbon-dated using ^14^C dating all of the palaeofaeces samples and they were dated to anticipated dates (Extended Data Fig. 1b and Supplementary Table 1).

#### Boomerang Shelter

This shelter lies in southeastern Utah^39^. The primary occupation was during Basketmaker II times, but a few pre-farmer artefacts dating to as early as 8310 years before present (bp) (around 7400 bc) have been recovered. However, most remains dated to between 2500 and 1500 bp and two of our samples dated to the first century ad in the middle of this range. By this time, the inhabitants were committed maize farmers with high proportions of maize in their diet as demonstrated by a previous study of palaeofaeces from the shelter^40^. Furthermore, the site is only about 40 km from the contemporary Turkey Pen Ruin, palaeofaeces from which yielded similar dietary results and had good preservation of human, plant and animal aDNA, but bacterial DNA was not considered for this site^41^.

#### Arid West Cave

The precise location of this set of samples cannot be determined (samples labelled AW107, AW108, AW110A, and so on) as they are without location labels. The samples were found at a time before palaeofaeces were regularly collected and saved, and if saved they were never studied. We know these samples were collected in 1931 or a year or two before, which narrows the possibilities of where they are from. The radiocarbon dates and macro-remains (diet) of these palaeofaeces make clear that they are from the northern part of the American Southwest, but they could come from several different expeditions almost a century or more ago. There is a remote possibility that they come from an expedition mounted by the Peabody Museum of Archaeology and Ethnology at Harvard University. They could be from the Samuel Guernsey projects between 1920 and 1923^42^. However, none of the project records make any mention of palaeofaeces, nor do they fit the time frame and site types that he studied. Conversely, the Harvard Peabody Museum also undertook a series of expeditions to eastern parts of Utah between 1928 and 1931 (often referred to as the Claflin–Emerson or Morss projects) and they did recover palaeofaeces and did work in deposits of the appropriate time, in particular at the Rasmussen Ranch Cave site in east-central Utah^43–45^. This is the most likely source, but it cannot be confirmed absolutely. Fortunately, for our purposes, the exact location is not critical. Knowing the time frame and general region is adequate for our purposes. The palaeofaeces are some 500 years or more closer to the present than those from Boomerang Shelter. The major difference is that these individuals would have had maize as a staple of their diets for an additional 500 years.

#### La Cueva de los Muertos Chiquitos (Zape)

The La Cueva de los Muertos Chiquitos site (ad 660–1430) is located near Zape, just north of Durango, Mexico (hereafter Zape). Excavated in the 1950s by Sheilagh and Richard Brooks, the cave primarily dates to the Gabriel San Loma cultural phase. The site is known for what appears to be a deliberate burial of a series of infants who died at or about the same time^46^. However, the palaeofaeces in our sample came from a different layer in the cave and are not associated with that event. Our samples date from the 700s ad to the early 900s ad. No full report exists, but various aspects of the material have been published^46–49^.

### ^14^C dating

The palaeofaeces samples were submitted to DirectAMS for accelerator mass spectrometry radiocarbon dating measurements. As shown in Extended Data Fig. 1b and Supplementary Table 1, all dates fit with the known dates of the sites that the samples are from and are dated to the first ten centuries ad.

#### Dietary analysis

Our knowledge of the diets comes from the macro-remains analysis of the palaeofaeces plus archaeologically recovered information from these and similar shelters in the region. The diet of the individuals has been summarized as maize and other available remains (Supplementary Information section 2 and Supplementary Table 2). Beans were not present for the inhabitants of the Boomerang Shelter and were a recent introduction for inhabitants of Arid West Cave, but had been present longer and with more varieties for the inhabitants of Zape cave. Wild plants would have included grasses and pinyon pine nuts, cactus, and agave and relatives, including the fruits, flowers and fleshy parts. Animals would have included deer and various rabbits, other mammals including a variety of rodents, as well as insects such as locusts and cicadas, both adult and larval stages, reptiles such as snakes, and birds. For most periods, the absence of beans would have required substantial animal protein.

#### Extraction, library preparation and sequencing of aDNA

Samples were sent to the Molecular Anthropology Laboratory at the University of Montana, which is a controlled access facility, wherein researchers are required to wear Tyvek clean suits, foot coverings, hair nets, face masks, arm coverings and gloves to enter. All work surfaces in the room, including specialized clothing, are bleached daily using a 50% household bleach solution and between each sample processing. Additionally, UV light overhead is run for an hour each evening, as well as a smaller targeted light on work surfaces, to aid in decontamination. The room maintains a positively pressurized environment. Movement from a laboratory working with post-PCR products to the aDNA laboratory was not allowed at any time.

Samples were transferred to the University of Montana in conical tubes, and after the outside had been wiped down with a bleach solution, a small portion was scraped from the centre of the sample into a UV-irradiated (for a minimum of 15 min) 15-ml sterile tube. Soil samples were weighed out in sterilized weigh boats. Approximately a gram was taken from soil and faecal samples and 5 ml of EDTA (0.5 M, pH 8) was added to each. Samples were incubated at room temperature for approximately 48 h, after which 20 μl of 1 mg ml^−1^ proteinase K was added to each, followed by sealing with Parafilm and further incubation at 52 °C with slow rotation (4 rpm) for 4 h. Once the samples were removed from incubation, they were extracted following a previously published protocol^50^. This entailed spinning the sample to the bottom of the tube by centrifugation at 1,500*g* and 1.5 ml of the EDTA solution being pipetted into a sterile, UV-treated 15-ml polypropylene tube. Next, 13 ml of PB buffer (Qiagen) was added to each sample and mixed by inversion. The liquid was spun through Qiagen MinElute filters using 50-ml polypropylene tubes and nested conical reservoirs (Zymo) with attached filters. These filters were then removed, placed into a collection tube, washed twice with PE buffer (Qiagen) and eluted with two 50 μl DNase-free H_2_O rinses into sterile, low-bind 2-ml tubes. A blank negative control was run through all of the previous and following steps, and in no instance was contamination in subsequent DNA quantifications or analyses detected.

Library preparation was completed using previously published protocols^51,52^. This entailed using half of the extracted DNA to perform uracil DNA glycosylase (UDG) repair with the USER enzyme (Supplementary Information section 12 and Supplementary Table 10). The other half of the extract was taken straight to blunt-end repair, followed by adaptor ligation and fill-in. Both the UDG-treated and untreated samples were separately indexed using a dual-index process with indexes from previously published studies^53,54^. The sample concentration was then calculated using a Qubit 4 with the High Sensitivity DS DNA assay (ThermoFisher). Samples with more than 1 ng μl^−1^ were pooled and sent for sequencing via overnight FedEx. Libraries were sequenced on the Illumina HiSeq 4000 platform in 2 × 150-bp paired-end format.

### Overview of the present-day samples

The present-day samples were classified into two categories: present-day industrial samples and present-day non-industrial samples. An industrial lifestyle is defined here as one with consumption of a Western diet, common antibiotic use and sedentary lifestyle. Non-industrial lifestyle is characterized by consumption of unprocessed and self-produced foods, limited antibiotic use and a more active lifestyle.

In total, 789 present-day human gut metagenomes were analysed. Present-day industrial samples encompass metagenomes from 418 stool samples, including 169 individuals from the USA (147 from the HMP^55^ and 22 from a previously published study^4^), 109 from Denmark^56^ and 140 from Spain^56^. Present-day non-industrial samples include publicly available gut metagenomes of 174 individuals from Fiji^31^, 36 from Peru^4^, 112 from Madagascar^13^ and 27 from Tanzania^57^. In addition, stool samples from 22 individuals were collected from a Mazahua community in the centre of Mexico. They preserve a non-industrial lifestyle and have remained semi-isolated from urban areas. The affinity to a non-industrial Mexican diet was assessed by the application of a questionnaire about the frequency of consumption of fresh or industrial food, which was adapted from a previous study^58^. The definition of a non-industrial Mexican diet is one that provides protein, carbohydrates, vitamins and minerals from the consumption of foods such as maize, legumes (mainly beans), fruits, vegetables such as pumpkins and nopales, as well as different types of herbs such as quelites and verdolagas^58^. These individuals had not received antibiotic treatment in at least six months before sample collection. All study participants were recruited in accordance with a human participant research protocol (IRB number: CEI 2018/01) approved by the Institutional Review Board of INMEGEN. Each participant provided a statement of informed consent, and we have complied with all of the relevant ethical regulations.

### Extraction, library preparation and sequencing of modern DNA

Stool samples from the individuals of Mexican ancestry were immediately put in dry ice after collection and sent to the Joslin Diabetes Center for processing. DNA extraction was performed using ZymoBIOMICS DNA Miniprep Kit (D4300). Sample concentrations were calculated using a Qubit 3.0 with the High Sensitivity DS DNA assay (ThermoFisher) and purity was assessed using a NanoDrop Spectrophotometer.

Library preparation was performed following a previously published protocol^59^. Sample concentrations were again calculated using a Qubit 3.0 with the High Sensitivity DS DNA assay (ThermoFisher). Samples were pooled for a total of 11 samples per lane and sent for shotgun metagenomic sequencing via overnight FedEx. Libraries were sequenced on the Illumina HiSeq 4000 platform in 2 × 150-bp paired-end format.

### Read processing and quality control

Adapters were removed from paired Illumina reads using AdapterRemoval v.2^60^. Human DNA sequences were filtered out using KneadData v.0.6.1 (https://github.com/biobakery/kneaddata) by mapping reads to the *Homo sapiens* reference database (build hg19)^61^. For the archaeological samples, short reads of fewer than 30 bp were removed using Cutadapt (v.2.8)^62^. All downstream analyses were done on these pre-processed reads unless otherwise specified.

### Human DNA analysis

In this study, we performed shotgun metagenomic sequencing, which also gave us access to the human host DNA. Although we did not perform targeted enrichment of human DNA molecules, the small amount of randomly sequenced molecules that could be aligned to the human reference genome was large enough to authenticate the host of the faecal samples as human and not another organism, such as a dog (as the two can be confused morphologically). These data further enabled us to investigate whether their mitochondrial haplogroups overlapped with the ones expected in the geographical region during the lifetime of the individuals. The human genetic data were not the target of the sampling process nor the research being undertaken and were used only to verify the microbial results. All of the human DNA analysis was performed before removal of human DNA by KneadData.

Owing to the high copy number of human mtDNA, almost complete inheritance on the maternal lineage and lack of recombination^63^, we used human mtDNA from the low-coverage human data to infer the proportion of modern human contamination and for haplogroup identification. For the contamination estimate based on the observed minor allele frequencies at rarely polymorphic sites, we used contamMix (v.1.0-10)^64^ as part of the ancient mtDNA pipeline of mitoBench v.1.6-beta (https://github.com/mitobench/mitoBench and https://github.com/alexhbnr/mitoBench-ancientMT). For haplogroup identification, reads were mapped to the human mtDNA reference genome (rCRS)^65^ and duplicates were removed using Picard MarkDuplicates v.2.18.2 (https://broadinstitute.github.io/picard), followed by a left alignment to normalize indels. A Bayesian approach to variant analysis was performed using FreeBayes (v.1.1.0)^66^ and haplogroups were identified by inputting the variant calling file into HaploGrep (v.2.1.21)^67^. All steps for haplogroup identification were run through a custom-made workflow in Galaxy (2019 build version)^68^ alongside command line executions for validation and replication.

### Reference-based taxonomic classification

Reference-based taxonomic classification for the ancient, Mexican and Fijian samples was performed by running MetaPhlAn2 (v.2.7.5) on the pre-processed reads using default settings^20^. For the other present-day industrial and non-industrial samples, MetaPhlAn2 output files were collected from the R package curatedMetagenomicData (v.1.16.0)^69^. One sample from Fiji (SRS476326)^31^ was 100% unclassified and was excluded from the reference-based taxonomic analysis.

### Prediction of the source of microbial communities

To predict the source of each sample, the species composition (from MetaPhlAn2) of the palaeofaeces was compared to 40 industrial gut microbiome samples, 40 non-industrial gut microbiome samples and a diverse set of environmental samples (Supplementary Table 9). These environmental samples include the 3 soil samples collected in this study, 40 Pleistocene sediment samples^70^ and 7 Holocene human-associated sediments (which overlap in age with our palaeofaeces) from CoproID^71^. MetaPhlAn2 results for 40 industrialized and 40 non-industrialized human participants were obtained from the R package curatedMetagenomicData^69^ (Supplementary Table 9). The rest of the samples were run through MetaPhlAn2^20^ using default settings, then converted to biom format. The resulting species abundance matrix biom file was used as input for SourceTracker2^72^.

### Host source prediction

To predict whether the source species of each palaeofaeces was *H. sapiens* or *Canis familiaris*, pre-processed reads were run through CoproID (v.1.0)^71^ using the following settings: --genome1 GRCh37 --genome2 CanFam3.1 --name1 ‘Homo_sapiens’ --name2 ‘Canis_familiaris’.

### Parasite analysis

Paired reads were fused into single reads using bbmerge from BBSuite (v.38.24)^73^ using standard parameters. Classification of the fused reads against a custom nucleotide database was performed using Kraken 2 (v.2.0.8-beta)^74^ using a threshold of 0.15. The custom Kraken 2 database was created from 160,946 publicly available genomes from RefSeq for bacteria, fungi, plants, mammalian vertebrates, other vertebrates and viruses (May 2019). In addition, 530 genomes were selected from 926 available protozoa, flatworm and roundworm genomes downloaded from GenBank (May 2019). The 530 genomes were selected based on assembly criteria, including N50, number of contigs and number of ambiguous sequences as described previously^75^. Contigs with length less than 1,000 bp were removed. For protozoa, flatworm and roundworm genomes, artificial nodes in the taxonomic tree were introduced. This means that below species or strain level, we have included further nodes for assembly and contig levels to increase the resolution of classification. To minimize the number of false-positive classifications, we used three different cut-offs in the Kraken-2-based analysis. Parasite species with hits below 1,000 reads were removed. To ensure that the hits were dispersed over the genome, we also required that the number of contigs with at least one hit was more than 10% of all of the contigs in the assembly and that the combined length of the contigs with hits represented at least 50% of the whole genome. Coverage of the genome and dispersion of reads were visually inspected for each candidate (Supplementary Table 4).

### De novo assembly pipeline

Each sample was de novo assembled into contigs using MEGAHIT (v.1.2.9)^76^ with default settings. Assembly statistics (number of contigs, number of bp in contigs, contig N50, contig L50 and the longest contig) were calculated using the statswrapper.sh function from BBMap (v.38.86) (https://sourceforge.net/projects/bbmap/) with default parameters (Supplementary Table 1).

### Genome reconstruction

Ancient and Mexican genomes were reconstructed as previously described^13^. Pre-processed reads were de novo assembled into contigs using MEGAHIT (v.1.2.9)^76^. For each sample, reads were mapped to contigs using Bowtie 2 (v.2.3.5.1)^77^ with default settings (no minimum contig length). The resulting alignment file was sorted and indexed with SAMtools (v.1.9)^78^. The sorted BAM file was used for contig binning using MetaBAT 2 (v.2.12.1)^9^ with default parameters (minimum contig size = 2.5 kb), resulting in putative genomes. Quality controls (completeness, contamination, genome size (bp), number of contigs, contig N50 values, mean contig length and the longest contig) were assessed using the lineage-specific workflow in CheckM with default settings (v.1.0.18)^79^. Following recent guidelines^80^, genomes with completeness between 50% and 90% and contamination < 5% were classified as medium-quality genomes. Higher-quality genomes with completeness > 90% and contamination < 5% were classified as high-quality genomes. Coverage for each contig was calculated using the ‘coverage’ command in CheckM^79^, and coverage per genome was calculated by averaging the coverage profiles across all contigs within the genome.

The relative abundance of each reconstructed genome (Supplementary Table 6) was calculated by dividing the number of reads aligned to the genome by the total number of raw reads from that sample. On average, the medium-quality and high-quality filtered genomes account for 11.5% (s.d. = 9.4) of the total raw reads per sample (Supplementary Table 6), and the novel medium-quality and high-quality filtered genomes constitute 3.3% (s.d. = 1.7) of the total raw reads per sample (Supplementary Table 6). To calculate the percentage of contigs binned in each genome, the number of contigs per genome was divided by the number of contigs binned from the sample. To calculate the percentage of bp from contigs binned in each genome, the genome size (in bp) was divided by the number of bp in the contigs binned from the same sample. The percentages across genomes from the same sample were summed to calculate the percentage per sample.

To cluster assembled genomes of the same species, pairwise ANIs for the assembled genomes were calculated using the ‘dereplicate’ command in dRep (v.2.4.2)^81^ with the following settings: -comp 50 -pa 0.9 -sa 0.95 -nc 0.30 -cm larger. This dRep command uses MUMmer (v.3.23)^82^ to cluster genomes with more than 95% ANI together into a SGB and select one representative genome per SGB. This 5% distance metric follows the definition of a bacterial species^83^.

To determine whether each of the SGBs belongs to a known microbial species, pairwise genetic distances were calculated between each of the representative genomes and each of the 388,221 reference microbial genomes. The reference genomes included previously reconstructed human gut MAGs^11,12^ (as previously catalogued^84^), previously reconstructed MAGs^13^, 80,990 genomes from the NCBI GenBank database previously used as reference^13^, and MAGs from nonhuman primate gut metagenomes^85^. Mash distances were calculated using Mash (v.2.1)^86^ for all of the genomes using default settings (sketch size = 1000). Subsequently, ANIs were calculated using FastANI (v.1.3)^83^ for each ancient genome and its 100 closest reference genomes within 10% Mash distance. The ‘cluster’ command in dRep^81^ was used to run FastANI^83^ using the default alignment fraction (0.1) and with the following settings: -sa 0.95 --S_algorithm fastANI. Bins with more than 95% ANI with at least one reference genome were classified as ‘known’ SGBs and the rest were classified as ‘novel’ SGBs. Each bin was labelled as ‘gut’, ‘environmental’ or ‘unsure’ on the basis of the source of its closest reference genome (that is, if the closest reference genome was a MAG or an isolate from a gut microbiome sample, then the bin was labelled as ‘gut’). The ‘classify’ workflow in GTDB-Tk (v.0.3.0; default settings) was used to assign taxa to the bins^23^.

### Damage pattern assessment

Assessment of host DNA damage was performed by mapping reads (before removal of human DNA by KneadData) to the human mtDNA reference genome (rCRS)^65^ and inputting the alignment files into mapDamage2.0 (v.2.0.9)^87^. Damage patterns for microbial DNA were assessed with DamageProfiler (v.0.4.7)^88^ using each of the medium-quality and high-quality reconstructed genomes as reference for its respective sample. For each genome, reads were mapped to each contig, the resulting alignment file was sorted and indexed with SAMtools (v.1.9)^78^, DamageProfiler^88^ was run per contig, and the average damage levels and damage variation across reads per contig were calculated. The 498 medium-quality and high-quality assembled genomes from the palaeofaeces were further curated by removing contigs with average read damage < 1% at either or both ends of the reads. This is a conservative cut-off because the process removed some known gut bacterial species (for example, *T. succinifaciens*) from the medium-quality and high-quality bins (Extended Data Fig. 7g). Genomes were classified as having high damage if the average damage level at the ends of the reads was within the top 50th percentile damage level among the 498 medium-quality and high-quality bins. Genomes were classified as having high damage variance if the s.d. of the damage at the ends of the reads was within the top 50th percentile s.d. among the 498 medium-quality and high-quality bins. Genomes with high damage levels and low damage variance are our most confident ancient genomes because most of the contigs in these genomes are highly damaged, hence they must contain minimal to no contamination with modern DNA.

### Phylogenetic analysis

To build phylogenetic trees, the ‘classify’ workflow in GTDB-Tk (v.0.3.0; default settings) was used to identify 120 bacterial marker genes and build a multiple sequence alignment based on these marker genes^23^. The resulting FASTA files containing multiple sequence alignments of the submitted genomes (align/<prefix>.[bac120/ar122].user_msa.fasta) were used for maximum likelihood phylogenetic tree inference using IQ-TREE (v.1.6.11)^89^ with the following parameters: -nt AUTO -m LG. Newick tree output files were visualized with iTOL v.5 (https://itol.embl.de/).

For Fig. 2b, 4,930 representative human microbiome genomes that were previously reconstructed^13^ were used as reference genomes. For Supplementary Fig. 1, all genomes from the NCBI RefSeq database belonging to each genus were used as reference genomes. Ancient genomes included in the trees were bacterial genomes from the 181 high-damage bins that were assigned to each genus. Multiple sequence alignment files used to create the phylogenetic trees were visually inspected (Supplementary Fig. 2).

### Divergence estimates of *M. smithii*

To calibrate the *M. smithii* phylogeny, we used as tip dating two *M. smithii* genomes reconstructed from ancient metagenome samples UT30.3 (1947 ± 30 bp) and UT43.2 (1994 ± 26 bp). We selected *M. smithii* because of its presence in two distinct palaeofaeces samples, a large number of available modern genomes, and a previous divergence estimate in the genus *Methanobrevibacter* that could be used as a comparison^24^. We first studied the phylogenetic placement of these two ancient genomes by leveraging 488 contemporary *M. smithii* genomes, and inferring a high-resolution phylogeny composed of ancient and contemporary genomes using PhyloPhlAn (v.3.0)^13,90^. Twenty-eight contemporary *M. smithii* genomes that were representative of the *M. smithii* phylogenetic expansion were selected for further analysis, along with the two ancient genomes, compiling a dataset of 30 genomes (Supplementary Fig. 3). To build this dataset, orthologues were searched within the ancient genomes (*n* = 2) and their contemporary counterparts (*n* = 28) and were merged into one concatenated alignment with a length of 346,567 bp using Roary (v.3.13.0)^91^ with parameters -i 0.95 and -cd 90. To assess the certainty of core genome phylogeny of the 30 *M. smithii* genomes, we used RAxML (v.8.1.15)^92^ under a GTR model of substitution with 4 gamma categories and 100 bootstrap pseudo replicates. BEAST2 (v.2.5.1)^93^ was used to infer the divergence times between genomes using a GTR model of substitution with 4 gamma categories. Convergence of posteriors was assessed by visualizing the log-transformed files with Tracer (v.1.7)^94^. Following a previous divergence estimate of *Methanobrevibacter*^24^, we used a strict clock model in BEAST2, and further performed model selection (Supplementary Table 7) to choose the most fitting demographic (tree) prior. We estimated the marginal likelihood via path sampling and stepping stone for five demographic models. We ran the chains up to 297 million generations to obtain convergence in accordance with the effective sample size of all parameters being over 200. We identified a coalescent Bayesian skyline^95^ as the most fitting demographic model for our dataset (Supplementary Table 7), indicating that the genomes are evolving under Wright–Fisher dynamics^96^. We further tested relaxed clocks, but the effective sample size of most parameters (including the prior and the root age, the latter of which varied by 2–3 orders of magnitude) were extremely low even after 500 million generations (more than 2-week running time). Moreover, the posterior mean, although not at convergence, was in the range of 10^−5^–10^−6^ mutations per site per year, a rate that is incompatible with the mutation rates of bacteria over a time range higher than 100 years^97^. As various posteriors could not go to convergence after sufficient sampling and/or were not compatible with known patterns of bacterial evolution in realistic scenarios (Supplementary Table 7), we focused on the strict clock model.

We optimized our molecular clock analysis by ruling out possible artefacts that could be derived from aDNA degradation. Post-mortem DNA damage results in an elevated C-to-T substitution rate at the 5′ end of reads (and an elevated G-to-A substitution rate at the 3′ end of reads)^98^. To mitigate such bias, we repeated our BEAST2 analyses using genomes reconstructed from reads that aligned to the two ancient *M. smithii* genomes but had been trimmed at the first and last 5 bp using Cutadapt (v.2.8)^62^. To further inspect substitutions that could possibly be derived from aDNA damage, we searched the alignment for polymorphic positions at which all contemporary genomes had C/G as base and all ancient genomes had T/A as base. We visually assessed the pileup of reads on the ancient MAGs using Tablet (v.1.19.09.03)^99^ and observed that 24 suspicious substitutions were located at the end of reads, suggesting that these sites could be prone to aDNA degradation. To minimize the effect of strain heterogeneity on the clocking analysis, we removed arbitrary sites of genomes that polymorphism dominance of mapped reads was lower than 0.8. Having identified and removed 11,938 sites, we obtained a carefully curated genome alignment with a length of 339,321 bp. This dataset was analysed using the most fitting demographic model under a GTR + G replacement model and a strict clock model (Supplementary Table 7).

### Molecular function analysis

From contigs, genes were annotated with PROKKA (v.1.14.6)^38^ with default parameters per sample. A non-redundant gene catalogue combining all of the predicted genes across all samples was generated with CD-HIT-EST (v.4.8.1)^100^ with a 95% identity threshold using the following settings: -n 10 -c 0.95 -s 0.9 -aS 0.9. Genes labelled as ‘hypothetical protein’ were removed from the gene catalogue. Raw reads from each sample were aligned to the gene catalogue using Bowtie 2 (v.2.3.5.1)^77^ with the following parameters: -D 20 -R 3 -N 1 -L 20 -i S,1.0,50 --local --mm. The output BAM file was sorted and indexed with SAMtools (v.1.9)^78^. For each gene per sample, the relative abundance was calculated by dividing the number of reads aligned to the gene by the length of the gene and the total number of reads aligned to the gene catalogue per sample. RPKM values were calculated by multiplying the relative abundance values by 1,000 (for the per kb conversion) and 1,000,000 (for the per million conversion). Five samples from Madagascar (SRR7658580, SRR7658586, SRR7658642, SRR7658670 and SRR7658672)^13^ and one from Tanzania (SRR1930179)^57^ were excluded because none of the reads aligned to the gene catalogue. A Wilcoxon rank-sum test with Bonferroni correction was performed for each of the genes. To ensure that genes enriched in the palaeofaeces were not merely soil contamination, we excluded genes enriched in the soil samples compared to the present-day samples from the list of genes enriched in the palaeofaeces (Supplementary Table 8).

### CAZy analysis

To predict CAZymes^28^ from PROKKA protein output files (.faa files), hmmsearch (v.3.1b2)^101^ was run against dbCAN HMMs v8^102^ and an *e*-value cut-off of less than 1 × 10^−5^ was used^102^. Five Fijian samples (SRS475540, SRS475681, SRS476013, SRS476143 and SRS476277)^31^, one HMP sample (SRS018313)^103^ and one Spanish sample (V1.UC59.4)^56^ were excluded because they had no predicted CAZyme. CAZyme relative abundances were calculated by dividing the number of times each CAZy family was predicted in each sample by the total number of CAZymes predicted in the sample. A two-tailed Wilcoxon rank-sum test with FDR correction was performed for each CAZy family. To identify CAZy families that were enriched in the soil samples relative to present-day samples, a one-tailed Wilcoxon rank-sum test with FDR correction was performed for each CAZy family. These soil-enriched CAZy families were removed from the list of CAZy families. Statistically significant CAZy families were manually annotated with broad substrate categories.

### Jaccard distance matrix

To calculate pairwise Jaccard distances, binary matrices were used as inputs. For Extended Data Fig. 5a, a species binary matrix was created from MetaPhlAn2 output. To do this, MetaPhlAn2 output files were collapsed into a relative abundance matrix with the columns as samples and the rows as species. A binary matrix was created by recording non-zero cells as 1. For Extended Data Fig. 5b, a binary matrix was created with the columns as samples and the rows as genes. The presence of a gene in a sample was recorded as 1. Pairwise Jaccard distance was calculated using the Python package scikit-bio (http://scikit-bio.org/), specifically using the pw_distances function from skbio.diversity.beta package. The result was visualized as a heat map.

### Analysis of short versus long DNA fragments

To check whether the long DNA fragments found in the palaeofaeces were from contamination with modern DNA, we divided each sample into two subgroups: a subset containing only the long reads (>145 bp) and a subset of only the short reads (≤145 bp), and compared the species and gene composition among those subsamples. For Extended Data Fig. 5a, species were identified by MetaPhlAn2^20^, and the resulting binary species matrix was used to calculate pairwise Jaccard distances. For Extended Data Fig. 5b, genes were identified by PROKKA (v.1.14.6)^38^. The outputs were used to build a binary matrix to calculate the pairwise Jaccard distances.

### Cloud computing

Analyses were conducted on Amazon Web Services spot instances using Aether^104^ and on the O2 High Performance Compute Cluster, supported by the Research Computing Group, at Harvard Medical School (http://rc.hms.harvard.edu).

### Statistics and reproducibility

Statistical significance was verified through Welch’s *t*-test, Fisher’s test or Wilcoxon rank-sum test as described. Multiple-hypothesis testing corrections were performed using either the FDR or the Bonferroni approach. Most of the statistical analysis and data visualization were performed in R using the packages tidyverse, ggplot2, purrr, tibble, dplyr, tidyr, stringr, readr, forcats, scales, grid, reshape2, Rtsne, ggfortify, factoextra, ggpubr, ggforce, ggrepel, RColorBrewer and pheatmap. Data analysis and visualization for *M. smithii* tip dating were performed using the Python libraries pandas, NumPy and Matplotlib. Simulation of the effects of aDNA damage on assembly was performed using the Python package SciPy. Throughout the Article, data presented as box plots are defined as follows: middle line, median; lower hinge, first quartile; upper hinge, third quartile; the upper whisker extends from the hinge to the largest value no further than 1.5× the interquartile range from the hinge; the lower whisker extends from the hinge to the smallest value at most 1.5× the interquartile range from the hinge; data beyond the end of the whiskers are individually plotted outlying points.

For Extended Data Fig. 1c, the analyses for Zape1, Zape2 and Zape3 were part of a large review of samples from this site. Ten other samples were presented independently^105^. An additional 50 samples were reviewed^106^. Thus, these images were part of an extensive study of 63 samples from the site. Thirty hours of scanning electron microscopy beam time were involved in making the images. The UT30.3 images were taken as part of an ongoing analysis of 98 samples from the Colorado Plateau. A total of 110 h of scanning electron microscopy beam time have been applied to characterizing the dietary components.

### Reporting summary

Further information on research design is available in the Nature Research Reporting Summary linked to this paper.

## Online content

Any methods, additional references, Nature Research reporting summaries, source data, extended data, supplementary information, acknowledgements, peer review information; details of author contributions and competing interests; and statements of data and code availability are available at 10.1038/s41586-021-03532-0.

## Supplementary information

Supplementary Information This file includes an Ethics Statement, Supplementary Information sections 1-12, Supplementary Figures 1-3, Supplementary Table 11, and Supplementary References.

Reporting Summary

Supplementary Table 1 Details of samples. Related to Extended Data Fig. 1. This includes description of archaeological samples, including C14 dating, assembly statistics, proportion of contaminant DNA, mtDNA haplogroups, and CoproID output. The file also describes details of the present-day Mexican samples and publicly available datasets used in this study.

Supplementary Table 2 Dietary analysis and seasonality interpretation of the paleofeces. Related to Extended Data Fig. 1c.

Supplementary Table 3 MetaPhlAn2 output. Related to Fig. 1 and Extended Data Fig. 1h. This includes complete taxonomic abundance matrix, a species binary matrix, and statistical analysis results for species, families, and phyla found in the samples.

Supplementary Table 4 Details of parasites found in the archaeological samples. Related to Extended Data Fig. 3.

Supplementary Table 5 Long vs. short reads analysis. Related to Extended Data Fig. 5a-b. This includes a complete taxonomic abundance matrix and a species binary matrix as identified by MetaPhlAn2, as well as a PROKKA-annotated gene abundance matrix.

Supplementary Table 6 Details of the MAGs. Related to Fig. 2 and Extended Data Figs. 6, 7, and 8.

Supplementary Table 7 Model selection for tip dating. Related to Fig. 3.

Supplementary Table 8 Molecular function analysis. Related to Fig. 4. This includes lists of enriched PROKKA-annotated genes, CAZyme analysis results, and lists of genes surrounding transposases. This file also includes lists of taxa that contain glycan-degrading enzymes, antibiotic resistance genes, and chitin CAZymes.

Supplementary Table 9 Details of all samples used in SourceTracker2 analysis. Related to Extended Data Fig. 1e.

Supplementary Table 10 Comparison of assembly results and species composition between UDG-treated and non-UDG treated libraries. Related to Extended Data Fig. 5c-e.

Peer Review File

## Data Availability

Raw sequencing data has been uploaded to NCBI Sequence Read Archive (SRA) under BioProject accession number PRJNA561510.

## Code availability

Scripts used for data analysis are publicly accessible at https://github.com/kosticlab/ancient-microbiome-denovo. The code used to quantify the effect of ancient DNA damage on the assembled sequences is publicly accessible at https://github.com/alexhbnr/effect_aDNAdamage_denovoassembly.
