# Supplementary material for: Reconstruction of ancient microbial genomes from the human gut
Source: Nature. 2021 May 12;594(7862):234–9. doi: 10.1038/s41586-021-03532-0 (PMC8189908; doi:10.1038/s41586-021-03532-0)
Supplement: Supplementary file 1 — This file includes an Ethics Statement, Supplementary Information sections 1-12, Supplementary Figures 1-3, Supplementary Table 11, and Supplementary References. [file 41586_2021_3532_MOESM1_ESM.pdf]

nature portfolio

https://doi.org/10.1038/s41586-021-03532-0

Supplementary information

Reconstruction of ancient microbial genomes from the human gut

In the format provided by the authors and unedited

Reconstruction of ancient microbial genomes from the human gut

Marsha C. Wibowo[1,2], Zhen Yang[1,2,3], Maxime Borry[4], Alexander Hübner[4], Kun D. Huang[5,6], Braden T. Tierney[1,2,7], Samuel Zimmerman[1,2], Francisco Barajas-Olmos[8], Cecilia Contreras-Cubas[8], Humberto García-Ortiz[8], Angélica Martínez-Hernández[8], Jacob M. Luber[1,2,9], Philipp Kirstahler[10], Tre Blohm[11], Francis E. Smiley[12], Richard Arnold[13], Sonia A. Ballal[14], Sünje Johanna Pamp[10], Julia Russ[15], Frank Maixner[16], Omar Rota-Stabelli[6,17], Nicola Segata[5], Karl Reinhard[18], Lorena Orozco[8], Christina Warinner[4,19,20], Meradeth Snow[11], Steven LeBlanc[21] & Aleksandar D. Kostic[1,2]†

[1]Section on Pathophysiology and Molecular Pharmacology, Joslin Diabetes Center, Boston, MA, USA.

[2]Department of Microbiology, Harvard Medical School, Boston, MA, USA.

[3]Department of Combinatorics and Optimization, University of Waterloo, Waterloo, Ontario, Canada.

[4]Department of Archaeogenetics, Max Planck Institute for the Science of Human History, Jena, Germany.

[5]CIBIO Department, University of Trento, Trento, Italy.

[6]Research and Innovation Centre, Fondazione Edmund Mach, San Michele all'Adige, Italy.

[7]Department of Biomedical Informatics, Harvard Medical School, Boston, MA, USA.

[8]Immunogenomics and Metabolic Diseases Laboratory, Secretaría de Salud, Instituto Nacional de Medicina Genómica, Mexico City, Mexico.

[9]Center for Cancer Research, National Cancer Institute, National Institutes of Health, Bethesda, MD, USA.

[10]Research Group for Genomic Epidemiology, National Food Institute, Technical University of Denmark, Kongens Lyngby, Denmark.

[11]Department of Anthropology, University of Montana, Missoula, MT, USA.

[12]Department of Anthropology, Northern Arizona University, Flagstaff, AZ, USA.

[13]Pahrump Paiute Tribe and Consolidated Group of Tribes and Organizations, Pahrump, NV, USA.

[14]Department of Gastroenterology, Hepatology and Nutrition, Boston Children's Hospital, Boston, MA, USA.

[15]Morrison Microscopy Core Research Facility, Center for Biotechnology, University of Nebraska-Lincoln, Lincoln, NE, USA.

[16]Institute for Mummy Studies, EURAC Research, Bolzano, Italy.

[17]Center Agriculture Food Environment (C3A), University of Trento, Trento, Italy.

[18]School of Natural Resources, University of Nebraska-Lincoln, Lincoln, NE, USA.

[19]Department of Anthropology, Harvard University, Cambridge, MA, USA.

[20]Faculty of Biological Sciences, Friedrich-Schiller University, Jena, Germany.

[21]Peabody Museum of Archaeology and Ethnology, Harvard University, Cambridge, MA, USA.

†email: aleksandar.kostic@joslin.harvard.edu

Table of Contents

**Ethics Statement**	3
**SI section 1.** Overview of samples, study design, and authentication measures	6
**SI section 2.** Dietary analysis results	9
**SI section 3.** Complete description of species analysis	15
**SI section 4.** Percentage of reads aligned to MetaPhlAn2 database	16
**SI section 5.** aDNA damage levels in Firmicutes and Bacteroidetes genomes	17
**SI section 6.** Simulations to quantify the effect of aDNA damage on the assembled sequences	18
**SI section 7.** Phylogenetic trees for genera that were assigned to many ancient bins	21
**SI section 8.** *Methanobrevibacter smithii* divergence estimates	24
**SI section 9.** Transposases	29
**SI section 10.** Mapping of genes to taxa	32
**SI section 11.** Analysis of antibiotic-resistance genes	34
**SI section 12.** Uracil DNA glycosylase (UDG) treatment analysis	35
**Supplementary References**	37

Ethics Statement

Throughout this study, complexities emerged that extend beyond the scientific findings. We recognize there are Indigenous people who view the palaeofaeces as a cultural connection to their ancestors which should never be overlooked. The study introduced us to the importance of refining methods to ensure communication is conducted not only openly and respectfully, but also collaboratively with those interested in learning more about the study and its conclusions. This is particularly vital due to the long history of mistrust and lack of communication between Indigenous groups and researchers that often neglect Indigenous voices, history, and opinions on projects carried out worldwide, and particularly in the U.S. Southwest where the palaeofaeces originated. It is our hope that the consultation described will serve as a template for future research on human palaeofaeces, and that consultation with the descendants of study subjects will occur prior to commencing the study.

While palaeofaeces are not subject to the Native American Graves Protection and Repatriation Act (NAGPRA) or other regulations, we believe it is important to go beyond the mere the letter of the law to remain credible to living communities with strong cultural ties to the specimens when addressing relationships between science and Indigenous perceptions. We acknowledge consultation should have been initiated prior to the project, however the process was inadvertently overlooked, leading to immediate steps taken to initiate an interactive process.

The research and data presented are based on the scientific analysis of palaeofaecal material from the U.S. Southwest and Mexico. Indigenous populations from both countries with connections to the locations where the palaeofaecal materials originated provided important contributions and further context to the study. We express our profound appreciation for allowing us to expand our scientific knowledge and hope this article stimulates discussions for the future.

Relying on our collaborative commitment, we met with representatives from multiple communities to share the research findings. In the spirit of transparency, we acknowledge our inability to meet with every possible stakeholder community due to the COVID-19 pandemic and other extenuating circumstances. Nonetheless, we believe it is important to sustain our outreach which serves as a benchmark for others working with culturally sensitive non-skeletal or other biological resources to recognize the centrality that exists among Indigenous communities.

Interested parties can contact the authors for more insight about which communities participated, recognizing that this process in ongoing.

Mexico. The Mazahua samples included in this study belong to the MAIS (Metabolic Analyses in an Indigenous Sample) cohort. All individuals are self-identified Indigenous members of the Mazahua ethnic group, and their parents and grandparents also self-identified as Indigenous. All participants provided informed written consent, allowing their comparison with ancient remains or other modern populations. When necessary, informed consent was translated from Spanish into an appropriate Indigenous language, with some individuals signing with their fingerprint or mark. The study was designed in accordance with the Declaration of Helsinki and approved by the Research, Ethics, and Biosafety Human Committees of the Instituto Nacional de Medicina Genómica (INMEGEN) in Mexico City (protocol number 12/2018/I).

Since Indigenous people are considered a vulnerable population, recruitment was systematically completed with the approval and guidance of the Indigenous leader of the Mazahua community and support from the National Commission for the Development of Indigenous Communities of Mexico (CDI, from the Spanish "*Comisión Nacional para el Desarrollo de Pueblos Indígenas*"). During community presentations, we agreed to conduct ourselves in accordance with the values of equity,

respect, and mutual collaboration. Emphasis was placed upon maintaining the integrity of all samples and preventing inappropriate, commercialized, or unauthorized use. Therefore, data can only be used for scientific purposes.

Communication Protocol: Multiple meetings were organized by the CDI. Preliminary data was discussed with the Indigenous leader and Mazahua volunteers who participated in the study. During these meetings, we presented and explained the findings as they relate to community health and/or the population's history. We welcomed questions and considered and addressed perceived doubts by offering individual data to participants of the study upon written request.

SI section 1 - Overview of samples, study design, and authentication measures

We collected and evaluated a number of palaeofaeces from a number of sites. Some were clearly not human, had poor preservation, etc. Fifteen samples met our criteria and we extracted DNA from these samples for shotgun metagenomic sequencing using ancient DNA (aDNA) techniques (see Methods). We obtained a mean sequencing depth of 363 million reads per sample (read length = 150 bp), de novo assembled the raw reads into draft genomes, and annotated genes in the metagenomes (Extended Data Fig. 1a). We subsequently excluded four palaeofaeces with poor assembly results ($\leq$ 1 high-quality genomes and $\leq$ 5 medium-quality genomes reconstructed; Supplementary Table 1), removed samples UT2.12 and AW116 due to high archaeological soil contamination levels as identified by SourceTracker2[72] (Extended Data Fig. 1e), and removed sample AW113 because CoproID[71] inferred its host source as *Canis familiaris* based on both the microbiome composition and alignment to host DNA (Supplementary Table 1). The remaining eight samples came from three sites labeled Boomerang Shelter, Arid West Cave, and Zape.

The authenticity of these eight samples (UT30.3 and UT43.2 from Boomerang; Zape1, Zape2, and Zape3 from Zape; and AW107, AW108, and AW110A from Arid West Cave) was further validated. First, the ancient origin of the samples was confirmed by C14 dating that suggests the ages of the palaeofaeces are consistent with dates of the sites they are from and span the first ten centuries of the Common Era (CE) (Extended Data Fig. 1b, Supplementary Table 1). Moreover, consistent with aDNA characteristics, the reads contain C-to-T damage patterns on the ends of the reads for both microbial and human DNA (Extended Data Fig. 2). We also calculated the proportion of present-day human mtDNA contamination that could have been introduced by sample handling using contamMix (v.1.0-10)[64], and the results reveal low contamination levels of less than 10% for all of the samples (mean = 1.75%, s.d. = 2.96) (Supplementary Table 1).

Second, the human origin of the palaeofaeces was validated by (i) CoproID[71] (Supplementary Table 1); (ii) microscopic analysis of dietary remains in the palaeofaeces that confirms the presence of domesticated and cultivated plant taxa, including maize pollen grains, *Ustilago maydis* spores, and cactus cladodes (Extended Data Fig. 1c, Supplementary Table 2, Supplementary Information section 2); and (iii) human mtDNA haplogroup analysis using HaploGrep (v.2.1.21)[67], which assigns all palaeofaeces to a major Native American mtDNA lineage[109,110] (Supplementary Table 1). This haplogroup is found at high frequency among many populations in the Southwest region of North America[111,112], and has been found in ancient human remains from the Americas[6,113–115].

Third, we confirmed that the samples are faecal samples. Many of the species identified by MetaPhlAn2[20] are common gut microbes, including *[Eubacterium] rectale*, *Faecalibacterium prausnitzii*, *Roseburia hominis*, *Prevotella copri*, and *Treponema succinifaciens* (Supplementary Table 3). Principal component analysis (PCA) and SourceTracker2[72] results show that species composition of the palaeofaeces falls within the diversity of present-day non-industrial gut microbiomes (Fig. 1b) and differs from soil metagenomes (Extended Data Fig. 1d-e), pointing to minimal soil microbe contamination. Furthermore, comparison of the palaeofaeces metagenomic data with parasite genomes reveals that the predominant parasites are Blastocystis ST1, ST2, and ST3 (Extended Data Fig. 3, Supplementary Table 4). In contrast, Blastocystis is not detected in the soil samples. Our finding aligns with a previous study showing that ST1-4 are the Blastocystis subtypes most frequently found in the human gut microbiome, and that ST2 predominates in non-industrialized, while ST4 is mainly detected in industrialized humans[116].

We noticed that these final eight samples are very well preserved and have long average DNA fragment sizes (Extended Data Fig. 4) with an average mode length of 174 bp (s.d. = 30.15). To ensure that these long DNA fragments are not modern contamination, we divided each sample’s metagenomic dataset into

two subsets: a subset containing only the long reads (> 145 bp) and a subset of just the short reads (30-145 bp), and compared species and gene composition among those sub-samples. The results reveal that the subsets cluster by sample and not read length, supporting that the long reads contain similar species and genes as the short reads, and thus represent a single microbial community (Extended Data Fig. 5a-b, Supplementary Table 5).

As a comparison to the ancient gut microbiome, we analyzed 789 present-day stool samples from both industrial and non-industrial populations (Extended Data Fig. 1b, Supplementary Table 1). These present-day industrial samples encompass three countries, including 169 individuals from the USA (147 from the Human Microbiome Project (HMP)[55] and 22 from Obregon-Tito et al.)[4], 109 from Denmark[56], and 140 from Spain[56]. For the present-day non-industrial samples, we analyzed gut metagenomes from five countries, including publicly available data of 174 individuals from Fiji[31], 36 from Peru[4], 112 from Madagascar[13], and 27 from Tanzania[57]. Moreover, we collected and performed shotgun metagenomic sequencing on 22 stool samples from individuals living in a rural Mazahua farming community in central Mexico. They had not received antibiotic treatment in at least six months prior to sample collection, consume a traditional agricultural diet rich in maize and beans, and have remained semi-isolated from industrialization and globalization.

SI section 2 - Dietary analysis results (related to Extended Data Fig. 1c, Supplementary Table 2)

Boomerang Shelter Dietary Overview. Sample UT43.2 is composed of rarely eaten foods. Woody stem fragments in the macroscopic remains appear to be terminal stems or spines from a shrub. The large amount of *Sarcobatus* (greasewood) pollen signals ingestion of male flowers from this plant. Large clusters of pollen signal floral ingestion. It is likely that the wood was ingested with the flowers. *Sarcobatus* male and female flowers are on separate plants. Male flowers grow in small spikes on the ends of stems and resemble small pinecones. This finding is unique in Southwestern palaeofaeces analysis. Other macroscopic remains from UT43.2 consist of nut fragments, similar to pine, and various remains of prickly pear pads (cladodes). Traces of *Chenopodium* seeds are present. The non-pollen microfossils are consistent with prickly pear cladodes, especially the druse phytoliths and Cactaceae lignin rings. The edible maize smut fungus, *Ustilago maydis*, is represented by millions of spores in the pollen preparation. *U. maydis* fruiting bodies are a common food in Mexico where it is called huitlacoche. *Sarcobatus* pollen grains, some aggregated in clusters derived from flowers, were also observed. In addition, plant cuticles and traces of fiber were present.

Sample UT2.12 was composed mostly of fine fiber and crushed ricegrass, *Achnatherum hymenoides* (*Oryzopsis hymenoides*), caryopses. The harvest of ricegrass has a low caloric profitability of less than 400 calories per hour. However, it can be harvested in June before preferable plant resources are available[117]. The pollen counts show that polleniferous foods derived from *Cleome* (beeweed) and maize were eaten. Sections of maize anthers were present in this sample. Thus, clear evidence of maize pollen harvest is present. This sample also contained orthopteran exoskeleton, either grasshopper or cricket. Macro remains included fruit exocarp.

Sample UT30.3 was composed mostly of masticated prickly pear cladodes including macroscopic elements of fiber, cuticle and epidermis. Microscopic remains were also dominated by cactus residue.

2,850,000 cactus lignin rings and druses were documented per gram of sample. Traces of maize, fractured hackberry seed, an unknown seed fragment and bone were also observed.

In summary, the Boomerang Shelter palaeofaeces series is atypical for the region and time period but reflect a human diet. The data contrast with those from other studies[118–121]. Of the nine samples from the site that were examined, of which only three were included in this study, four stand out in their unique or rare components such as greasewood and ricegrass. Only three contained milled maize. In other studies, milled maize is the most common component of Ancestral Pueblo diet[122]. The Boomerang Shelter palaeofaeces are consistent with famine foods[118,119]. Famine foods such as these have been documented from periods of drought or seasonal harvest fluctuations[119,123]

Arid West Cave Dietary Overview. A total of eight samples from Arid West Cave were analyzed for food residue. Only five were included in this study. The combined macroscopic and microscopic analyses provide a good idea of diet. It is noteworthy that maize starch was found in seven of the samples. The starch was in pristine form and altered form. Altered grains exhibit increased fissures in the grains and small compression points on the grains consistent with milling. Six samples were maize based. One sample, AW113, contained only maize, milled to 1-2 mm. Starch grains were abundant. Over a million altered starch grains were evident with 215,000 *U. maydis* spores. Another sample, not used here, had two colors of milled maize, milled to less than 1.0mm. Fine fiber from an unknown source is also present. Altered and pristine starch make up the majority of the macroscopic remains and *Cleome* (beeweed) flowers or buds were included in this spectrum. *Cleome* pollen frequently occurs in Ancestral Pueblo palaeofaeces. As a spice, *Cleome* buds were the most common condiment used in Pueblo cuisine from 200 CE onwards[120]. Sample AW110A contained maize that was milled to less than 0.5mm. Crush bone was also present in the sample. The microscopic remains contained over four million altered starch grains and rabbit hair. *Cleome* pollen amounted to 575,000 grains per gram.

Another sample not used here had unmilled and milled maize with seeds from *Physalis* (groundcherry) fruit. *Physalis* is a common Pueblo fruit similar to tomatillos. Two samples were dominated by maize but had a variety of additional components, while still another was composed of unmilled maize, coarsely milled (1-2 mm) maize, *Lycium* seeds from wolfberry fruit, cactus cladode druse phytoliths, animal hair, altered maize starch and gelatinized starch from cooking. Over 100,000 *Sarcobatus* (greasewood) pollen grains were present in this sample. Two starch sources were milled together in another unused sample maize and *Amaranthus* (pigweed) seeds. Interestingly, trichomes from the mustard family were abundant in this sample and are consistent with *Descurainia*. This shows that mustard greens were part of this diet. Cactus cladode microscopic elements and an abundance of altered maize starch were present. Maize starch is the only evidence of cultivated food in AW107. The majority of the other remains are finely milled non-cultivated grass caryopses, chaff, and stems. No traces of maize were found in AW108. Masticated orthopteran insects and non-cultivated grass caryopses, chaff, and stems composed this sample.

In general, palaeofaeces from Arid West Cave represent a typical Pueblo maize-reliant diet. Also harvested from the corn plant were *U. maydis* fruiting bodies and tassels. Therefore, pollen and fungus were part of the diet. Mustard greens and fruits of *Physalis* and *Lycium* and wild grass grains supplemented the maize-based diet.

Zape Dietary Overview. La Cueva de los Muertos Chiquitos is located in the Rio Zape, municipality of Guanaceví, Durango Mexico. The site has been the focus of parasitological, demographic, and botanical analyses. The demographic analysis was based on dental casts made from impression left on expectorated agave fiber masses called quids. Quids were the most common class of remains recovered from trash deposits at the site. From 50 randomly selected quids, 49 casts from different individuals were recovered[106]. Parasitological analysis showed a high level of infection with human-specific and

zoonotic parasites[48,124]. Dietary analysis indicates that maize, agave, squash, and cultivated beans were mainstays[47,105,106]. Minor dietary components include juniper, prickly pear cladodes, squash seeds, tomatillos, fish, and rodents.

Three palaeofaeces used in this study were analyzed for dietary remains: Zape1, Zape2, and Zape3. Sample Zape1 is composed mostly of an agave fiber mass and maize pollen. Traces of milled maize, fractured nuts, and goosefoot seed are also present. The microscopic analysis shows only maize pollen, 220,689 grains per gram in clusters from anthers. This indicates that maize anthers and tassels were processed as food. The site well represents the association of agave and maize documented previously[106]. Sample Zape2's total weight of macro remains is 0.13 grams. Unprocessed weight was 0.15 grams. The majority of the material is composed of succulent leaves with serrated margins and polygonal epidermal cells. Fine spongy fiber makes up the secondary portion. A maize cob terminal end was identified. Ground maize and crushed nuts are present with traces of milled maize. The microscopic remains are composed of 105,832,910 *Ustilago maydis* spores per gram of sample. Huitlacoche (maize mushroom) is an indigenous food of the region. This is the most ancient evidence of Huitlacoche in Mexico. Finally, sample Zape3 is composed of milled goosefoot seed, dropseed, maize, and insects. Pollen, probably from pigweed, or a related species in the Amaranthaceae, is very abundant. The pristine condition and abundance of the pollen indicates a source with ingested greens including buds. Squash pollen is present and signals the ingestion of squash blossoms. In general, the remains from these palaeofaeces are consistent with indigenous foods that persist among the inhabitants of the region.

Contrasting diets for the different populations

The prehistoric palaeofaeces and present-day Mazahua faecal samples represent three different dietary traditions. The modern Mazahua diet is diverse. Mazahua are especially known for their use of edible mushrooms[125]. However, the majority of calories comes from maize and secondarily wheat. Beans make

up a relatively minor component of the dietary cultivated plants. Fruits are eaten seasonally and include wild cherry (*Prunus serotina*) and to a lesser degree wild blackberries (*Rubus liebmanii*) and Mexican hawthorn (*Crataegus mexicana*). Mustard (*Brassica campestris*) is the source of the most popular greens followed by pigweed (*Amaranthus hybridus*), goosefoot (*Chenopodium berlandieri*), and yellowcress (*Rorippa nasturtium-aquaticum*).

The desert-adapted prehistoric Zape diet was also diverse[105]. *Agave* hearts (caudices) and five varieties of maize were the main food sources[106]. However, the diversity of supplemental cultivated plants was impressive including twelve varieties of beans and three species of cultivated squash. Additional wild food plants included acorns, piñon pine nuts, black walnuts, sunflower achenes, fruits and greens of goosefoot and pigweed, ground cherry (*Physalis*), and prickly pear fruits[47]. Trace foods included purslanes (*Portulaca*), sumac (*Rhus*), juniper berries, and dropseed grass (*Sporobolus*). A detailed analysis of ten Zape palaeofaeces showed dietary similarities to modern Tarahumara people[105]. They shared milled maize, huitlacoche (fungal fruiting bodies of *Ustilago maydis*), beans, groundcherry, and fermented maize. Importantly, prickly pear pads (cladodes) were evident in two samples and *Agave* caudices in four samples[105]. For *Agave,* inulin-type fructans are the primary carbohydrate. *Agave* fructans have an impact on the gut bacterial community including bifidobacteria[126]. In the Chihuahua desert, hunter-gatherers had a dietary intake of about 135 g bifidogenic inulin-type fructans per day for the average adult male[127]. For Zape, the consumption of *Agave* inulin-type fructans would have been substantial, especially in times when cultivated foods were less available. Prickly pear (*Opuntia*) cladodes also affect the gut bacterial community[128]. Prickly pear mucilage and pectic-derived oligosaccharides were observed to increase bifidobacteria population by 23.8% and 25%, respectively. Therefore, for Zape two key desert succulent food sources would have had a bifidogenic effect on the gut microbiome.

The Boomerang Shelter and Arid West Cave samples represent early farmers who subsisted on foods derived from maize including whole grain, flour, pollen, and huitlacoche. When the data for all palaeofaeces are reviewed, it is clear that they were very dependent on wild foods as well, of which prickly pear was the most important. Other foods included greens from mustard, pigweed, goosefoot, buds from beeweed (*Cleome serrulata*), goosefoot seeds, and wild fruits. Puebloans on the Colorado Plateau were reliant on cladodes and caudices of desert succulents[121,129], especially during periods of cultivated food shortage[119]. Therefore, the effects of the bifidogenic compound noted for Zape would have been present for the UT and AW samples. Unlike the Zape and Mazahua diets, the diversity for the Boomerang Shelter and Arid West Cave samples was low.

SI section 3 - Complete description of species analysis (related to Fig. 1)

Species enriched in the industrial samples relative to both the palaeofaeces and the non-industrial samples include *Akkermansia muciniphila* (two-tailed Fisher's test with FDR correction, $P = 2.2x10^{-2}$ and $P = 9.8x10^{-30}$, respectively) and members of the *Alistipes* and *Bacteroides* genera, such as *Alistipes onderdonkii* ($P = 0.0004$ and $P = 5.5x10^{-90}$), *Alistipes putredinis* ($P = 0.005$ and $P = 6.1x10^{-52}$), *Alistipes finegoldii* ($P = 0.005$ and $P = 8.2x10^{-34}$), *Alistipes shahii* ($P = 0.01$ and $P = 1.9x10^{-11}$), *Bacteroides vulgatus* ($P = 0.0006$ and $P = 2.1x10^{-15}$)*, Bacteroides xylanisolvens* ($P = 0.005$ and $P = 5.6x10^{-14}$), *Bacteroides thetaiotaomicron* ($P = 0.01$ and $P = 3.5x10^{-20}$), *Bacteroides caccae* ($P = 0.014$ and $P = 3.3x10^{-7}$), and *Bacteroides massiliensis* ($P = 0.02$ and $P = 9.3x10^{-36}$) (Fig. 1c, Supplementary Table 3). The only species enriched in the non-industrial samples relative to the palaeofaeces is *Bifidobacterium adolescentis* ($P = 0.008$). Species that are significantly more abundant in the palaeofaeces compared to both the non-industrial and industrial samples include *Ruminococcus champanellensis* ($P = 0.0003$ and $P = 9.6x10^{-9}$) and members of the *Enterococcus* genus: *Enterococcus mundtii* ($P = 1.03x10^{-11}$ and $P = 1.4x10^{-13}$), *Enterococcus hirae* ($P = 7.7x10^{-6}$ and $P = 5.4x10^{-13}$), *Enterococcus faecium* ($P = 1.5x10^{-6}$ and $P = 1.5x10^{-7}$), and *Enterococcus faecalis* ($P = 0.02$ and $P = 0.001$). Other species are enriched in both the palaeofaeces and the non-industrial samples compared to the industrial samples, such as *Ruminococcus flavefaciens* ($P = 0.02$ and $P = 0.02$), *Ruminococcus callidus* ($P = 0.01$ and $P = 2.3x10^{-31}$), *Butyrivibrio crossotus* ($P = 7.7x10^{-5}$ and $P = 4.2x10^{-66}$), and the spirochaete *Treponema succinifaciens* ($P = 2.4x10^{-14}$ and $P = 1.1x10^{-117}$).

SI section 4 - Percentage of reads aligned to MetaPhlAn2 database (Extended Data Fig. 1f)

To calculate the percentage of metagenomic reads mapped to MetaPhlAn2 database, we divided the number of aligned reads per sample by the total number of reads per sample. As expected, the HMP samples have the highest percentage of reads aligned, followed by the Mexican samples, the Fijian samples, the palaeofaeces, and the soil samples (Extended Data Fig. 1f; one-tailed Wilcoxon rank-sum; palaeofaeces vs. Fijian, $P = 1.72\text{x}10^{-6}$; palaeofaeces vs. Mexican, $P = 1.71\text{x}10^{-7}$; palaeofaeces vs. HMP, $P = 1.01\text{x}10^{-6}$).

The percentage of reads aligned to MetaPhlAn2 database per sample is not high, but this was expected. MetaPhlAn2 database includes an average of 184 (± 45) marker genes for each bacterial species[20]. Since an average bacterial genome contains 5,000 genes[130,131], MetaPhlAn2 database would cover about 3.68% of a given bacterial genome. Considering that many microbial species (77%) are still unknown or have never been described before (as shown in Pasolli et al.[13]) and that MetaPhlAn2 does not have the 'unknown' species in the database, the ~3% alignment rate for HMP samples is expected. For non-industrial samples (Fijian and Mexican), we expect an even lower rate of alignment because they are composed of more 'unknown' species that are not present in the MetaPhlAn2 database. For soil samples, the alignment rate is ~10 times lower because there are likely ~10 times more species that are not in the database, since the diversity of soil microbiome is 10 times higher than that of the gut microbiome[132].

SI section 5 - aDNA damage levels in Firmicutes and Bacteroidetes genomes (Extended Data Fig. 1g)

We observed a significantly higher abundance of Firmicutes in the palaeofaeces relative to the present-day industrial samples (Fig. 1a). One possible explanation is if Bacteroidetes are preferentially degraded relative to Firmicutes. This has been tested before and cell wall morphology and structure have not been found to account for differences in DNA preservation in ancient microbiome samples such as dental calculus[36]. In that study, terminal cytosine damage rates and DNA fragment length were found to be independent of cell wall structure (Gram-positive, Gram-negative, and/or presence of an S-layer). However, to test for this possible effect in palaeofaeces, we calculated terminal C-to-T and G-to-A substitution rates for both our medium-quality and high-quality pre-filtered and filtered (contigs with less than 1% damage removed) ancient genomes. As shown in Extended Data Fig. 1g, terminal damage rates are significantly higher in Firmicutes compared to Bacteroidetes (two-tailed Wilcoxon rank-sum test). This suggests that the high abundance of Firmicutes in the palaeofaeces is not due to preferential decay of Bacteroidetes. Further, our samples are very well preserved and the damage levels are very low. DNA damage is primarily driven by hydrolytic reactions, and the low levels of damage are thus likely due to the extreme desiccation of the cave sediments. Taken together, we do not expect that decay would significantly shift the taxonomic composition of the palaeofaeces.

SI section 6 - Quantification of the effect of aDNA damage on the assembled sequences using simulations (Extended Data Fig. 9)

In order to quantify the effect of aDNA damage, i.e., the increased frequency of C-to-T substitutions at the terminal ends of DNA fragments, on the assembled contigs, we performed assemblies on simulated short-read sequencing data with known amounts of aDNA damage.

For the simulation of the short-read sequencing data, we first determined four variables that might influence the assembly results with respect to aDNA damage: the GC content of the assembled genome, the sequencing depth along the genome, the amount of observed aDNA damage on the DNA fragments, and the mean length of these DNA fragments. In order to simulate data that reflected the empirical data, we determined which values were observed for these variables in the palaeofaeces samples based on the 498 medium-quality and high-quality MAGs assembled in this study (Supplementary Table 6; Extended Data Fig. 9a). For our simulation experiment, we then chose three microbial taxa which GC content reflected the lower end (*M. smithii*, 31%), the median (*Tannerella forsythia*, 47%), and the upper end (*Actinomyces dentalis*, 72%) of the GC content distribution (Extended Data Fig. 9b). Next, we generated short-read sequencing data from these reference genomes using gargammel's *fragSim* (v.1.1.2)[107] with three different read length distributions (Extended Data Fig. 9b). We added different amounts of aDNA damage to the resulting short-read sequences using gargammel's *deamSim* so that we observed nine levels of damage ranging from 0% to 20%, which was quantified by the amount of observed C-to-T substitutions on the terminal base at the 5' end of the DNA fragments (Extended Data Fig. 9b). This step was run five times for each combination of reference genome and read length profile in order to have replicates resulting in a total of 46 sequence samples per reference genome and read length distribution (five replicates of nine damage levels plus one sample without damage). Finally, we down-sampled the sequencing data of each of these sequence samples to five coverage bins by randomly drawing a number

from the uniform distribution defining each bin (Extended Data Fig. 9b) and generated paired-end sequences using gargammel's *adptSim*.

We assembled the simulated sequencing data using MEGAHIT[76] with default settings after removing adapters using AdapterRemoval v.2[60] and removing low-quality sequences using fastp[133]. The resulting contigs were filtered for a minimal length of 2.5 kb following the processing of the contigs assembled from the empirical data. Retained contigs were pairwise-aligned to their respective reference genome using BLASTn (v.2.5.0)[134] and substitutions relative to the reference genome were determined using an in-house Python script. Summary statistics were calculated using QUAST (v.4.6.3)[135,136].

First, we evaluated the overall number of mismatches between the assembled contig sequence and the reference genome which was used for simulating the short-read data (Extended Data Fig. 9c). In order to take the amount of the reference genome that was successfully assembled and aligned back to itself into account, we normalized the number of mismatches per 1,000 bp of aligned contig. Due to the low overall number of mismatches, we focused on the 95% quantile of the mismatch rate distribution, i.e., 95% of the contigs had a lower mismatch rate than this value. For all combinations of reference genome, read length distribution, aDNA damage, and coverage, we observed at most 10 mismatches per 1 kb assembled sequence, which is equal to a percent identity of 99% and higher. There was no apparent correlation between the amount of simulated aDNA and the number of mismatches observed, suggesting that the small number of mismatches detected was likely driven by genome complexity.

To further assess the effect of aDNA damage on the assembled sequences, we compared the number of C-to-T substitutions to the average number of all other substitutions per 1,000 bp of assembled sequence by calculating the $\log_2$-ratio (Extended Data Fig. 9d). Since aDNA damage manifests itself by a higher frequency of C-to-T substitutions at the terminal bases of DNA fragments, we would expect to observe a

higher number of C-to-T substitutions compared to other substitutions with respect to the reference genome. Only for a single combination of sequencing parameters, sequencing data simulated from *M. smithii* with a short-read length profile, a coverage between 5 to 10-fold, and no added aDNA damage, we observed a $\log_2$-ratio of more than 1 (1.34) indicating that we observed twice as many C-to-T substitutions than the average of all other substitutions. For all other scenarios, the $\log_2$-ratio was < 0.75 and for 321 of the 450 simulated samples even < 0, highlighting that C-to-T substitutions were not more frequent than other substitutions in the assembled sequences.

SI section 7 - Phylogenetic trees for genera that were assigned to many ancient bins (related to Fig. 2)

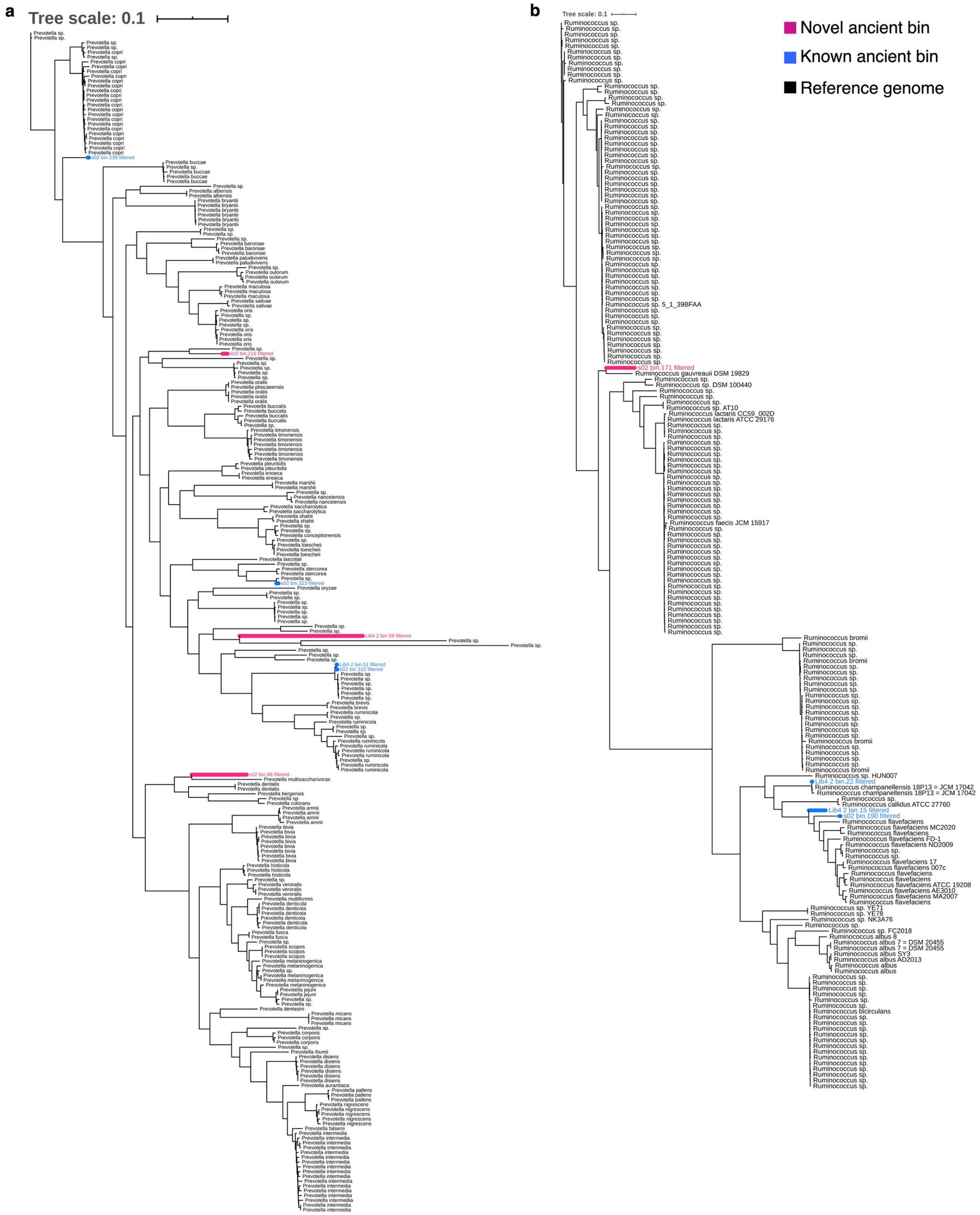

Supplementary Fig. 1 | *Prevotella* and *Ruminococcus* phylogenetic trees. These two genera were chosen because they were assigned to many ancient bins. Maximum likelihood tree was constructed

from multiple sequence alignments (MSA) of 120 bacterial marker genes as identified using the classify workflow in GTDB-Tk (v.0.3.0; default settings)[23].

a, *Prevotella* maximum likelihood phylogenetic tree. *Prevotella* genomes from NCBI RefSeq were used as reference genomes. Highly damaged filtered ancient genomes assigned to the genus *Prevotella* were included. Tree was manually pruned to reduce the number of leaves.

b, *Ruminococcus* phylogenetic tree. *Ruminococcus* genomes from NCBI RefSeq database were used as reference genomes. High-damage filtered ancient genomes assigned to the genus *Ruminococcus* were included.

Prevotella phylogenetic tree alignment inspection

To validate that the phylogenetic tree results were not due to misalignment, we visually inspected multiple sequence alignment files used to create the phylogenetic trees and confirmed that although the novel ancient SGBs were characterized by some highly divergent fragments, these divergences were always shared with at least one of the reference genomes (Supplementary Fig. 2). This indicates that the divergent fragments were genuine and not the result of misalignment or poor sequence quality.

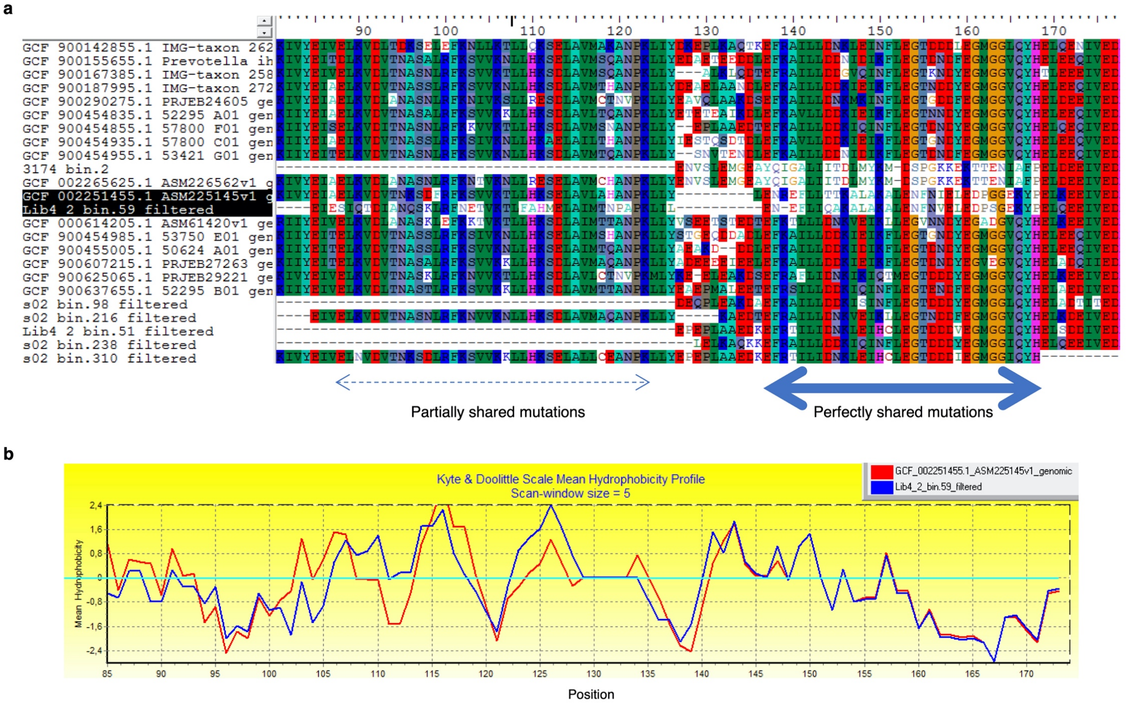

Supplementary Fig. 2 | Quality inspection of alignment for *Prevotella* phylogenetic tree.

a, A portion of the *Prevotella* alignment. Bold arrow depicts a typical example of divergent fragments in the ancient genomes that are almost perfectly shared with some of the reference genomes. In detail, Lib4_2_bin.59_filtered shares its mutations with GCF002251455. The only instance we could find of a divergent ancient fragment not clearly shared with the reference genomes is depicted by the dashed line: Lib4_2_bin.59_filtered shares only partial mutations (e.g., A94, K98, T104) with GCF000614205. However, the private mutations in Lib4_2_bin.59_filtered look genuine as they preserve the chemical properties of the fragment.

b, Amino acid hydrophobic profile of the fragments between positions 85 and 175 in Lib4_2_bin.59_filtered (blue) and the reference genome GCF002251455 (red). Profiles are very similar indicating a similar secondary structure. Images were generated using BioEdit[137].

SI section 8 – *M. smithii* divergence estimates (related to Fig. 3)

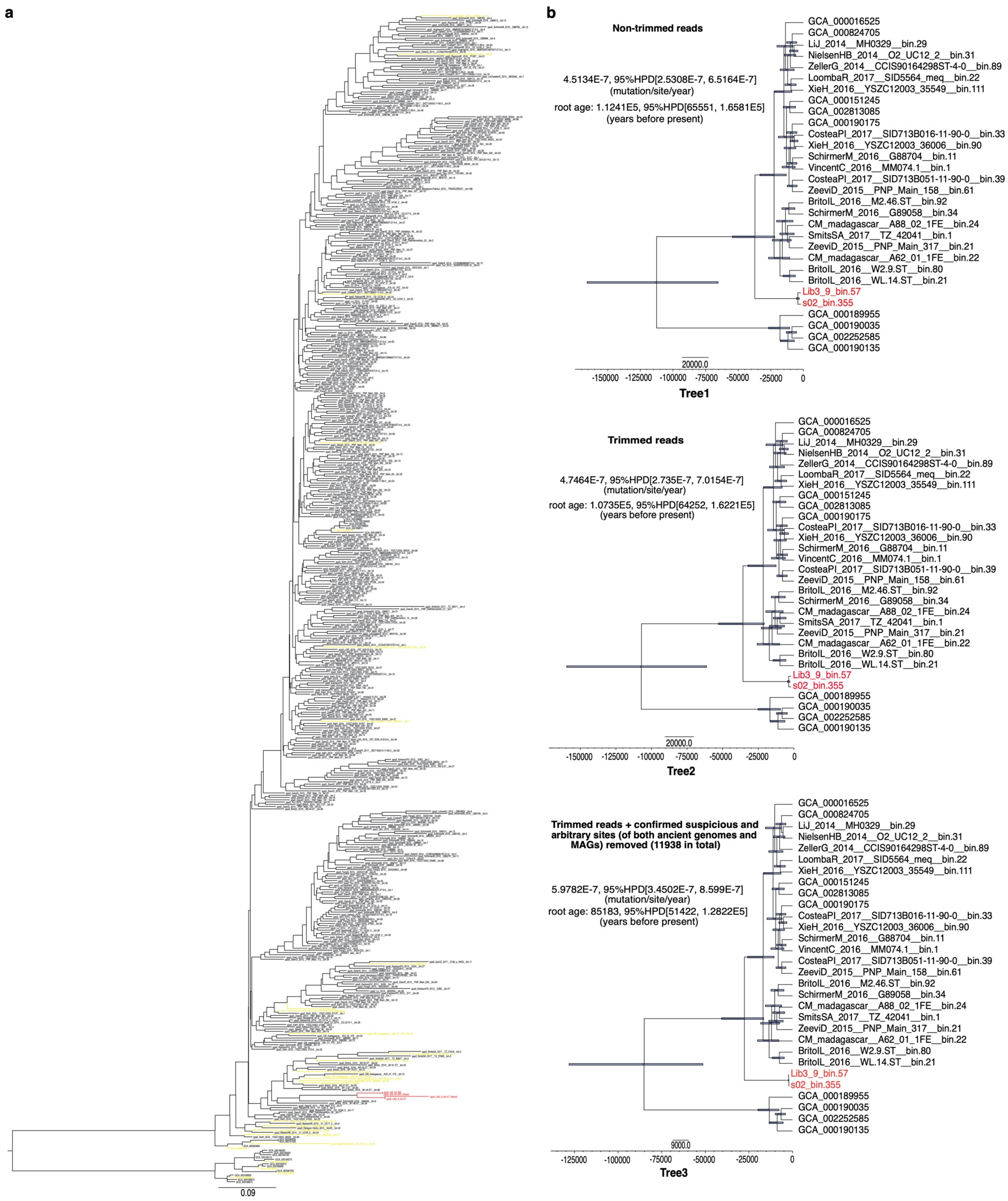

Supplementary Fig. 3 | *M. smithii* phylogeny and tip dating.

a, A whole phylogeny of *M. smithii*. Ancient *M. smithii* MAGs are highlighted in red. Contemporary *M. smithii* genomes included in the Bayesian phylogenetic analysis are displayed in yellow.

b, Comparison of tip dating results using three different versions of ancient *M. smithii* genomes: pre-filtered genomes (top panel), filtered genomes (middle panel; contigs with less than 1% damage were removed), and filtered genomes after trimming of the first and last 5 bp of the reads (bottom panel). The results are consistent among the three versions.

<u>Validation for *M. smithii* tip dating analysis using raw sequence divergence calculations (Extended Data Fig. 10)</u>

To validate our inferred dates in Fig. 3, we re-calibrated divergence dates between A & M1 *M. smithii* strains (Extended Data Fig. 10a) by calculating raw genetic distances. We divided all strains into three groups: M1(n = 24), A (n = 2), and M2 (n = 4) (Extended Data Fig. 10a). To measure raw sequence divergences between A & M2 strains, M1 & M2 strains, and A & M1 strains, we calculated single nucleotide variant (SNV) rates (the number of variant sites divided by the total number of sites considering only those not containing gaps, using the same reconstructed alignment used in our reported BEAST2 analysis) for each pair of strains and plotted the pairwise divergences for all group combinations (Extended Data Fig. 10b). The results show that sequence divergences (SNV per site) between M1 & M2 strains range from 0.0489 to 0.0546 with a mean of 0.0523; those between A & M1 strains range from 0.0187 to 0.0224 with a mean of 0.0205; those between A & M2 strains range from 0.0495 to 0.0514 with a mean of 0.0507.

To calculate systematic differences in substitutions per site per year, we used the formula d(M1, M2) - d(A, M2) ~ (substitutions per site per year * 1985 years), in which 1985 is the C14 mean corrected dates of samples UT30.3 and UT43.2 (Extended Data Fig. 1b, Supplementary Table 1). We started with a rough calibration by calculating d(M1, M2) - d(A, M2) using the average of d(M1, M2) and the average of d(A, M2): 0.0523 - 0.0507 = 0.0016. Based on the formula d(M1, M2) - d(A, M2) ~ (substitutions per site per year * 1985 yrs), we obtained substitutions/site/year = 0.0016/1985 = 8.060453e-07. Thus, the

divergence date between A and M1 based on the average sequence divergences can be obtained by: the average of d(A, M1) divided by 8.060453e-07 → 0.0205/8.060453e-07 = 25432.81 years (25.43 thousand years ago), which is quite close with the reported divergence date for A & M1 from our BEAST2 analysis (27.58 thousand years ago).

Further, we performed the analysis in more detail by using all pairwise sequence divergence values instead of just the averaged values. In detail:

First, we calculated all pairwise systematic differences between M1 & M2 (n = 96) and A & M2 (n = 8) strain divergences, resulting in 768 pairwise values of d(M1, M2) - d(A, M2) (Extended Data Fig. 10c). Those values range from -0.0025 to 0.0051, with a mean of 0.0016 (negative values indicate the few instances when M1 & M2 divergences are smaller than A & M2 divergences). As a comparison, we calculated systematic differences based on raw sequences (d(M1, M2) - d(A, M2)) with accumulated SNV rates (evolutionary rate * evolutionary time, which equates the product of inferred clock rate and the age of the common ancestor of two ancient strains) based on our BEAST2 analysis. This was performed by calculating the product of rate estimates from all iterations of simulation (Supplementary Table 7) and the inferred age of the common ancestor of the ancient strains (2198 BP, consensus tree file attached). The resulting values range from 0.0004 to 0.0025 with a mean of 0.0014 (Extended Data Fig. 10d). The distribution of results calculated based on the BEAST2 analysis falls well within the range of systematic differences calculated using raw sequence divergences, with a very similar mean estimate as well. These results indicate a preliminary consensus between the raw sequence divergence calculation and the inferred estimates from the BEAST2 analysis.

Second, we converted pairwise systematic differences (substitutions/site) to pairwise time-resolved systematic differences (substitutions/site/year), which could be used for calibrating the remaining

genetic distances, by dividing systematic differences with the average C14 age (1985 years old) of the two palaeofaeces (Extended Data Fig. 10e). In addition, we plotted the distribution of clock rate estimates (which are the signals in molecular clocking analysis) from the existing BEAST2 results (Extended Data Fig. 10f, Supplementary Table 7). As seen below, the distribution of our existing clock rate estimates is restricted in a narrow uncertainty range and falls within the range of time-resolved systematic differences based on raw sequence divergences (Extended Data Fig. 10e, f).

Third, we re-calibrated the genetic distances between A & M1 using the time-resolved systematic differences (d(A, M1)/time-resolved systematic differences, Extended Data Fig. 10g), which should give a result consistent with the estimated dates of the node leading to the divergence between A and M1 strains in Fig. 3. To compare the re-calibrated dates with our divergence date estimates, we re-plotted the distribution of all estimated A & M1 divergence dates from our BEAST2 analysis (Extended Data Fig. 10i, which has been shown as the orange violin plot in Fig. 3 as well). The re-calibrated dates based on systematic differences show a few low-frequency outliers (Extended Data Fig. 10g), which are likely noises that were ruled out after millions of iterations in BEAST2 simulation process. This is supported by the fact that our BEAST2 estimates with a narrow uncertainty range fall in the range of the re-calibrated dates based on raw sequence divergence (Extended Data Fig. 10g, i). The mean of our date estimates (27.58 thousand years ago) resembles that of the re-calibrated dates after low-frequency extreme values were removed (26.01 thousand years ago) (Extended Data Fig. 10h, i), which further strengthened the validity of our divergence date estimation.

Moreover, to test whether aDNA damage significantly affected the results, we repeated the same analysis described above using the common ancestor of the ancient strains. We inferred the common ancestor by removing all sites containing SNPs that are different between the two ancient strains. This is because aDNA damage should occur independently in the two ancient *M. smithii* genomes. We observed

and removed 40 such sites (alignment FASTA files and site positions attached). Based on the common ancestor of the two ancient strains, we obtained an average pairwise values of time-resolved systematic differences of 8.2679e-07 (substitutions/site/year) and an average of divergences of A & M1 strains of 0.0203 (substitutions/site). Thus, the re-calibrated date of divergence between A & M1 strains is 24.66 thousand years ago. The reported divergence dates are not significantly affected by aDNA damage. Instead, the dates shown in Fig. 3 largely reflect the process of mutation accumulation in the course of *M. smithii* genome evolution.

Taken together, the average divergence dates of A & M1 strains re-calibrated using raw sequence divergences, either for the original alignment or the alignment containing only the common ancestor of the ancient strains (26.01 and 24.66 thousand years ago, respectively), is largely consistent with the BEAST2 analysis estimates (27.58 thousand years ago, Fig. 3). However, compared to calculating raw sequence divergences, the BEAST2 analysis results show a higher confidence in estimation, supported by a narrow range of estimation uncertainty.

The inferred divergences indicate that *M. smithii* radiated in a timeframe compatible with the major human migrations. The origin of the lineage leading to the two ancient *M. smitihii* genomes are set between 40,000 and 16,000 years ago (mean = 27,000 years ago). These estimates predate (although there is a certain overlap toward the earlier 95% posterior estimates) the accepted age of human entry to North America via the Beringia bridge (20,000 - 16,000 years ago).

SI section 9 - Transposases (related to Fig. 4)

To compare functional genomic profiles of the palaeofaeces and the present-day samples, we annotated genes from the contigs of each metagenome with PROKKA[38] and built a non-redundant gene catalog with CD-HIT[100]. We aligned the raw reads from each sample to the gene catalog, calculated gene relative abundances, and performed one-tailed Wilcoxon rank-sum test with Bonferroni correction for each of the genes to identify genes enriched in the palaeofaeces or the present-day samples (Fig. 4a, Supplementary Table 8). To ensure genes enriched in the palaeofaeces are not merely soil contamination, we excluded genes enriched in the soil samples compared to the present-day samples from the list of genes enriched in the palaeofaeces. The top-15 most significantly enriched genes for each comparison are shown in Fig. 4a. We visualized the distribution of each gene with boxplots to confirm that the enriched genes are not driven by outliers (data not shown).

The results reveal that the ancient gut microbiome is enriched in transposases (Fig. 4a, Supplementary Table 8). Among the genes significantly more abundant in the palaeofaeces relative to the industrial samples, 37.5% are transposases. In comparison, merely 3.5% of the genes enriched in the industrial samples compared to the palaeofaeces are transposases (two-tailed Fisher's test, $P = 3.2 \times 10^{-9}$). To compare between the palaeofaeces and the non-industrial samples, since only eight genes are enriched in the non-industrial samples after Bonferroni correction, we selected genes with adjusted $P < 0.05$ after FDR correction. Among these, transposases account for 29.1% of the genes enriched in the palaeofaeces, but only 12.8% of those enriched in the non-industrial samples ($P = 3.2 \times 10^{-13}$). These transposases are also enriched in the non-industrial samples relative to the industrial samples (18.2% of the enriched non-industrial genes vs. 12.7% of the enriched industrial genes, $P = 3.0 \times 10^{-9}$).

To identify what genes frequently surround the transposases, for all of the samples, we identified contigs that contain 'transposase' or 'Transposase' from the PROKKA output files (.gff files). We took genes

that are from those same contigs and are within 1,000 bp (average gene length) from the transposases, and counted how many times each gene name appears per sample type (palaeofaeces or non-industrialized or industrialized). From the results, we removed genes with incomplete names (e.g., ‘n’, ‘in’, ‘ein’, ‘5’, ‘tein’, and ‘0’) and genes labeled as ‘hypothetical protein’ or ‘transposase’ (because the results consisted of an overwhelmingly large number of unique transposases). The top-50 genes surrounding transposases per sample type are shown in Supplementary Table 8 (Tab 5). We annotated the top genes with functions according to UniProtKB[108] and found that many of these genes are recombinases, DNA repair proteins, stress response proteins, and pyrimidine biosynthesis enzymes (Supplementary Table 11).

Supplementary Table 11 | Genes surrounding transposases that are within the top-50 for all three sample types (palaeofaeces, non-industrial, industrial).

Gene	Function (according to UniProtKB)
Low molecular weight protein-tyrosine-phosphatase YfkJ	Involved in ethanol stress resistance
Tyrosine recombinase XerC	Site-specific tyrosine recombinase
Thymidylate synthase	Pyrimidine biosynthesis
Thymidylate synthase 2	Pyrimidine biosynthesis
Tyrosine recombinase XerD	Site-specific tyrosine recombinase
putative protein	-
Thymidylate synthase 1	Pyrimidine biosynthesis
DNA-invertase hin	Serine recombinase family of DNA invertases
Imidazole glycerol phosphate synthase subunit HisH	L-histidine biosynthesis (amino acid biosynthesis)
Insertion sequence IS5376 putative ATP-binding protein	ATP binding
Very short patch repair protein	DNA repair
LexA repressor	DNA repair
DNA-binding protein HU	Histone-like DNA-binding protein to prevent DNA denaturation under extreme environmental conditions.
Endoribonuclease EndoA	Toxin - stress response
HTH-type transcriptional activator RhaR	Regulation of transcription
Orotate phosphoribosyltransferase	Pyrimidine biosynthesis
Sporulation initiation inhibitor protein Soj	Inhibits the initiation of sporulation
Accessory gene regulator protein B	Pathogenesis, quorum sensing

SI section 10 - Mapping of genes to taxa (related to Fig. 4)

To investigate which taxa in the samples carry the genes of interest, we checked for the presence of these genes in the MAGs. First, to map which MAGs possess the enriched genes, PROKKA (v.1.14.6)[38] was run for a total of 1,938 bins, which included all of the representative ancient bins, the representative Mexican bins, and the representative bins from Pasolli et al.[13] that originated from the industrial and non-industrial samples used here. A binary matrix was created with the bin names as columns and the genes as rows. Significantly enriched genes from the functional analysis were searched in the binary matrix. Second, to assign taxonomies to the bins, for the ancient and Mexican bins, the bins were annotated with the lowest taxonomic rank assigned by GTDB-Tk (v.0.3.0)[23]. For the bins from Pasolli et al.[13], taxonomic names were assigned based on the lowest taxonomic rank of their 'estimated taxonomy'. Finally, for each sample type (palaeofaeces or non-industrial or industrial), we plotted the number of bins within each taxonomic annotation that contain those specific genes (Supplementary Table 8, Tab 6). For instance, for the palaeofaeces, SusD-like protein P2 is found in two bins annotated as g__Prevotella. In the non-industrial samples, SusD-like protein P2 is found in 14 bins annotated as g__Prevotella.

The results show that glycan degradation genes (Endo-4-O-sulfatase and three SusD-like proteins) are mostly found in Bacteroidetes SGBs, including *Bacteroides* and *Prevotella* species (Supplementary Table 8). In the present-day samples, multiple tetracycline resistance genes are present in *Streptococcus mitis*, in line with previous findings showing that *S. mitis* develops resistance to tetracycline at a high rate[138,139], and in *Collinsella* SGBs, a genus that increases in abundance upon low dietary fiber intake[140].

Mapping of CAZy families to taxa

CAZy analysis was performed as described in the Methods section for all of the medium-quality and high-quality filtered ancient MAGs, the medium-quality and high-quality Mexican MAGs, and 1,451 representative MAGs from Pasolli et al.[13] that were reconstructed from the metagenomes used in our study. For each sample type (palaeofaeces or industrial or non-industrial), we plotted the number of MAGs within each taxonomic annotation that carry the chitin CAZymes (CBM14, CBM18, CBM19, CBM54, CE4, GH18, GH5_11, GH5_44, and GH8) (Supplementary Table 8, Tab 7).

SI section 11 – Analysis of antibiotic-resistance genes (Extended Data Fig. 11; related to Fig. 4)

To determine whether the antibiotic-resistance (AbR) genes are on plasmids or chromosomes, we first mapped which MAGs and contigs contain the AbR genes of interest. PROKKA (v.1.14.6)[38] was run for all of the medium-quality and high-quality filtered ancient MAGs, the medium-quality and high-quality Mexican MAGs, and 1,451 representative MAGs from Pasolli et al.[13] that originated from the metagenomes used in our study. Platon (v.1.5.0)[141] was run for the MAGs that possess those AbR genes to predict whether the contigs containing those AbR genes are on chromosomes or plasmids. According to Platon, none of these genes are on plasmids in the MAGs.

As shown in Fig. 4a, Supplementary Table 8, and Extended Data Fig. 11, there are additional AbR genes in the metagenomes that are not associated with MAGs. These AbR genes might be plasmid borne. Some of these AbR genes are prevalent in the present-day samples but are mostly absent in the palaeofaeces. These include most of the tetracycline resistance genes, extended-spectrum beta-lactamase PER-1, lincosamide resistance protein, and antibiotic efflux pump periplasmic linker protein ArpA. However, there are also antibiotic-resistance genes that are more abundant in the palaeofaeces compared to the present-day samples, such as fosfomycin resistance protein FosX and Daunorubicin/doxorubicin resistance ABC transporter permease protein DrrB (Supplementary Table 8, Extended Data Fig. 11).

SI section 12 - Analysis of Uracil DNA glycosylase (UDG)-treated libraries

aDNA damage patterns are characterized by the presence of uracil bases as a result of cytosine deamination[98,142,143]. With the long DNA fragments, low damage levels, and high sequencing depth of our samples, we expected that our results should be minimally affected by DNA damage. To examine whether DNA damage affected our assembly results, we repaired DNA damage by performing uracil DNA glycosylase (UDG) treatment, a method used to remove uracil from aDNA[51] (Methods). We removed these uracil residues using previously published protocol[51] and performed downstream analyses on these UDG-treated libraries.

The results from UDG-treated libraries were compared to the results for the non-UDG-treated libraries. UDG-treated libraries were sequenced at a much lower depth compared to their respective non-UDG-treated libraries, hence to make a fair comparison, each non-UDG-treated sample was downsampled using seqtk v1.0-1 (https://github.com/lh3/seqtk) to match the number of read pairs of its respective UDG-treated sample. Both UDG-treated samples and downsampled non-UDG-treated samples were processed through the same pipeline as described above (see Methods, Extended Data Fig. 1a). For species-level analysis, species were identified using MetaPhlAn2[20], pairwise Jaccard distances were calculated for all samples, and a *t*-Distributed stochastic neighbour embedding (*t*-SNE) analysis was performed. For gene-level analysis, each of the samples was run through the de novo assembly pipeline (MEGAHIT[76]), genes were predicted with PROKKA[38], a and non-redundant protein catalogue was generated using CD-HIT[100] with a 90% identity threshold using the following settings: -n 5 -c 0.9 -s 0.9 -aS 0.9. Pairwise Jaccard distances were then calculated. Assembly statistics and complete MetaPhlAn2 outputs are reported in Supplementary Table 10.

The results indicate that UDG-treated and non-UDG-treated libraries downsampled to same-sequencing depth contain similar species and gene composition (Extended Data Fig. 5c-e, Supplementary Table 10).

This establishes that DNA damage minimally influenced our results. However, the UDG repair protocol resulted in even shorter fragments of DNA (average mode length = 74 bp), which posed a challenge to our de novo assembly pipeline and resulted in lower assembly quality compared to the non-UDG-treated libraries (Supplementary Table 10). Since the species and gene composition of the non-UDG treated libraries reflect their respective UDG-treated libraries (Extended Data Fig. 5c-e), albeit at a higher quality, all data shown in this study are from the non-UDG-treated libraries.

Supplementary References

109. Achilli, A. *et al.* The phylogeny of the four pan-American MtDNA haplogroups: implications for evolutionary and disease studies. *PLoS One* **3**, e1764 (2008).

110. Tamm, E. *et al.* Beringian standstill and spread of Native American founders. *PLoS One* **2**, e829 (2007).

111. Lorenz, J. G. & Smith, D. G. Distribution of four founding mtDNA haplogroups among Native North Americans. *Am. J. Phys. Anthropol.* **101**, 307–323 (1996).

112. Snow, M., Durand, K., Gustafson, M. & Smith, D. G. Additional analysis of mtDNA from the Tommy and Mine Canyon sites. *Journal of Archaeological Science: Reports* **13**, 229–239 (2017).

113. Llamas, B. *et al.* Ancient mitochondrial DNA provides high-resolution time scale of the peopling of the Americas. *Sci Adv* **2**, e1501385 (2016).

114. Tackney, J. C. *et al.* Two contemporaneous mitogenomes from terminal Pleistocene burials in eastern Beringia. *Proc. Natl. Acad. Sci. U. S. A.* **112**, 13833–13838 (2015).

115. Fehren-Schmitz, L. *et al.* A Re-Appraisal of the Early Andean Human Remains from Lauricocha in Peru. *PLoS One* **10**, e0127141 (2015).

116. Beghini, F. *et al.* Large-scale comparative metagenomics of Blastocystis, a common member of the human gut microbiome. *ISME J.* **11**, 2848–2863 (2017).

117. Simms, S. R. Acquisition Cost and Nutritional Data on Great Basin Resources. *J. Calif. Gt. Basin Anthropol.* **7**, 117–126 (1985).

118. Minnis, P. E. Famine foods of the northern American desert borderlands in historical context. *J. Ethnobiol.* **11**, 231–257 (1991).

119. Sutton, M. Q. & Reinhard, K. J. Cluster analysis of the coprolites from Antelope House: Implications for Anasazi Diet and Cuisine. *Journal of Archeological Science* 741–750 (1995).

120. Reinhard, K. J., Edwards, S., Damon, T. R. & Meier, D. K. Pollen concentration analysis of Ancestral Pueblo dietary variation. *Palaeogeogr. Palaeoclimatol. Palaeoecol.* **237**, 92–109 (2006).

121. Reinhard, K. J. *et al.* Understanding the Pathoecological Relationship between Ancient Diet and Modern Diabetes through Coprolite Analysis: A Case Example from Antelope Cave, Mojave County, Arizona. *Curr. Anthropol.* **53**, 506–512 (2012).

122. Minnis, P. E. Prehistoric Diet in the Northern Southwest: Macroplant Remains from Four Corners Feces. *Am. Antiq.* **54**, 543–563 (1989).

123. Dominguez S, Reinhard KJ, Sandness KL, Edwards CA, Danielson D. The Dan Canyon Burial, 42A21339, a P III Burial in Glen Canyon National Recreation Area. *Lincoln: Midwest Archeological Center's Occasional Studies Series 26.* (1992).

124. Morrow, J. J. & Reinhard, K. J. The Paleoepidemiology of Enterobius vermicularis (Nemata: Oxyuridae) Among the Loma San Gabriel at La Cueva de los Muertos Chiquitos (600–800 CE), Rio Zape Valley, Durango, Mexico. *Comp. Parasitol.* **85**, 27–33 (2018).

125. FarfÁn, B., Casas, A., Ibarra-ManrÍquez, G. & PÉrez-NegrÓn, E. Mazahua Ethnobotany and Subsistence in the Monarch Butterfly Biosphere Reserve, Mexico. *Econ. Bot.* **61**, 173–191 (2007).

126. Ramnani, P., Costabile, A., Bustillo, A. G. R. & Gibson, G. R. A randomised, double- blind, cross-over study investigating the prebiotic effect of agave fructans in healthy human subjects. *J. Nutr. Sci.* **4**, e10 (2015).

127. Leach, J. D. & Sobolik, K. D. High dietary intake of prebiotic inulin-type fructans in the prehistoric Chihuahuan Desert. *Br. J. Nutr.* **103**, 1558–1561 (2010).

128. Guevara-Arauza, J. C., de Jesús Ornelas-Paz, J., Pimentel-González, D. J., Mendoza, S. R. & Luz María Teresita. Prebiotic effect of mucilage and pectic-derived oligosaccharides from nopal (Opuntia ficus-indica). *Food Sci. Biotechnol.* **21**, (2012).

129. Reinhard, K. J. & Danielson, D. R. Pervasiveness of phytoliths in prehistoric southwestern diet and implications for regional and temporal trends for dental microwear. *J. Archaeol. Sci.* **32**, 981–988 (2005).

130. Land, M. *et al.* Insights from 20 years of bacterial genome sequencing. *Funct. Integr. Genomics* **15**,

141–161 (2015).

131. Lane, N. & Martin, W. The energetics of genome complexity. *Nature* **467**, 929–934 (2010).

132. Blum, W. E. H., Zechmeister-Boltenstern, S. & Keiblinger, K. M. Does Soil Contribute to the Human Gut Microbiome? *Microorganisms* **7(9)**, 287 (2019).

133. Chen, S., Zhou, Y., Chen, Y. & Gu, J. fastp: an ultra-fast all-in-one FASTQ preprocessor. *Bioinformatics* **34**, i884–i890 (2018).

134. Altschul, S. F., Gish, W., Miller, W., Myers, E. W. & Lipman, D. J. Basic local alignment search tool. *J. Mol. Biol.* **215**, 403–410 (1990).

135. Gurevich, A., Saveliev, V., Vyahhi, N. & Tesler, G. QUAST: quality assessment tool for genome assemblies. *Bioinformatics* **29**, 1072–1075 (2013).

136. Mikheenko, A., Valin, G., Prjibelski, A., Saveliev, V. & Gurevich, A. Icarus: visualizer for de novo assembly evaluation. *Bioinformatics* **32**, 3321–3323 (2016).

137. Hall, T. A. BioEdit: a user-friendly biological sequence alignment editor and analysis program for Windows 95/98/NT. *Nucleic Acids Symposium Series* **41**, 95–98 (1999).

138. Teng, L. J., Hsueh, P. R., Chen, Y. C., Ho, S. W. & Luh, K. T. Antimicrobial susceptibility of viridans group streptococci in Taiwan with an emphasis on the high rates of resistance to penicillin and macrolides in Streptococcus oralis. *J. Antimicrob. Chemother.* **41**, 621–627 (1998).

139. Lancaster, H. *et al.* Prevalence and identification of tetracycline-resistant oral bacteria in children not receiving antibiotic therapy. *FEMS Microbiol. Lett.* **228**, 99–104 (2003).

140. Gomez-Arango, L. F. *et al.* Low dietary fiber intake increases Collinsella abundance in the gut microbiota of overweight and obese pregnant women. *Gut Microbes* **9**, 189–201 (2018).

141. Schwengers, O. *et al.* Platon: identification and characterization of bacterial plasmid contigs in short-read draft assemblies exploiting protein sequence-based replicon distribution scores. *Microb Genom* **6(10)**, mgen000398 (2020).

142. Hofreiter, M., Jaenicke, V., Serre, D., von Haeseler, A. & Pääbo, S. DNA sequences from multiple

amplifications reveal artifacts induced by cytosine deamination in ancient DNA. *Nucleic Acids Res.* **29**, 4793–4799 (2001).

143. Stiller, M. *et al.* Patterns of nucleotide misincorporations during enzymatic amplification and direct large-scale sequencing of ancient DNA. *Proc. Natl. Acad. Sci. U. S. A.* **103**, 13578–13584 (2006).
