# Supplementary material for: Reconstruction of ancient microbial genomes from the human gut
Source: Nature. 2021 May 12;594(7862):234–9. doi: 10.1038/s41586-021-03532-0 (PMC8189908; doi:10.1038/s41586-021-03532-0)
Supplement: Supplementary file 2 — Reporting Summary [file 41586_2021_3532_MOESM2_ESM.pdf]

nature research

Corresponding author(s): Aleksandar D. Kostic

Last updated by author(s): March 5, 2021

Reporting Summary

Nature Research wishes to improve the reproducibility of the work that we publish. This form provides structure for consistency and transparency in reporting. For further information on Nature Research policies, see Authors & Referees and the Editorial Policy Checklist.

Statistics

For all statistical analyses, confirm that the following items are present in the figure legend, table legend, main text, or Methods section.

n/a	Confirmed	
☐	☒	The exact sample size (*n*) for each experimental group/condition, given as a discrete number and unit of measurement
☐	☒	A statement on whether measurements were taken from distinct samples or whether the same sample was measured repeatedly
☐	☒	The statistical test(s) used AND whether they are one- or two-sided<br>*Only common tests should be described solely by name; describe more complex techniques in the Methods section.*
☒	☐	A description of all covariates tested
☐	☒	A description of any assumptions or corrections, such as tests of normality and adjustment for multiple comparisons
☐	☒	A full description of the statistical parameters including central tendency (e.g. means) or other basic estimates (e.g. regression coefficient) AND variation (e.g. standard deviation) or associated estimates of uncertainty (e.g. confidence intervals)
☐	☒	For null hypothesis testing, the test statistic (e.g. *F*, *t*, *r*) with confidence intervals, effect sizes, degrees of freedom and *P* value noted<br>*Give P values as exact values whenever suitable.*
☒	☐	For Bayesian analysis, information on the choice of priors and Markov chain Monte Carlo settings
☒	☐	For hierarchical and complex designs, identification of the appropriate level for tests and full reporting of outcomes
☒	☐	Estimates of effect sizes (e.g. Cohen's *d*, Pearson's *r*), indicating how they were calculated

Our web collection on statistics for biologists contains articles on many of the points above.

Software and code

Policy information about availability of computer code

Data collection	No software was used.
Data analysis	Analyses were conducted on Amazon Web Services (AWS) spot instances and on the O2 High Performance Compute Cluster, supported by the Research Computing Group, at Harvard Medical School (http://rc.hms.harvard.edu). Scripts utilized for data analysis are publicly accessible at https://github.com/kosticlab/ancient-microbiome-denovo. The code used to quantify the effect of ancient DNA damage on the assembled sequences is publicly accesible at https://github.com/alexhbnr/effect_aDNAdamage_denovoassembly. We used the following softwares for data analysis: AdapterRemoval v.2, Cutadapt v2.8, KneadData v0.6.1, Bowtie 2 v2.3.5.1, contamMix v1.0-10, mitoBench v1.6-beta, Picard MarkDuplicates v2.18.2, FreeBayes v1.1.0, HaploGrep v2.1.21, Galaxy (2019 build version), MetaPhlAn2 v2.7.5, the R package curatedMetagenomicData v1.16.0, SourceTracker2, CoproID v1.0, BBSuite v38.24, Kraken 2 v2.0.8-beta, MEGAHIT v1.2.9, BBMap v38.86, PROKKA v1.14.6, CD-HIT-EST v4.8.1, MetaBAT2 v2.12.1, CheckM v1.0.18, dRep v2.4.2, MUMmer v3.23, FastANI v1.3, Mash v2.1, GTDB-Tk v0.3.0, mapDamage2.0 v2.0.9, DamageProfiler v0.4.7, IQ-TREE v1.6.11, iTOL v5, SAMtools v1.9, PhyloPhlAn v3.0, Roary v3.13.0, RAxML v8.1.15, BEAST2 v2.5.1, Tracer v1.7, Tablet v1.19.09.03, hmmsearch v3.1b2, dbCAN HMMs v8, seqtk v1.0-1, gargammel v1.1.2, BLASTn v2.5.0, BioEdit v7.2, Platon v1.5.0, Python v2.7 and v3.5, R v3.5.2. Most of the statistical analysis and data visualization were performed in R using the packages tidyverse, ggplot2, purrr, tibble, dplyr, tidyr, stringr, readr, forcats, scales, grid, reshape2, Rtsne, ggfortify, factoextra, ggpubr, ggforce, ggrepel, RColorBrewer, and pheatmap. Data analysis and visualization for Methanobrevibacter smithii tip dating were performed using the Python libraries pandas, NumPy, and Matplotlib. Simulation of the effects of ancient DNA damage on assembly was performed using the Python package SciPy and summary statistics were calculated using QUAST v4.6.3. Pairwise Jaccard distance was calculated using the Python package scikit-bio.

For manuscripts utilizing custom algorithms or software that are central to the research but not yet described in published literature, software must be made available to editors/reviewers. We strongly encourage code deposition in a community repository (e.g. GitHub). See the Nature Research guidelines for submitting code & software for further information.

Data

Policy information about availability of data

All manuscripts must include a data availability statement. This statement should provide the following information, where applicable:

- Accession codes, unique identifiers, or web links for publicly available datasets
- A list of figures that have associated raw data
- A description of any restrictions on data availability

Raw sequencing data has been uploaded to NCBI-SRA in the form of the BioProject PRJNA561510.

Field-specific reporting

Please select the one below that is the best fit for your research. If you are not sure, read the appropriate sections before making your selection.

☒ Life sciences ☐ Behavioural & social sciences ☐ Ecological, evolutionary & environmental sciences

For a reference copy of the document with all sections, see nature.com/documents/nr-reporting-summary-flat.pdf

Life sciences study design

All studies must disclose on these points even when the disclosure is negative.

Sample size	No sample size calculation was performed. The sample size was limited by the number of participants we were able to recruit and collect samples from during a two-day visit to their village. These samples sizes were sufficient to observe statistically significant trends in microbiome composition.
Data exclusions	We excluded seven paleofeces from all analyses, but we have provided their MetaPhlAn2 taxonomic results in Supplementary Table 3 (Tab 2). In detail, these paleofeces were excluded due to poor assembly results (TS889, TS895, UT3.6, and TS929A), evidence of archaeological soil contamination (UT2.12 and AW116), or a non-human host source (AW113).
Replication	Reproducibility was verified by comparing our cohorts to these previously published cohorts: Human Microbiome Project Consortium (Nature, 2012), Li et al. (Nature Biotechnology, 2014), Obregon-Tito et al. (Nature Communications, 2015), Brito et al. (Nature, 2016), Pasolli et al. (Cell, 2019), Rampelli et al. (Current Biology, 2015). All attempts at replication were successful.
Randomization	Randomization is not relevant to our study as it is observational in nature.
Blinding	Metagenomic library construction, dietary analysis, and seasonality interpretation were performed blindly. Blinding is not applicable to the metagenomic analysis; all samples were analyzed computationally in a uniform manner.

Reporting for specific materials, systems and methods

We require information from authors about some types of materials, experimental systems and methods used in many studies. Here, indicate whether each material, system or method listed is relevant to your study. If you are not sure if a list item applies to your research, read the appropriate section before selecting a response.

Materials & experimental systems

n/a	Involved in the study
☒	☐ Antibodies
☒	☐ Eukaryotic cell lines
☐	☒ Palaeontology
☒	☐ Animals and other organisms
☐	☒ Human research participants
☒	☐ Clinical data

Methods

n/a	Involved in the study
☒	☐ ChIP-seq
☒	☐ Flow cytometry
☒	☐ MRI-based neuroimaging

Palaeontology

Specimen provenance	Paleofeces were collected from Boomerang Shelter, Arid West Cave, and La Cueva de los Muertos Chiquitos (Zape). Boomerang shelter lies in southeastern Utah and was excavated under Utah Antiquities Section, permit number U-01-NO. For the Arid West Cave site, a series of dry rock shelters were excavated, most likely from the Rasmussen Ranch Cave site in east central Utah, between the years 1920-1931 under an antiquities permit as discussed in previous publications (Morss et al., 1931; Morss et al., 1931; Spangler et al., 2018). For Zape, the cave was excavated in April 1957 and July 1960 by Richard Brooks, assisted by Sheilagh Brooks and Teodoro Corral. The work was supported by a grant from the Associates in Tropical Biogeography of the University of California. Permission to excavate was granted by the Mexican government through the Secre-taria de Educacion Publica. Ignacio Bernal and his colleagues in the Instituto Nacional de Antropologia e Historia assisted.

Specimen deposition	Paleofeces from Boomerang Shelter are curated at the Edge of the Cedars State Park Museum, Blanding, Utah, USA. Samples from Arid West Cave are curated at The Robert S. Peabody Institute of Archaeology, Andover, Massachusetts, USA. The collection from Zape is curated at the Anthropology Department of the University of Nevada, Las Vegas, USA.
Dating methods	The samples were submitted to DirectAMS for AMS C14 carbon dating measurement.

☒ Tick this box to confirm that the raw and calibrated dates are available in the paper or in Supplementary Information.

Human research participants

Policy information about studies involving human research participants

Population characteristics	We collected stool samples from 22 individuals (16 females and 6 males; age 43.4 ± 13.32 years, mean ± sd; BMI 30.8 ± 4.59, mean ± sd) from a Mazahua community in the center of Mexico.
Recruitment	Individuals were recruited to the study following IRB approval as well as permission from village elders. Recruitment was via word-of-mouth. Exclusion criteria were history of diabetes, medication use, and diarrhea within two weeks. There are no known biases that contributed to their inclusion into the study.
Ethics oversight	All study participants were recruited in accordance with a human subjects research protocol (IRB number: CEI 2018/01) approved by the Institutional Review Board of INMEGEN. Each participant provided a statement of informed consent, and we have complied with all of the relevant ethical regulations.

Note that full information on the approval of the study protocol must also be provided in the manuscript.
