# Supplementary material for: Reconstruction of ancient microbial genomes from the human gut
Source: Nature. 2021 May 12;594(7862):234–9. doi: 10.1038/s41586-021-03532-0 (PMC8189908; doi:10.1038/s41586-021-03532-0)
Supplement: Supplementary file 13 — Peer Review File [file 41586_2021_3532_MOESM3_ESM.pdf]

Peer Review File

Manuscript Title: Reconstruction of ancient microbial genomes from the human gut

Editorial Notes:

Reviewer Comments & Author Rebuttals

Reviewer Reports on the Initial Version:

Referee #1 (Remarks to the Author):

Review of some very old ....

Initially I was worried that this would yet again be one of those terrible coprolite studies filled to the brim with reagent and other kinds of contamination. Finding many "novel" species often is a tell-tale sign of bad things to come but I was pleasantly surprised that the "novel"
species are actually all from pretty normal gut microbiome genera (from a non-western gut microbiome perspective). This is perhaps an important point that needs to be mentioned specifically (the "novel" species are not "that" novel). Typically feces studies do not suffer (much) from contamination issues but coprolite studies do; in this study they
however did a very thorough job dealing with (most(?)) contaminants by looking at DNA damage, a very useful/important contamination recognition tool that should be implemented when looking at ancient DNA. Very importantly, they looked for DNA damage patterns in each of the reconstructed bacterial genomes individually. Indeed, a lot of contamination (insufficiently damaged DNA) was present.
Soil contamination analysis furthermore adds to the quality of the research (as yet another line of defense). The research does not claim to be perfect (L.177: "potentially a slight contamination of the paleofeces") but appears to do a proper job. This field needs a properly performed large study, with sensible methodology, findings and conclusions; for that reason I'm very positive about it.

It perhaps does try to oversell itself a little in regards to that much of what is missing/lost in western gut microbiomes can likely also be learned from comparison with non-coprolite sample collections (fresh samples from modern humans living in non-western environments), but it indeed opens up doors from a evolutionary history perspective.

Some general points:
Line 163: Phylum level analysis is just bad/lazy/terrible/archaic (pet peeve). At least make family level comparisons. This can easily be done and will be much more enlightening/interesting. The Spirochaetes phylum is indeed interesting on the phylum level for comparison reasons, but for Bacteroidetes you really need to down to the Bacteroidaceae/Prevotellaceae level. The same is true for Firmicutes (Lachnospiraceae/Ruminococcaceae/others).

Line 182: These HMP samples are from Americans. In (much of) western europe still plenty of people have many of these species,
apart from Treponema. Americans are probably simply "more lost" (from a gut microbiome perspective). Worth a thought?

Line 297: To mainly compare the coprolites with the HMP samples is perhaps a bit of a waste; of course there will be massive differences.
The question in my mind, from a evolutionary perspective, is how the strains found within coprolites compare with strains found in in the cohort of Mexican people in this study (as there might at least be a link between those). It is already known that the industrial human gut microbiome has diverged massively from non-

industrialized/rural/etc microbiomes. This study mainly shows very nicely that the "ancient microbiome" does indeed have much more in common (is actually quite similar) with people from non-industrialized "modern" humans. This is one of the main findings for me; previous studies typically have been of insufficient quality to clearly show this (I do not claim to have read every article in the field, but I can point to quite a few bad ones). The antibiotic resistance and virulence factor findings are however of course interesting (/logical, at least in regards to antibiotics). In any case, I would not mind seeing a few more focussed comparisons between the coprolites and the Mexicans.

Some comments in regards to the figures:

Figure 2.
2a: As stated earlier, at the very least go down to the family level, at least for Bacteroidaceae and Prevotella from the Bacteroidetes phylum and for Lachnospiraceae and Ruminococcaceae of the Firmicutes phylum.
It is fine if some of the groups are kept at the phylum level (or some other level) if somewhat rare/exotic for example, but for the main groups more more resolution should be provided in this stacked barchart figure.
Introduce an "other" category for all the very small not so relevant rare stuff. Those can still be shown in the source/supplemental data.

Figure 5.
5a: What part of this difference is simply caused by the overabundance of Bacteroides in HMP?
5bi: With which genera/species are these differences most strongly correlated with? (per pathway, perhaps name 1 or perhaps 2?)
5bii: As stated, HMP is perhaps not the best group to compare paleofeces with. We should compare paleofeces with various groups high in prevotella etc (the mexican samples), and then see if stuff is indeed lost/different. Otherwise, you might as well compare high-Prevotella/Spirochaetes human groups (or even non-captive primates)
with the HMP group to see what is lost in the HMP group; there would be no need at all for paleofeces. Am I missing something?

Extended Data Fig. 3/4: Where is the sequencing data of these dietary remains? Any eukaryotic information? I'm just seeing some Fungi in ab1-MetaPhlAn2_output and info on parasites. Were there not any plant remains sequenced? There is plenty of information on pollen; why not on DNA? Why not link these up?

Some requests:
1: Could you perhaps also clearly/separately show all the stuff that you threw away, and at which step? What was filtred out (per sample)?
What was the taxonomy of this? This would be highly interesting/educational. Perhaps make a supplemental file out of this?

2: Could you include AZ113 in the Tab1-MetaPhlAn2_output? It would be a waste not to show the dog data.

<u>Referee #2 (Remarks to the Author):</u>

Wibowo et al present an interesting study on metagenomic profiling and MAG reconstruction from 1000-2000 year old human coprolites. Metagenomic analysis of ancient DNA has mostly been focused on dental calculus and this study is amongst the first on faecal material to my knowledge. The data appear to be compelling suggesting that the authors have in fact managed to retrieve bona fide ancient genomes from the paleofeces, but the evidence could be improved if some form of tip dating could be performed (see comments below).

The taxonomic and functional insights are consistent with existing literature comparing modern day industrialised vs non-industrialised faecal microbiomes, and the identification of novel species found only in the coprolites is exciting. Is there any evidence that these novel species are present in non-human primate faecal

microbiomes or anywhere else in the environment? One missing element in the study is mapping over- and under-represented functions back to their taxonomic origins via the MAGs where possible (see comments below). This would help to cross validate taxonomic and functional observations.

Specific comments

Line 97. Although likely beyond the scope of this study, an ancient dog gut microbiome may also be of interest. Indeed, it could serve as a reference point for degree of change in this host species relative to human over the same time interval.

Line 159. What percentage of metagenomic reads mapped to reference genomes using MetaPhlAn2? Did the ancient samples have a lower read mapping than the modern samples as might be expected? These data are probably in the supplementary tables somewhere but I found these difficult to navigate as they're not even labelled by their supp table numbers. Regardless, it would be useful to the reader to summarise this high level information in the main text.

Line 165. To play devil's advocate, can you rule out preferential decay of Bacteroidetes DNA in the paleofeces? Perhaps Gram positive cells (Firmicutes) are preserved better over the millenia than Gram negative cells (Bacteroidetes).

Lines 223&243. Are the 62 most confident ancient genomes suitable for SNP analysis, or would you expect numerous miscalled bases? If yes, then the subset of 22 assigned to existing species could be used for a SNP-based comparative analysis with modern representatives which would provide further support for their authenticity.

Line 238. There is a specific reference for GTDB-Tk: https://www.ncbi.nlm.nih.gov/pubmed/31730192

Line 245. Are any of the 71 novel ancient species represented in non-human primates? ie. by primate gut microbiome read mapping to these set of genomes.

Line 260. Long phylogenetic branches are often a sign of misalignment and/or poor sequence quality, and not especially compelling to make the case that these are genomes from ancient bacteria. A SNP-based comparison to modern members of the same species or genera may be more convincing, and/or some type of tip dating analysis if possible https://en.wikipedia.org/wiki/Tip_dating

Line 265. How was the DESeq2 analysis normalised? By total number of reads used? By universal marker genes? This is important to mention (in the results) when doing a comparison of this nature.

Lines 277&302. More virulent and pathogenic implies more Proteobacteria in modern humans. Members of this phylum are well known for carrying virulence genes, but have you mapped these functions back to MAGs that carry them to confirm?

Line 289. Sus operons are predominantly if not exclusively found in members of the Bacteroidetes (which are overrepresented in modern humans therefore consistent with this observation). Have you confirmed this with any of the MAGs that you recovered?

Referee #3 (Remarks to the Author):

Wibowo et al. report shotgun metagenome sequencing on eight paleofeces samples gathered from two sites in the southwestern USA and one site from Mexico. The authors used several methods to infer that much of the resulting DNA sequencing data was of ancient human origin, including carbon dating, microscopy analysis of dietary components, DNA damage profiling, analysis of host DNA found in the paleofeces, and community

composition analysis. The authors heavily leverage MetaPhlAn2 to compare community composition between ancient human stool samples with modern human stool samples from American, Fijian and Mexican communities. Genome bins were also recovered from paleofeces metagenomes, revealing many novel species-level groups. The authors also performed a functional analysis on genes predicted from metagenome assemblies.

The methods used to generate these data appear to be robust, and the data generated represent an exciting contribution to the field. While many of the findings presented here are confirmatory for what has been inferred about ancestral human gut microbiomes based on study of traditional populations, the thorough analysis to ensure the human source and age of the samples makes the resulting data and analysis very valuable. However, the metagenome analysis was lacking in several key areas and significant revisions are necessary.

Read-mapping and relative abundance methods and data are missing
The authors included very little information regarding the methods and results of read-mapping analyses from mapping a sample's own reads on its own assembly. Details that need to be described for each paleofeces metagenome sample include:
-Minimum contig length included in the bowtie2 index for read-mapping (line 893)
-Percentage of assembled reads that were mapped onto these contigs
-Minimum contig size used for MetaBAT2 genome binning
-Number and percentage of contigs binned using MetaBAT2 that appear in MQ or HQ genome bins
-Number and percentage of base pairs from contigs binned using MetaBAT2 that appear in MQ or HQ genome bins
-Describe the method used to calculate the coverage depth of each genome bin (Table S4, Tab1-coprolite genomes)

These data will allow the authors to answer questions such as:
-How much of the sequenced data (in % reads or % sequenced bp) can be accounted for by MQ or HQ genomes binned in this study
-Are the novel species-level genomes a major or minor component (abundance-wise) of the paleofecal metagenomes?

Additionally, coverage depth information for each gene annotated by PROKKA should be incorporated into the analysis to calculate fold change between samples (see below).

The authors should comment on the issues that arise when using MetaPhlAn2 (whose reference genomes largely come from industrialized countries) to estimate presence/absence and relative abundance of taxa in paleofecal samples. Other mapping analyses could be used to determine what % of paleofecal metagenome reads can be accounted for by:
-Genomes in the MetaPhlAn2 database
-Genome bins from the combined Pasolli, Almeida, and Nayfach papers
-Novel MQ+HQ species-level bins from all paleofecal samples

It is also important to determine whether novel genome bins recovered in one paleofecal sample can be detected in other paleofecal samples from this study.

Calculating and reporting species novelty

The authors describe 45% of their medium and high quality bins as "novel" due to these bins having Mash distance >0.05 to MAGs in the Pasolli, et al. data set as well as the genomes present on NCBI. Using Mash distance as the lone metric for determining novelty is insufficient. Mash provides an estimate for ANI identity but is not exact. The strength of this estimate is in part determined by the "sketch size", which the authors do not provide. The strength of the estimate is also weakened when genomes being compared are of less than high quality.

For a more rigorous quantification of novelty, the authors should use Mash as a means of clustering species at 90% identity, and then calculate exact ANI distances using ANImf or fastANI to determine if genomes exceed the 95% similarity threshold (this can be done using dRep, which the authors have used elsewhere in the paper).

Finally, the authors should clarify that genomes <= 95% ANI from all others in a reference dataset are not "novel species", but rather "novel species-level groups distinct from those in the existing reference database". This is in line with how others have reported novelty based on MAGs (Pasolli, et al., 2019).

Functional analysis

The pipeline that the authors used for functional analyses involved 1) annotation of genes using PROKKA (based on the UniProtKB database), 2) (random?) rarefaction to 100,000 genes per sample, 3) determining the number of genes assembled from each sample with each functional annotation, 4) identifying the top 100 enriched genes in paleofeces HMP samples and annotating them with broad functional categories, and 5) using a 2-tailed Fischer's test to determine enrichment of functional categories. In addition to a lack of methods describing how this pipeline was carried out, this approach has several distinct weaknesses.

First, this analysis has no consideration of gene abundance. The most abundant gene in a sample has the same weight as the least abundant gene, and both have an equal probability of being eliminated from analysis entirely during rarefaction. The analysis should consider gene abundance as well as existence, especially since similar genes in different genomes can assemble together to make one representative gene (which would then only be counted once).

Second, it is not clear from the methods section how DESeq2 was run on the resulting table of gene counts, nor it is clear that DESeq2 is an appropriate tool in this circumstance. DESeq2 is designed for RNA-seq read counts, not counts of numbers of assembled gene categories (which are likely non-normally distributed and may violate some of the statistical assumptions underlying DESeq2). Further, DESeq2 is notorious for generating many false positives (see Paulson et al. 2013 Nature Methods, and many others since). If DESeq2 is used, then the p value cutoff should be more like 0.000001, and the abundances of significant genes checked with a boxplot (on the log scale) to visually check if they are real or driven by one or two outliers.

Finally, it is not clear that the final Fisher test being performed is the best way to address functional differences. Not only is it relient on subjective annotation, but it's unclear that the final readout of "Are more genes of this category in the top 50 most enriched HMP genes vs Paleofeces genes?" is the right question to ask.

One way of addressing these concerns would be to map reads from all samples to a gene catalog and use this to calculate gene differential abundance. This would consider gene relative abundance and make for a more appropriate use-case for DESeq2 (with the caveats listed above). This gene catalog could include all genes assembled in this study, all CAZymes, and other existing gene catalogs. Genes could be annotated using a DIAMOND / BLAST search against UniRef, or with Prokka as is performed now. See (Li, J., Jia, H., Cai, X. et al. An integrated catalog of reference genes in the human gut microbiome. Nat Biotechnol 32, 834–841 (2014). https://doi.org/10.1038/nbt.2942) for an example.

Additional Specific Points:
-The authors describe how DNA damage patterns can be identified by mapping reads to a reference genome. Can the authors comment on how this would impact analyses such as assembly, read-mapping, and the generation of phylogenetic trees? Is it possible that ANI distances are being overestimated because damaged bases are contributing significantly to contig consensus sequences? If so, possibly modeling damage on simulated metagenomic data derived in silico from sequenced genomes (where the gold standard is known) could be used to correct ANI calculations, or establish a revised threshold.
-Line 94: Please provide more detail regarding what constitutes a "poor assembly result". Also consider

providing metrics such as mean read length following preprocessing, assembly N50, # contigs bigger than the minimum size used for MetaBAT2 binning, # base pairs in contigs bigger than min size for MetaBAT2 binning, etc.
-Line 259: It is stated that novel ancient genomes have long phylogenetic branches, and this does appear to be supported in Fig 4. It would be nice if this could be quantified (e.g. using a metric like “added branch length”).
-The paper would benefit from a more comprehensive analysis of genes relevant to complex carbohydrate degradation (e.g., dbcan) since the authors discuss enrichment of genes relevant to carbohydrate binding and import (susC,susD) in Bacteroidetes but do not discuss differential abundance of other relevant genes or gene clusters. Previous papers comparing traditional to industrialized microbiota highlight that plant polysaccharide-related CAZymes are more abundant in traditional populations, while mucin-degrading CAZymes are enriched in industrialized. A more comprehensive analysis along these lines could test whether similar findings hold in ancient samples and thus contribute to the overall breadth and relevance of the findings.

Minor points
-Line 93: “Assembled” should read “binned”
-Line 214: Were the Nayfach et al. bins included in the 385,236 bins used for MASH comparison? The total number of bins indicates that it was, but that paper is not cited in that instance.
-Line 229: “Out of these 71 bins, 20 have low variance.” It is unclear what this means.
-Line 1142: should read “milled maize” rather than just “milled"
-Figure 1, sub-panel B: report confidence intervals of mean corrected dates
-Figure 3g: edit x axis to not include half values, can’t have 0.5 or 1.5
-Extended Data Fig. 10: There is a random letter “e” floating to the right of “Autoinducer-2 kinase"

Referee #4 (Remarks to the Author):

Wibowo et al. report a de novo assembly of the ancient gut microbiome from 8 human paleofeces 1-2 thousand years old. They report that these are more similar in microbial composition to the gut microbiome of non-industrialized humans than to of industrialized humans. If true this is very important and interesting given recent years research showing the importance of our gut microbiome relative to varies health conditions. However, this type of reseach face some specific challenges of which some, but not all, are convisingly addressed by the authors:

1. The sequences are endogenous and ancient: The damage pattern reported by the authors suggest its the case, although the length distribution is unusual for ancient DNA. The reads (<100bp) are unusually long. Still, I buy this argument given we know very little on DNA preservation in microbes under varies preservation conditions.

2. The sequences are from the human gut and not from the surrounding environment: This is a crucial part as feces will not only be contaminated by contemporary DNA but with DNA from environmental microbes occupying the faces shortly after its delivered. Thus you will find a mixture of ancient microbial sequences of environmental and gut origins. How can you separate these? The authors argues some are typical of gut but not of environment, but what do we know about the surrounding environment microbes at the site back in time (we have only poor understanding of present settings)- nothing. Furthermore, with short reads how do we know that a given sequence if from a given gut microbe and not from a closely related environmental microbe of the past - We don't.

The authors needs too convincingly show that they can address point 2. A number of peoples have considered doing what the authors do, but have abandoned the idea simply because there has been no way to convincingly address point 2. For the authors to make a strong claim they need to solve this problem.

Author Rebuttals to Initial Comments:

Response to Referees' Comments for Manuscript 2020-02-01803 "Reconstruction of ancient microbial genomes from the human gut"

We would like to thank the referees for their thorough feedback and helpful suggestions. We believe their comments have notably strengthened our manuscript. First, in the revised manuscript, we have expanded the set of modern-day gut microbiome samples we analyzed to 789 samples from eight different countries. Throughout the manuscript, we present more detailed comparisons among the paleofeces, the non-industrial samples, and the industrial samples. Second, we further authenticated our paleofeces by comparing them to additional archaeological sediment samples from different time periods. Third, for the reconstructed genomes, we have improved our species novelty analysis by calculating Average Nucleotide Identity and by including non-human primate gut microbes to our collection of reference genomes. We also performed simulations to evaluate the effects of ancient DNA damage on assembled contig sequences, and performed a tip dating analysis using our reconstructed ancient *Methanobrevibacter smithii* genomes. Fourth, for our functional analysis, we have modified our methodology to take into account gene relative abundances as suggested, performed a CAZyme analysis, and linked the enriched functions back to the MAGs they originate from. Lastly, we have addressed the reviewers' comments to modify specific phrases and to include additional relevant details in the text, Figures, and Supplementary Materials.

In the following sections, we present our specific responses to the reviewers' comments.

Referees' comments:

<u>Referee #1 (Remarks to the Author):</u>

Comment: Review of some very old ....

Initially I was worried that this would yet again be one of those terrible coprolite studies filled to the brim with reagent and other kinds of contamination. Finding many "novel" species often is a tell-tale sign of bad things to come but I was pleasantly surprised that the "novel"
species are actually all from pretty normal gut microbiome genera (from a non-western gut microbiome perspective). This is perhaps an important point that needs to be mentioned specifically (the "novel" species are not "that" novel).

Response: To classify the genomes as either "novel" or "known", we follow previously described

standard of clustering genomes with >95% average nucleotide identity (ANI) to the same species[51]. To clarify this and to emphasize that the novel species-level genome bins (SGBs) belong to normal gut microbiome genera, as suggested, we have added the following text:

- In the Introduction (lines 78-81): “Out of our 181 most confident gut microbial genomes with DNA damage patterns characteristic of ancient DNA (aDNA)[21,22], 61 are novel SGBs (≥5% distance with all of the reference genomes[51]). Many of these novel SGBs belong to the typical human gut microbiome genera, and seven are shared across multiple paleofeces.”
- In the Results (lines 254-256 and 267-269): “Genomes with >95% ANI to at least one reference genome were classified as “known” SGBs, and the rest were classified as “novel” SGBs[14]. This 5% distance metric follows the definition of a bacterial species[51]” and “These are typical human gut microbiome genera, which supports that the curated genomes, including the novel SGBs, are gut microbes.”
- In the Discussion (lines 397-399): “Many of these novel SGBs belong to the typical human gut microbiome genera, which supports that they are gut microbes.”

Comment: Typically feces studies do not suffer (much) from contamination issues but coprolite studies do; in this study they
however did a very thorough job dealing with (most(?)) contaminants by looking at DNA damage, a very useful/important contamination recognition tool that should be implemented when looking at ancient DNA. Very importantly, they looked for DNA damage patterns in each of the reconstructed bacterial genomes individually. Indeed, a lot of contamination (insufficiently damaged DNA) was present.
Soil contamination analysis furthermore adds to the quality of the research (as yet another line of defense). The research does not claim to be perfect (L.177: "potentially a slight contamination of the paleofeces") but appears to do a proper job. This field needs a properly performed large study, with sensible methodology, findings and conclusions; for that reason I'm very positive about it.
It perhaps does try to oversell itself a little in regards to that much of what is missing/lost in western gut microbiomes can likely also be learned from comparison with non-coprolite sample collections (fresh samples from modern humans living in non-western environments), but it indeed opens up doors from a evolutionary history perspective.
Response: We agree with the reviewer that it is important not to overemphasize that the genomes we reconstructed have gone extinct. They might be present in modern-day non-western individuals but yet to be cultivated or sequenced. To address this, we have deleted phrases such as “missing species” and “extinct species” and replaced them with “novel microbial species”. We think it is more accurate to refer to them as novel species rather than extinct or missing species. These changes include:

- In the Abstract, we deleted the sentence “To discover bacterial species that might be missing in

modern populations,...”

- In the Abstract, we modified “This work opens the door to interrogating the evolutionary history of the human gut microbiota by genome reconstruction from paleofeces and may lead to discovery of missing bacteria important for human health.” to “This work opens the door to discovering novel gut microbes from ancient microbiome samples and interrogating the evolutionary history of the human gut microbiota by genome reconstruction from paleofeces.” (lines 50-52)
- In the Introduction, we modified “Therefore, identifying the missing species and their beneficial functions may give insights into aspects of human-microbiome symbiosis that have been lost[8].” to “Therefore, studying our ancestral gut microbiome might give insights into aspects of host-microbiome symbioses that have become altered in the modern, industrialized world[9].” (lines 56-57)
- In the Introduction, we modified “This work offers unprecedented insights into our ancestral gut microbiome and potentially extinct ancient microbial species.” to “This work offers unprecedented insights into our ancestral gut microbiome.” (line 87)

Comment: Some general points:

Line 163: Phylum level analysis is just bad/lazy/terrible/archaic (pet peeve). At least make family level comparisons. This can easily be done and will be much more enlightening/interesting. The Spirochaetes phylum is indeed interesting on the phylum level for comparison reasons, but for Bacteroidetes you really need to down to the Bacteroidaceae/Prevotellaceae level. The same is true for Firmicutes (Lachnospiraceae/Ruminococcaceae/others).

Response: We have now added phylum-, family-, and species-level results in Figure 2. Specifically, we included in:

- Figure 2a: Relative abundances of phyla that are significantly different between the paleofeces and the industrial samples or between the paleofeces and the non-industrial samples (one-tailed Wilcoxon test, FDR correction).
- Figure 2b: Relative abundances of “VANISH” (volatile and/or associated negatively with industrialized societies of humans) (Fragiadakis et al., Gut Microbes, 2019) and BloSSUM (bloom or selected in societies of urbanization/modernization) families (Sonnenburg & Sonnenburg, Science, 2019).
- Figure 2d: Heatmap of the presence/absence of species that are significantly different between the paleofeces and the industrial samples or between the paleofeces and the non-industrial samples (two-tailed Fisher’s test, FDR correction).

Comment: Line 182: These HMP samples are from Americans. In (much of) western europe still plenty of people have many of these species, apart from Treponema. Americans are probably simply "more lost" (from a gut microbiome perspective). Worth a thought?

Response: We thank the reviewer for this feedback. This has substantially expanded the scope of our study. As suggested, we have included additional samples from other regions to our taxonomic (Figure 2) and functional (Figure 5) analyses. Our modern-day samples now encompass 789 human gut metagenomes from eight countries, including:

- Modern-day industrial samples:
  - 147 individuals from the USA (Human Microbiome Project Consortium, Nature, 2012)
  - 249 individuals from Denmark and Spain (Li et al., Nat. Biotechnol., 2014)
  - 22 individuals from the USA (Obregon-Tito et al., Nat. Commun., 2015)
- Modern-day non-industrial samples:
  - 22 individuals from Mexico (this study)
  - 174 individuals from Fiji (Brito et al., Nature, 2016)
  - 36 individuals from Peru (Obregon-Tito et al., Nat. Commun., 2015)
  - 112 individuals from Madagascar (Pasolli et al., Cell, 2019)
  - 27 individuals from Tanzania (Rampelli et al., Curr. Biol., 2015)

These modern-day samples are described in the Results (lines 144-154) and the Methods section (lines 908-921).

Comment: Line 297: To mainly compare the coprolites with the HMP samples is perhaps a bit of a waste; of course there will be massive differences.

The question in my mind, from a evolutionary perspective, is how the strains found within coprolites compare with strains found in in the cohort of Mexican people in this study (as there might at least be a link between those). It is already known that the industrial human gut microbiome has diverged massively from non-industrialized/rural/etc microbiomes. This study mainly shows very nicely that the "ancient microbiome" does indeed have much more in common (is actually quite similar) with people from non-industrialized "modern" humans. This is one of the main findings for me; previous studies typically have been of insufficient quality to clearly show this (I do not claim to have read every article in the field, but I can point to quite a few bad ones). The antibiotic resistance and virulence factor findings are however of course interesting (/logical, at least in regards to antibiotics). In any case, I would not mind seeing a few more focussed comparisons between the coprolites and the Mexicans.

Response: To address this, instead of only comparing between the paleofeces and the industrial samples, we have now added comparisons between the paleofeces and five non-industrial societies.

As the reviewer pointed out, the findings suggest that the paleofeces are more similar to the modern-day non-industrial humans relative to the industrial humans both in terms of taxonomic composition (lines 156-205, Fig. 2) and gene composition (lines 314-370, Fig. 5). Not as many features are statistically significantly different between the paleofeces and the non-industrial samples compared to between the paleofeces and the industrial samples. We describe differences between the paleofeces and the non-industrial samples as follows:

- At the phylum level (lines 164-165): "None of the phyla is significantly different between the paleofeces and the non-industrial samples."
- At the family level (lines 187-188): "Compared to the non-industrial samples, only Spirochaetaceae is significantly higher in the paleofeces (p=0.004)."
- At the species level (lines 198-201): "The only species enriched in the non-industrial samples relative to the paleofeces is *Bifidobacterium adolescentis* (p=0.008). Meanwhile, species that are significantly more abundant in the paleofeces compared to both the non-industrial and industrial samples include *Ruminococcus champanellensis* (p=0.0003, 9.6×10^{-9}) and members of the *Enterococcus* genus."
- Genes enriched in the paleofeces (lines 333-334): "Among these, transposases account for 29.1% of the genes enriched in the paleofeces, but only 12.8% of those enriched in the non-industrial samples ($p=3.2 \times 10^{-13}$)."
- Genes enriched in the non-industrial samples (lines 338-339): "In comparison, both the industrial and the non-industrial samples are enriched in antibiotic resistance genes compared to the paleofeces (Supplementary Table 8)."
- CAZymes (lines 363-367): "The results reveal a similar enrichment pattern in the paleofeces and in the non-industrial samples compared to the industrial samples (Fig. 5c). For instance, starch- and glycogen-degrading CAZy families are enriched in the paleofeces and the non-industrial samples, while mucin- and alginate-related CAZy families are enriched in the industrial samples."

We did not compare the paleofeces with only the Mexican samples because even though those Mexican samples are from the same region as the paleofeces, the Mexican individuals do not preserve a very traditional lifestyle, but have only remained semi-isolated from the urban areas and represent a mixed traditional/industrial subsistence type. The taxonomic and functional compositions of these samples are not very reflective of the non-industrial gut microbiome. For instance, Fig. 2c shows that the Mexican samples are placed in between the industrial and the other non-industrial samples. Fig. 2d shows that even though the Mexican samples share some species with the other non-industrial samples (e.g. *Prevotella copri*, *Prevotella stercorea*, *Catenibacterium mitsuokai*), they lack *Treponema*

succinifaciens, a species present in most non-industrial samples but completely absent in the industrial samples. Moreover, Extended Data Fig. 8 shows that only one of the 195 species-level genome bins reconstructed from these Mexican samples is novel. At the gene level, the Mexican samples are not as abundant in transposases, but are more abundant in antibiotic resistance genes compared to many of the other non-industrial samples (Fig. 5a). Overall, we believe it is more comprehensive and informative to compare the paleofeces with all of the non-industrial samples from various regions rather than with only the Mexican samples.

Further, to address the question about how the strains in the paleofeces compare with modern-day microbial strains evolutionarily, we performed a tip-based dating for *Methanobrevibacter smithii.* We used two filtered (contigs <1% damage were removed) ancient *M. smithii* genomes from samples UT30.3 (1947 +/- 30 BP) and UT43.2 (1994 +/- 26 BP) as tip calibrations and selected 28 representative contemporary *M. smithii* genomes. The results indicate that using ancient genomes for calibrating *M. smithii* phylogenies, we could evolutionarily match previous studies on one of its sister species, *M. oralis* (Weyrich et al., Nature, 2017). This further supports the quality of our reconstructed genomes and the potential of utilizing ancient genomes reconstructed from paleofeces to study the evolutionary history of the gut symbionts. These results are described in lines 290-312 and shown in Fig. 4c and Extended Data Fig. 10, and the methodology is described on pages 54-55.

Comment: Some comments in regards to the figures:
Figure 2.
2a: As stated earlier, at the very least go down to the family level, at least for Bacteroidaceae and Prevotella from the Bacteroidetes phylum and for Lachnospiraceae and Ruminococcaceae of the Firmicutes phylum.
It is fine if some of the groups are kept at the phylum level (or some other level) if somewhat rare/exotic for example, but for the main groups more more resolution should be provided in this stacked barchart figure.
Introduce an "other" category for all the very small not so relevant rare stuff. Those can still be shown in the source/supplemental data.

Response: As described above, we have now added phylum-, family-, and species-level results in Figure 2. To show only the relevant taxa, for Fig. 2a and Fig. 2d, we only show phyla and species that are significantly different between the paleofeces and the industrial samples or between the paleofeces and the non-industrial samples. For Fig. 2b, we only include families that are members of the "VANISH" (volatile and/or associated negatively with industrialized societies of humans) (Fragiadakis et al., Gut

Microbes, 2019) and BloSSUM (bloom or selected in societies of urbanization/modernization) (Sonnenburg & Sonnenburg, Science, 2019) taxa. Moreover, we now visualize the results in boxplots (Fig. 2a-b) and a heatmap (Fig. 2d) instead of a stacked barchart to show the distribution of the relative abundances per sample more clearly. The complete results are shown in Supplementary Table 3.

Comment: Figure 5.

5a: What part of this difference is simply caused by the overabundance of Bacteroides in HMP?
Response: To address this, we have mapped the enriched genes back to the MAGs that carry them. In Fig. 5b and in the Supplementary Notes (page 100), we show that the enrichment of glycan degradation genes, including SusD genes, in the modern samples is due to the overabundance of *Bacteroides*. This is described in lines 354-358: "Moreover, multiple glycan degradation genes (Endo-4-O-sulfatase and three SusD-like proteins) are significantly enriched in the industrial samples compared to the paleofeces. We confirmed that these genes are mostly found in MAGs within the Bacteroidetes family (Fig. 5b), consistent with the higher abundance of Bacteroidetes in the industrial samples as detected by MetaPhlAn2 (Fig. 2) and with previous findings that Bacteroidetes species possess many glycan-degrading genes[64,65]."

However, the enrichment of antibiotic resistance genes in the modern samples is not due to *Bacteroides* overabundance, but rather those genes come from *Streptococcus mitis*, *Collinsela*, and some other taxa. This is shown on page 100 and described in the Results section (lines 349-352): "We found that in the modern samples, multiple tetracycline resistance genes are present in *Streptococcus mitis* SGBs, in line with previous findings showing that *S. mitis* develops resistance to tetracycline at a high rate[61,62], and in *Collinsella* SGBs, which increases in abundance upon low dietary fiber intake[63] (Supplementary Notes)."

For the transposases enriched in the paleofeces, there are many different transposase genes and they are present in various species/genera without a clear pattern. This is expected since many different microbes possess transposases. Therefore, in the manuscript we do not discuss which MAGs those transposases come from.

Comment: 5bi: With which genera/species are these differences most strongly correlated with? (per pathway, perhaps name 1 or perhaps 2?)
Response: As described above, we have now described these data in the Results section (lines 349-352 and 355-358) and included the plots in Fig. 5b and in the Supplementary Notes (page 100).

Comment: 5bii: As stated, HMP is perhaps not the best group to compare paleofeces with. We should compare paleofeces with various groups high in prevotella etc (the mexican samples), and then see if stuff is indeed lost/different. Otherwise, you might as well compare high-Prevotella/Spirochaetes human groups (or even non-captive primates) with the HMP group to see what is lost in the HMP group; there would be no need at all for paleofeces. Am I missing something?

Response: As described above, we have now included additional comparisons between the paleofeces and a panel of non-industrial populations at the taxonomic level (lines 156-205, Fig. 2) and the gene level (lines 314-370, Fig. 5).

Comment: Extended Data Fig. 3/4: Where is the sequencing data of these dietary remains? Any eukaryotic information? I'm just seeing some Fungi in ab1-MetaPhlAn2_output and info on parasites. Were there not any plant remains sequenced? There is plenty of information on pollen; why not on DNA? Why not link these up?

Response: We thank the referee for pointing this out. MetaPhlAn2 relies on clade-specific marker genes for ~13,500 prokaryotes, ~3,500 viruses, and only ~110 eukaryotes. Therefore, it is not designed to provide eukaryotic dietary information. As far as we know, there is no software and database that is specifically curated for identifying eukaryotic dietary remains.

To examine whether dietary information can be recovered from the sequencing data, we aligned the raw reads from each sample to genomic data available for plant species identified through analysis of dietary remains found from the microscopic analysis. We used Bowtie2 local alignment settings and used the representative genome of each species from NCBI as reference for the alignment. We included *Undaria pinnatifida*/seaweed as a negative control because it should not have been a part of the diet of the individuals whose paleofeces we analyzed. The percentages of reads aligned for each reference species are as follows:

SampleID	Zea mays (corn)	Chenopodium quinoa	Ustilago maydis	Cucurbita argyrosperma (squash)	Phaseolus vulgaris (beans)	Undaria pinnatifida (seaweed)	Cucurbita pepo (squash)
UT30.3	0.22	0.22	0.05	0.21	0.11	0.17	0.24
UT2.12	0.23	0.18	0.05	0.18	0.11	0.17	0.31
UT43.2	0.17	0.17	0.03	0.16	0.09	0.12	0.19
AZ107	0.36	0.25	0.04	0.26	0.11	0.2	0.32
AZ108	0.37	0.37	0.16	0.38	0.28	0.36	0.44
AZ110A	0.28	0.26	0.03	0.26	0.11	0.18	0.3
AZ113	0.32	0.31	0.07	0.32	0.17	0.25	0.37
AZ116	0.35	0.29	0.03	0.31	0.13	0.24	0.52
Zape1	0.24	0.17	0.02	0.18	0.06	0.13	0.24
Zape2	0.38	0.33	0.17	0.33	0.22	0.3	0.41
Zape3	0.31	0.29	0.08	0.25	0.15	0.22	0.3
1026.1.4	1.66	0.16	0.07	0.15	0.1	0.16	0.21
1043.4.1	0.73	0.11	0.05	0.11	0.06	0.13	0.12
3567.1.1	5.36	0.35	0.14	0.3	0.22	0.25	0.37

The highest alignment rate is between sample 3567.1.1 (soil) and *Zea mays*. Aside from that, the percentages of reads aligned across all species are quite low and similar to the amount aligned to the negative control (seaweed). This suggests that the low levels of alignment we observe in this analysis may be background noise from spurious alignment. Further research and optimization of eukaryotic sequence identification from metagenomic data is necessary to investigate the genetic preservation of dietary sequences in paleofeces. Such method development and analysis falls beyond the scope of this study, but will be a focus of future work.

Comment: Some requests:

1: Could you perhaps also clearly/separately show all the stuff that you threw away, and at which step? What was filtred out (per sample)?
What was the taxonomy of this? This would be highly interesting/educational. Perhaps make a supplemental file out of this?

Response: According to the reviewer's suggestion, we have now included in Supplementary Table 3 (Tab2-MetaPhlAn2_removed_samples) taxonomic composition of the samples we excluded from downstream analyses. The samples we excluded are described in the Results section (lines 95-100): "We excluded four paleofeces with poor assembly results (≤1 high-quality genomes and ≤5 medium-quality genomes reconstructed; Supplementary Table 1), removed samples UT2.12 and AZ116 due to high archaeological soil contamination levels as identified by SourceTracker2[23] (Extended Data Fig. 1), and removed sample AZ113 because CoproID[24] inferred its host source as *Canis familiaris* based on both the microbiome composition and alignment to host DNA (Supplementary Table 1)."

For each sample, our filtering steps are described in the Methods section (lines 936-939): “Adapters were removed from paired Illumina reads using AdapterRemoval v.2[94]. Human sequences were filtered out using KneadData (https://bitbucket.org/biobakery/kneaddata/wiki/Home) by mapping reads to *Homo sapiens* reference database (build hg19)[96]. For the archaeological samples, short reads of <30 bp were removed using Cutadapt[95]. ” The short reads of <30bp that we removed are too short to contain meaningful taxonomic information and were removed for quality control, so we did not analyze those with MetaPhlAn2. However, we have shown the taxonomic and gene composition of both a subset of the samples containing only the long reads (>145bp) and a subset containing only the short reads (30-145bp) in Extended Data Fig. 6 and Supplementary Table 5. These results are described in lines 135-142.

For the reconstructed genomes, as shown in Fig. 3a, we filtered out low-damaged contigs (<1% damage) from the genomes. This is described in lines 226-228: “We mapped reads to each contig, calculated the average damage across reads per contig, and removed contigs with average read damage <1% on either or both ends of the reads.” and in lines 1080-1082: “The 498 MQ and HQ assembled genomes were further curated by removing contigs with average read damage <1% at either or both ends of the reads.” The pre-filtered results, including the taxa those genomes belong to, are shown in Extended Data Fig. 7 and Supplementary Table 6 (Tab 1-paleofeces genomes and Tab2-paleofeces rep bins).

Comment: 2: Could you include AZ113 in the Tab1-MetaPhlAn2_output? It would be a waste not to show the dog data.
Response: We have now included sample AZ113 in Supplementary Table 3 (Tab2-MetaPhlAn2_removed_samples) together with the other samples that we excluded from downstream analyses.

Referee #2 (Remarks to the Author):

Comment: Wibowo et al present an interesting study on metagenomic profiling and MAG reconstruction from 1000-2000 year old human coprolites. Metagenomic analysis of ancient DNA has mostly been focused on dental calculus and this study is amongst the first on faecal material to my knowledge. The data appear to be compelling suggesting that the authors have in fact managed to retrieve bona fide ancient genomes from the paleofeces, but the evidence could be improved if some form of tip dating could be performed (see comments below).

Response: We have now performed a tip-based dating for *Methanobrevibacter smithii* using two filtered (contigs <1% damage were removed) ancient *M. smithii* genomes from samples UT30.3 (1947 +/- 30 BP) and UT43.2 (1994 +/- 26 BP) as tip calibrations. The results are described in lines 290-312 and shown in Fig. 4c and Extended Data Fig. 10 (also see response to reviewer comment below), and the methodology is described on pages 54-55.

Comment: The taxonomic and functional insights are consistent with existing literature comparing modern day industrialised vs non-industrialised faecal microbiomes, and the identification of novel species found only in the coprolites is exciting. Is there any evidence that these novel species are present in non-human primate faecal microbiomes or anywhere else in the environment?
Response: We have now added non-human primate gut microbial genomes to our collection of reference genomes. In our latest analysis, we calculated ANIs between the ancient genomes and reference genomes from various sources (described in lines 236-239 and 1060-1063). These reference genomes are not only from human gut microbiome samples, but also from other human body sites (154,723 MAGs from Pasolli et al., Cell, 2019), non-human associated bacteria and environmental bacteria (80,990 genomes from GenBank), and non-human primate gut metagenomes (2,985 MAGs from Manara et al., Genome Biol, 2019). Therefore, the ancient MAGs that are labeled as novel have not been previously characterized in the human gut, the non-human primate gut, or in environmental samples. They may still be extant today, but if so, they have not been identified or characterized to date.

Comment: One missing element in the study is mapping over- and under-represented functions back to their taxonomic origins via the MAGs where possible (see comments below). This would help to cross validate taxonomic and functional observations.
Response: We have now added this analysis, as described in the Results (lines 349-352, 355-358) and shown in Fig. 5b and in the Supplementary Notes (page 100). This is also further described in the response below.

Comment: Specific comments

Line 97. Although likely beyond the scope of this study, an ancient dog gut microbiome may also be of interest. Indeed, it could serve as a reference point for degree of change in this host species relative to human over the same time interval.
Response: We have now included the taxonomic composition of sample AZ113 (inferred as dog paleofeces by CoproID), as well as the other samples that we excluded from downstream analyses,

in Supplementary Table 3 (Tab2-MetaPhlAn2_removed_samples).

Comment: Line 159. What percentage of metagenomic reads mapped to reference genomes using MetaPhlAn2? Did the ancient samples have a lower read mapping than the modern samples as might be expected? These data are probably in the supplementary tables somewhere but I found these difficult to navigate as they're not even labelled by their supp table numbers. Regardless, it would be useful to the reader to summarise this high level information in the main text.

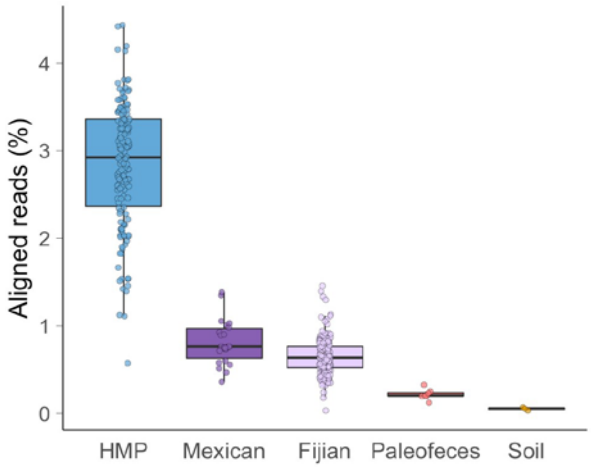

Response: We performed this analysis and described the results in the Supplementary Notes (lines 1519-1528): "To check for the percentage of metagenomic reads mapped to MetaPhlAn2 database, we divided the number of aligned reads per sample by the total number of reads per sample. As expected, the HMP samples have the highest percentage of reads aligned, followed by the Mexican samples, the Fijian samples, the paleofeces, and the soil samples (one-tailed Wilcoxon test, p-value paleofeces vs. Fijian=1.72×10^{-6}, p-value paleofeces vs. Mexican=1.71×10^{-7}, p-value paleofeces vs. HMP=1.01×10^{-6})." and in the Results (lines 161-162): "As expected, the paleofeces have a lower percentage of reads mapped to the reference database relative to the contemporary samples (Supplementary Notes)." The results are also shown in the following Figure.

We did not calculate the percentage of aligned reads for the other modern-day samples because we collected those MetaPhlAn2 output files from curatedMetagenomicData (Pasolli et al., Nat Methods, 2017), which only provides the taxonomic relative abundance files, but not the alignment results. However, we think this Figure shows clearly that, as expected, the paleofeces have a lower percentage of reads mapped to the MetaPhlAn2 database relative to the modern samples.

We apologize for the difficulty in navigating the Supplementary Tables. We have attempted to clarify these throughout the manuscript and added more details to our description of the Supplementary Tables (pages 103-105).

Comment: Line 165. To play devil's advocate, can you rule out preferential decay of Bacteroidetes DNA in the paleofeces? Perhaps Gram positive cells (Firmicutes) are preserved better over the millenia

than Gram negative cells (Bacteroidetes).

Response: This question has been tested before and cell wall morphology and structure has not been found to account for differences in DNA preservation in ancient microbiome samples such as dental calculus (Mann et al., Sci Rep, 2018). In that study, terminal cytosine damage rates and DNA fragment length were found to be independent of cell wall structure (Gram-positive, Gram-negative, and/or presence of an S-layer).

However, to test for this possible effect in paleofeces, we calculated terminal C->T and G->A substitution rates for both our pre-filtered and filtered (contigs with <1% damage removed) ancient genomes. As shown in the following Figure and in the Supplementary Notes (lines 1530-1553), terminal damage rates are higher in Firmicutes compared to Bacteroidetes. This suggests that the high abundance of Firmicutes in the paleofeces is not due to preferential decay of Bacteroidetes. Further, our samples are very well preserved and the damage levels in our samples are very low. DNA damage is primarily driven by hydrolytic reactions, and the low levels of damage are thus likely due to the extreme

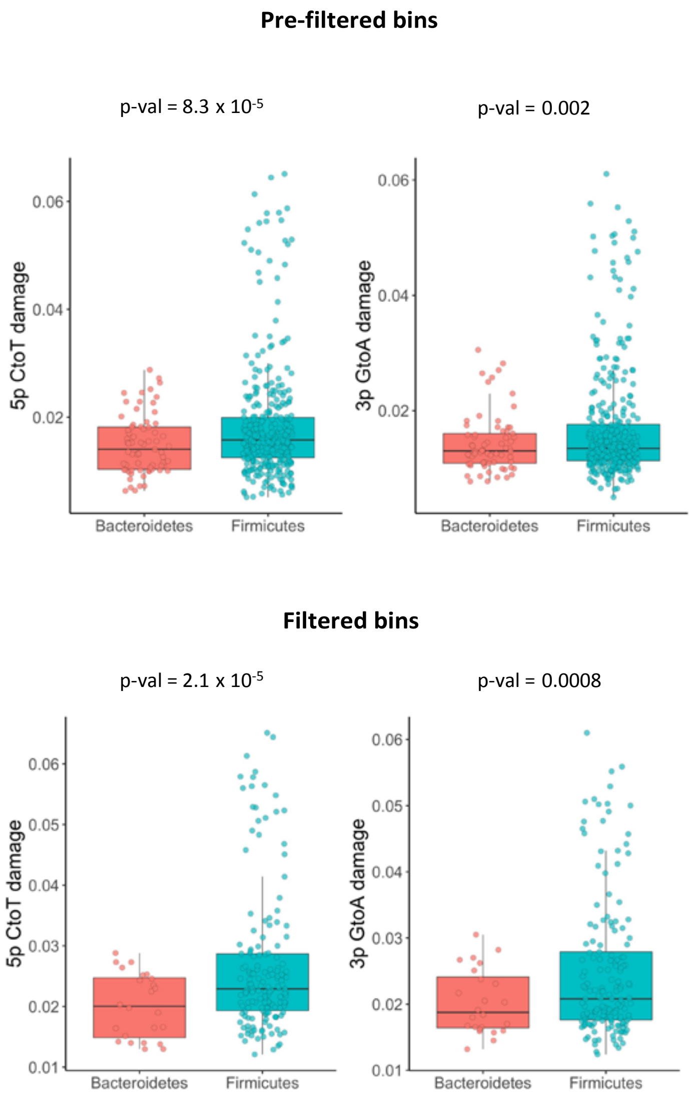

desiccation of the cave sediments. Taken together, we do not expect that decay would significantly shift the taxonomic composition of the paleofeces.

Comment: Lines 223&243. Are the 62 most confident ancient genomes suitable for SNP analysis, or would you expect numerous miscalled bases? If yes, then the subset of 22 assigned to existing species could be used for a SNP-based comparative analysis with modern representatives which would provide further support for their authenticity.

Response: The damage levels in our samples are very low and the coverage levels of our reconstructed genomes are high (mean=90x, sd=108). With such a deep coverage, we do not expect that aDNA damage would significantly be incorporated into the assembled consensus sequences. To quantitatively assess the effect of ancient DNA (aDNA) damage on the assembled genomes, we performed assemblies on simulated short-read sequencing data with known amounts of aDNA damage (lines 216-220 and pages 86-90). We varied the GC contents, aDNA damage levels, sequencing depths, and DNA fragment lengths of the simulated short-read data. The results show that there is minimal effect of aDNA damage on the number of mismatches between the assembled genomes and the original reference genomes. There is also no enrichment of C-to-T substitutions (typical aDNA damage pattern) compared to other substitutions in the assembled contig consensus sequences. Considering these results, we do not expect the reconstructed ancient genomes to have numerous miscalled bases from aDNA damage.

As the reviewer suggested, we performed a SNP analysis, described in the Supplementary Notes (pages 92-93). Among the 22 MAGs assigned to existing species, two MAGs are assigned to *Methanobrevibacter smithii*, while the rest of the assigned species only consist of one ancient MAG. Therefore, we performed a SNP analysis for *M. smithii*. The results show that there is a significant number of SNPs shared among the two ancient *M. smithii* genomes that are absent in the modern *M. smithi* genomes. This supports that the ancient *M. smithii* genomes are significantly more distant to the modern genomes relative to the modern genomes compared to each other.

Comment: Line 238. There is a specific reference for GTDB-Tk: https://www.ncbi.nlm.nih.gov/pubmed/31730192

Response: We thank the reviewer for pointing this out and have now incorporated this into the text.

Comment: Line 245. Are any of the 71 novel ancient species represented in non-human primates? ie. by primate gut microbiome read mapping to these set of genomes.

Response: To address this, we have now added 2,985 recently published MAGs from non-human primate gut metagenomes (Manara et al., Genome Biol, 2019) to our collection of reference genomes (described in lines 236-239 and 1060-1063). We calculated ANIs between the ancient genomes and those reference genomes. Therefore, the ancient MAGs that are labeled as novel have not been previously characterized in the non-human primate gut.

Comment: Line 260. Long phylogenetic branches are often a sign of misalignment and/or poor sequence quality, and not especially compelling to make the case that these are genomes from ancient bacteria. A SNP-based comparison to modern members of the same species or genera may be more convincing, and/or some type of tip dating analysis if possible https://en.wikipedia.org/wiki/Tip_dating
Response: We thank the reviewer for this suggestion. It has resulted in new analyses important for the manuscript, including SNP and tip dating analyses.

Regarding the length of the branches, the branch lengths of all novel ancient *Prevotella* bins in Fig. 4b, as well as those of ancient *Ruminococcus* and *Bacteroides* bins in Extended Data Fig. 9, are as long as (or even shorter than) the branches of their closer related (sister) reference genomes on the trees. Furthermore, the branches subtending the common ancestor of novel ancient bins and their sisters are in general quite long indicating genuine phylogenetic signals. These two pieces of evidence argue against poor sequence quality and/or misalignments in the ancient bins. To further validate that these results are not due to misalignment, we visually inspected the multiple sequence alignment files used to create the phylogenetic trees (see Figure below, also described on pages 90-91 in the manuscript). The results show that some novel ancient sequences are indeed characterized by some highly divergent fragments, but these divergences are always shared with at least one of the reference genomes. This indicates that the divergent fragments are genuine and not the results of misalignment or poor quality. We could find only one suspicious instance, at the very beginning of Lib4_2_bin.59 in the *Prevotella* alignment: mutations are only partially shared with a reference genome, but its private mutations seem genuine as they preserve the hydrophobic profile of the peptide.

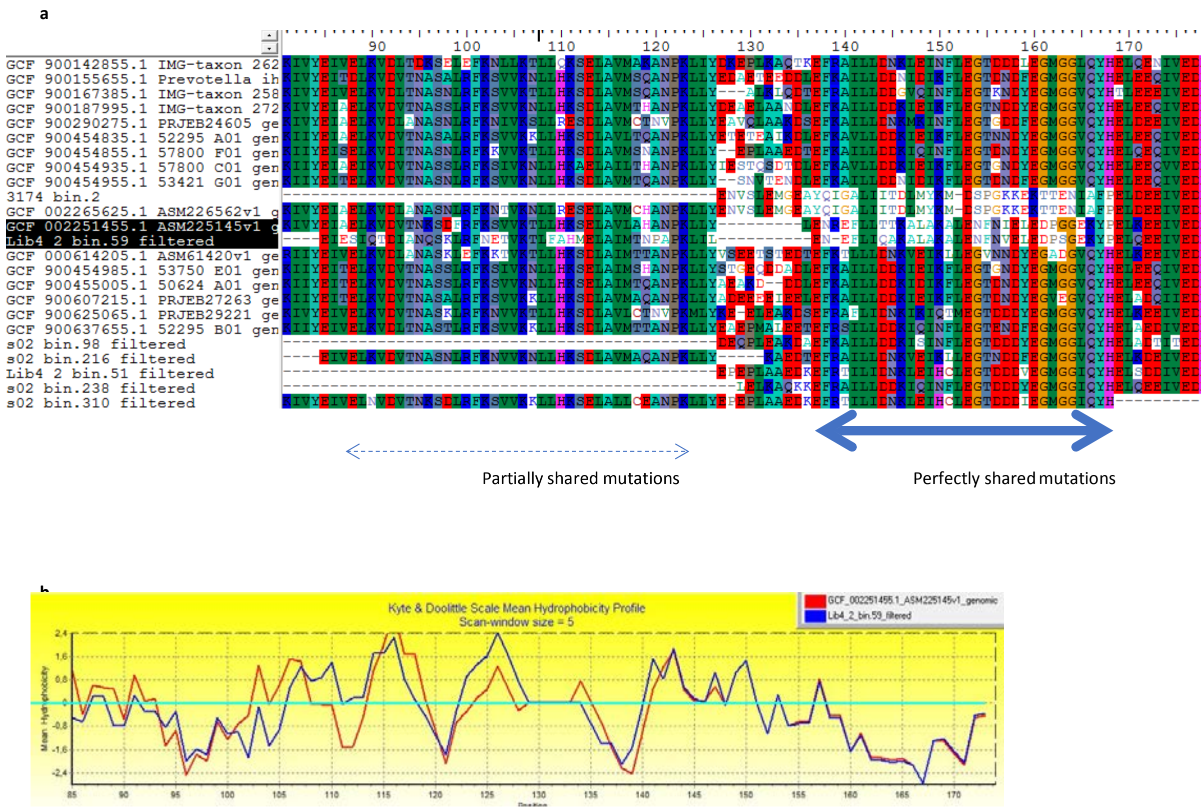

Quality inspection of alignment for *Prevotella* phylogenetic tree.

a, A portion of the *Prevotella* alignment. Bold arrow depicts a typical example of divergent fragments in the ancient genomes that are almost perfectly shared with some of the reference genomes. In detail, Lib4_2_bin.59_filtered shares its mutations with GCF002251455. The only instance we could find of a divergent ancient fragment not clearly shared with the reference genomes is depicted by the dashed line: Lib4_2_bin.59_filtered shares only partial mutations (eg: A94, K98, T104) with GCF000614205.

However, the private mutations in Lib4_2_bin.59_filtered look genuine as they preserve the chemical properties of the fragment. **b,** The amino acid hydrophobic profile of the fragments between position 85 to 175 in Lib4_2_bin.59_filtered (blue) and the reference genome GCF002251455 (red). Profiles are very similar indicating a similar secondary structure. Images have been generated using BioEdit.

However, we agree with the reviewer that we cannot exclude all possible technical issues that might have contributed to the long phylogenetic branches, so we have now modified the text to not overemphasize on whether the long phylogenetic branches suggest that the novel genomes are truly ancient. Specifically, we modified these previous sentences “For *Prevotella* (Fig. 4b), as well as the other genera (Fig. 4a, Extended Data Fig. 9), the novel ancient genomes have long phylogenetic branches. This supports our findings that these genomes are highly distant from the reference genomes.” to “Phylogenetic trees for genera that are assigned to many ancient bins (*Bacteroides*, *Prevotella*, and *Ruminococcus*) (Fig. 4b, Extended Data Fig. 9) show that the novel ancient genomes do not cluster closely with the reference genomes.” (lines 286-288)

Moreover, we performed a SNP analysis, as suggested by the referee (pages 92-93). Among the 22 MAGs assigned to existing species, two MAGs are assigned to *Methanobrevibacter smithii*, while the rest of the assigned species only consist of one ancient MAG. Therefore, we performed a SNP analysis

for *M. smithii*. The results show that there is a significant number of SNPs shared among the two ancient *M. smithii* genomes that are absent in the modern *M. smithi* genomes. This supports that the ancient *M. smithii* genomes are significantly more distant to the modern genomes relative to the modern genomes compared to each other.

We also performed a tip dating analysis for *M. smithii* using two filtered (contigs <1% damage were removed) ancient *M. smithii* genomes from samples UT30.3 (1947 +/- 30 BP) and UT43.2 (1994 +/- 26 BP) as tip calibrations. The results indicate that using ancient genomes for calibrating *M. smithii* phylogenies, we could evolutionarily match previous studies on one of its sister species, *M. oralis* (Weyrich et al., Nature, 2017). This further supports the quality of our reconstructed genomes and the potential of utilizing ancient genomes reconstructed from paleofeces to study the evolutionary history of the gut symbionts. These results are described in lines 290-312 and shown in Fig. 4c and Extended Data Fig. 10, and the methodology is described on pages 54-55.

Comment: Line 265. How was the DESeq2 analysis normalised? By total number of reads used? By universal marker genes? This is important to mention (in the results) when doing a comparison of this nature.
Response: The DESeq2 analysis was normalised by the total number of genes predicted by PROKKA per sample. However, to address comments from Referee #3 regarding how DESeq2 is not the best to use for this analysis, we re-performed the functional analysis using a different approach. We took into account gene relative abundances and performed Wilcoxon test instead of DESeq2 to identify differentially abundant pathways. The method is described in lines 1146-1160 and the results are described on pages 14-16 and Fig. 5 in the manuscript.

Comment: Lines 277&302. More virulent and pathogenic implies more Proteobacteria in modern humans. Members of this phylum are well known for carrying virulence genes, but have you mapped these functions back to MAGs that carry them to confirm?
Response: Since we modified our functional analysis methods, some of the results have

now changed. We now focus the analysis on glycan-degrading and antibiotic resistance genes (enriched in the modern-day samples) and transposases (enriched in the paleofeces). As suggested by the reviewer, we mapped these functions back to the MAGs that carry them. In Fig. 5b and in the Supplementary Notes (page 100), we show that glycan degradation genes, including SusD genes, mostly come from MAGs within Bacteroidetes. This is described in lines 354-358: "Moreover, multiple glycan degradation genes (Endo-4-O-sulfatase and three SusD-like proteins) are significantly enriched in the industrial samples compared to the paleofeces. We confirmed that these genes are mostly found in MAGs within the Bacteroidetes family (Fig. 5b), consistent with the higher abundance of Bacteroidetes in the industrial samples as detected by MetaPhlAn2 (Fig. 2) and with previous findings that Bacteroidetes species possess many glycan-degrading genes[64,65]."

On the other hand, antibiotic resistance genes come from *Streptococcus mitis*, *Collinsela*, and some other taxa. This is shown on page 100 and described in the Results section (lines 349-352): "We found that in the modern samples, multiple tetracycline resistance genes are present in *Streptococcus mitis* SGBs, in line with previous findings showing that *S. mitis* develops resistance to tetracycline at a high rate[61,62], and in *Collinsella* SGBs, which increases in abundance upon low dietary fiber intake[63] (Supplementary Notes)."

For the transposases, there are many different transposase genes and they are present in various species/genera without a clear pattern. This is expected since many different microbes possess transposases. Therefore, in the manuscript we do not discuss which MAGs those transposases come from.

Comment: Line 289. Sus operons are predominantly if not exclusively found in members of the Bacteroidetes (which are overrepresented in modern humans therefore consistent with this observation). Have you confirmed this with any of the MAGs that you recovered?

Response: As described in the previous response, we identified MAGs that carry the enriched genes and showed that SusD genes predominantly originated from Bacteroidetes MAGs (Fig. 5b, Supplementary Notes page 100, Results section lines 354-358).

Referee #3 (Remarks to the Author):

Wibowo et al. report shotgun metagenome sequencing on eight paleofeces samples

gathered from two sites in the southwestern USA and one site from Mexico. The authors used several methods to infer that much of the resulting DNA sequencing data was of ancient human origin, including carbon dating, microscopy analysis of dietary components, DNA damage profiling, analysis of host DNA found in the paleofeces, and community composition analysis. The authors heavily leverage MetaPhlAn2 to compare community composition between ancient human stool samples with modern human stool samples from American, Fijian and Mexican communities. Genome bins were also recovered from paleofeces metagenomes, revealing many novel species-level groups. The authors also performed a functional analysis on genes predicted from metagenome assemblies.

The methods used to generate these data appear to be robust, and the data generated represent an exciting contribution to the field. While many of the findings presented here are confirmatory for what has been inferred about ancestral human gut microbiomes based on study of traditional populations, the thorough analysis to ensure the human source and age of the samples makes the resulting data and analysis very valuable. However, the metagenome analysis was lacking in several key areas and significant revisions are necessary.

Comment: Read-mapping and relative abundance methods and data are missing **Response:** We thank the reviewer for pointing these out and have included these data in the manuscript as described below.

Comment: The authors included very little information regarding the methods and results of read- mapping analyses from mapping a sample's own reads on its own assembly. Details that need to be described for each paleofeces metagenome sample include:

-Minimum contig length included in the bowtie2 index for read-mapping (line 893)

Response: We have now included this description in the Methods section (lines 1034-1035): "For each sample, reads were mapped to contigs using Bowtie2 (version 2.3.5.1)[97] with default settings (no minimum contig length)."

Comment: -Percentage of assembled reads that were mapped onto these contigs

Response: We have now added these details in Supplementary Table 6 (Tab 1 for the ancient genomes, Tab 3 for the filtered ancient genomes, Tab 5 for the soil genomes, and Tab 7 for the Mexican genomes). The percentage of reads that mapped to all of the contigs in each sample is shown

in the column titled “% reads mapped to contigs”. Moreover, the percentage of reads that mapped onto each genome bin is shown in the column titled “relative abundance (% reads aligned to bin)”. The percentage of reads mapped onto all of the MQ/HQ genomes per sample is shown in the column titled “% reads aligned to MQ/HQ bins per sample”. We describe this calculation in the Methods (lines 1044- 1046): “The relative abundance of each reconstructed genome (Supplementary Table 6) was calculated by dividing the number of reads aligned to the genome by the total number of raw reads from that sample.” and lines 1049-1050 “The percentages across genomes from the same sample were summed to calculate the percentage per sample.”

Comment: -Minimum contig size used for MetaBAT2 genome binning
Response: We have now included this detail in the Methods section (lines 1036-1037): “The sorted BAM file was used for contig binning using MetaBAT2[10] with default parameters (minimum contig size=2.5kb), resulting in putative genomes.”

Comment: -Number and percentage of contigs binned using MetaBAT2 that appear in MQ or HQ genome bins
Response: We have now included these details in Supplementary Table 6 (Tab 1 for the ancient genomes, Tab 3 for the filtered ancient genomes, Tab 5 for the soil genomes, and Tab 7 for the Mexican genomes). Specifically, the number of contigs per genome bin is shown in the column titled “# contigs”. The percentage of contigs that appear in each genome bin is shown in the column titled “% binned contigs in bin”. The percentage of contigs that appear in all MQ/HQ bins per sample is shown in the column titled “% binned contigs in MQ/HQ bins per sample”. We describe this calculation in the Methods section (lines 1046-1047) “To calculate the percentage of contigs binned in each genome, the number of contigs per genome was divided by the number of contigs binned from the sample.” and lines 1049-1050 “The percentages for genomes from the same sample were summed to calculate the percentage per sample.”

Comment: -Number and percentage of base pairs from contigs binned using MetaBAT2 that appear in MQ or HQ genome bins
Response: We have now included these details in Supplementary Table 6 (Tab 1 for the ancient genomes, Tab 3 for the filtered ancient genomes, Tab 5 for the soil genomes, and Tab 7 for the Mexican genomes). Specifically, the number of base pairs per genome bin is shown in the column titled “Genome size (bp)”. The percentage of base pairs from contigs in each genome bin is shown in the column titled “% base pairs from binned contigs in bin”. The percentage of base pairs from contigs that

appear in all MQ/HQ bins per sample is shown in the column titled “% base pairs from binned contigs in MQ/HQ bins”. We describe this calculation in the Methods section (lines 1047-1050) “To calculate the percentage of base pairs from contigs binned in each genome, the genome size (in bp) was divided by the number of base pairs in the contigs binned from the same sample. The percentages for genomes from the same sample were summed to calculate the percentage per sample.”

Comment: -Describe the method used to calculate the coverage depth of each genome bin (Table S4, Tab1-coprolite genomes)
Response: We have now added this description in the Methods section (lines 1042-1044): “Coverage for each contig was calculated using the CheckM[78] “coverage” command, and coverage per genome was calculated by averaging the coverage profiles across all contigs within the genome.”

Comment: These data will allow the authors to answer questions such as:
-How much of the sequenced data (in % reads or % sequenced bp) can be accounted for by MQ or HQ genomes binned in this study
Response: The average percentage of reads aligned to the MQ/HQ bins per paleofeces is 23% (sd=7.2). For the filtered bins (contigs <1% damage removed), the average percentage is 11.5% (sd=9.4). Meanwhile, the average percentage is 5.97% for the soil samples (sd=6.5) and 25% for the Mexican samples (sd=12.5). These results are shown in Supplementary Table 6 (column titled “% reads aligned to MQ/HQ bins per sample”) and described in the Results section (lines 230-232): “We calculated the percentage of raw reads aligned to the genomes and found that on average, the MQ and HQ filtered genomes account for 11.5% (sd=9.4) of the total raw reads per sample (Supplementary Table 6).”

Comment: -Are the novel species-level genomes a major or minor component (abundance-wise) of the paleofecal metagenomes?
Response: The average percentage of reads aligned to the novel MQ/HQ pre-filtered genomes per paleofeces is 8.5% (sd=5). For the novel MQ/HQ filtered genomes (contigs <1% damage removed), the average is 3.3% (sd=1.7). These results are summarized in Supplementary Table 6 (column titled “% reads aligned to novel MQ/HQ bins per sample”) and described in the Results section (lines 259-261): “On average, the novel MQ and HQ filtered genomes constitutes 3.3% (sd=1.7) of the total raw reads per sample (in percentage of reads aligned) (Supplementary Table 6).”

Comment: Additionally, coverage depth information for each gene annotated by PROKKA should be incorporated into the analysis to calculate fold change between samples (see below).

Response: We thank the referee for the suggestions on how to improve our functional analysis. We re- performed our functional analysis as suggested by the reviewer as described below.

Comment: The authors should comment on the issues that arise when using MetaPhlAn2 (whose reference genomes largely come from industrialized countries) to estimate presence/absence and relative abundance of taxa in paleofecal samples. Other mapping analyses could be used to determine what % of paleofecal metagenome reads can be accounted for by:

-Genomes in the MetaPhlAn2 database

-Genome bins from the combined Pasolli, Almeida, and Nayfach papers

-Novel MQ+HQ species-level bins from all paleofecal samples

Response: We agree that there are caveats of using MetaPhlAn2 and have now pointed these out in lines 160-161 "This is a reference-based tool and only identifies taxa present in the reference database." and lines 209-210: "MetaPhlAn2 is a reference-based approach and its database mostly comes from industrialized samples, therefore the above analysis only identified those taxa present in its database."

However, even though MetaPhlAn2's reference database mostly comes from industrialized individuals, it is a powerful tool to better understand which of those taxa in the reference database are present in our samples. The challenge of mapping the raw reads to genomes from Pasolli, Almeida, and Nayfach papers and our ancient MAGs is that most of those genomes do not have clear taxonomic names, so it will be challenging to use them to map which taxa are present in our samples.

Additionally, MetaPhlAn2 aligns raw reads to species-diagnostic marker genes instead of whole genomes, and this allows for high confidence in taxonomic assignment. Aligning our reads to whole genomes will introduce a large amount of noise into our taxonomic assignments, as many reads will not align uniquely to one species or strain. For the purposes of this study and its specific research questions, we believe MetaPhlAn2 is the more appropriate classifier, despite its limitations.

Given these, we decided that MetaPhlAn2 is still best to use for identifying taxa present in the reference genomes. Meanwhile, we have added comments on some of the caveats of

using MetaPhlAn2 as

suggested by the reviewer and have performed an unbiased analysis with our *de novo* genome reconstruction.

Comment: It is also important to determine whether novel genome bins recovered in one paleofecal sample can be detected in other paleofecal samples from this study.
Response: This detail can now be found in Supplementary Table 6. In “Tab 4-paleo filtered rep bins”, the column titled “# Reconstructed genomes” shows the number of ancient genomes within each species-level genome bin (SGB). The column titled “# Samples” shows the number of paleofecal samples each SGB is detected in. Seven of the novel SGBs (SGB IDs 17_1, 24_1, 25_1, 119_1, 121_1, 157_1, and 161_1) are shared across multiple paleofecal samples.

We have also described this in the Introduction (lines 80-81): “Many of these novel SGBs belong to the typical human gut microbiome genera, and seven are shared across multiple paleofeces.” and in the Results section (lines 256-259): “The results reveal that 61 or 39% of the ancient gut SGBs are novel SGBs that are not represented in the current reference genomes (Fig. 3a, Supplementary Table 6), and seven of these novel SGBs are shared across multiple paleofeces.”

Comment: Calculating and reporting species novelty
The authors describe 45% of their medium and high quality bins as “novel” due to these bins having Mash distance >0.05 to MAGs in the Pasolli, et al. data set as well as the genomes present on NCBI. Using Mash distance as the lone metric for determining novelty is insufficient. Mash provides an estimate for ANI identity but is not exact. The strength of this estimate is in part determined by the **“sketch size”,** which the authors do not provide. The strength of the estimate is also weakened when genomes being compared are of less than high quality.

For a more rigorous quantification of novelty, the authors should use Mash as a means of clustering species at 90% identity, and then calculate exact ANI distances using ANImf or fastANI to determine if genomes exceed the 95% similarity threshold (this can be done using dRep, which the authors have used elsewhere in the paper).
Response: We modified our novelty quantification according to this feedback. We performed primary clustering with Mash (sketch size=1000, specified in lines 1063-1064) followed by secondary clustering using fastANI, as described in the Methods section (lines

1058-1068): “To determine whether each of the SGBs belong to a known microbial species, pairwise genetic distances were calculated between each of the representative genomes and each of the 388,221 reference microbial genomes. The

reference genomes included all human gut MAGs reconstructed in Almeida et al.[13] and Nayfach et al.[12] (catalogued in Almeida et al.[48]), and all MAGs reconstructed in Pasolli et al.[14], 80,990 genomes from NCBI GenBank database used as reference in Pasolli et al.[14], and MAGs from non-human primate gut metagenomes[49]. Mash distances[50] were calculated for all of the genomes using default settings (sketch size=1000). Subsequently, Average Nucleotide Identities (ANIs) were calculated using FastANI[51] for each ancient genome and its 100 closest reference genomes within 10% Mash distance. The dRep[52] “cluster” command was utilized to run FastANI[51] with the following settings: -sa 0.95 --S_algorithm fastANI. Bins with >95% ANI to at least one reference genome were classified as “known” SGBs and the rest were classified as “novel” SGBs.”

Comment: Finally, the authors should clarify that genomes <= 95% ANI from all others in a reference dataset are not "novel species", but rather "novel species-level groups distinct from those in the existing reference database". This is in line with how others have reported novelty based on MAGs (Pasolli, et al., 2019).

Response: We now use the terminology “species-level genome bins (SGBs)” throughout the text.

Comment: Functional analysis

The pipeline that the authors used for functional analyses involved 1) annotation of genes using PROKKA (based on the UniProtKB database), 2) (random?) rarefaction to 100,000 genes per sample,
3) determining the number of genes assembled from each sample with each functional annotation, 4) identifying the top 100 enriched genes in paleofeces HMP samples and annotating them with broad functional categories, and 5) using a 2-tailed Fischer’s test to determine enrichment of functional categories. In addition to a lack of methods describing how this pipeline was carried out, this approach has several distinct weaknesses.

First, this analysis has no consideration of gene abundance. The most abundant gene in a sample has the same weight as the least abundant gene, and both have an equal probability of being eliminated from analysis entirely during rarefaction. The analysis should consider gene abundance as well as existence, especially since similar genes in different genomes can assemble together to make one representative gene (which would

then only be counted once).

Second, it is not clear from the methods section how DESeq2 was run on the resulting table of gene counts, nor it is clear that DESeq2 is an appropriate tool in this circumstance. DESeq2 is designed for

RNA-seq read counts, not counts of numbers of assembled gene categories (which are likely non- normally distributed and may violate some of the statistical assumptions underlying DESeq2). Further, DESeq2 is notorious for generating many false positives (see Paulson et al. 2013 Nature Methods, and many others since). If DESeq2 is used, then the p value cutoff should be more like 0.000001, and the abundances of significant genes checked with a boxplot (on the log scale) to visually check if they are real or driven by one or two outliers.

Finally, it is not clear that the final Fisher test being performed is the best way to address functional differences. Not only is it relient on subjective annotation, but it's unclear that the final readout of “Are more genes of this category in the top 50 most enriched HMP genes vs Paleofeces genes?” is the right question to ask.

One way of addressing these concerns would be to map reads from all samples to a gene catalog and use this to calculate gene differential abundance. This would consider gene relative abundance and make for a more appropriate use-case for DESeq2 (with the caveats listed above). This gene catalog could include all genes assembled in this study, all CAZymes, and other existing gene catalogs. Genes could be annotated using a DIAMOND / BLAST search against UniRef, or with Prokka as is performed now. See (Li, J., Jia, H., Cai, X. et al. An integrated catalog of reference genes in the human gut microbiome. Nat Biotechnol 32, 834–841 (2014). https://doi.org/10.1038/nbt.2942) for an example.

Response: We appreciate this thorough feedback, all of which we believe we have now successfully integrated into our functional analysis. This has significantly strengthened our analysis. To address this comment, we modified our methods to take gene abundance into account as suggested by the referee. Briefly, we performed *de novo* assembly using MEGAHIT, annotated predicted genes using PROKKA, and clustered the genes from all of the samples using CD-HIT to produce a non-redundant gene catalog. Subsequently, we aligned the raw reads from each sample to the non-redundant gene catalog and calculated gene relative abundances per sample. To identify genes that are differentially

abundant between the different groups, instead of using DESeq2, we performed a two-tailed Wilcoxon rank-sum test. which is more conservative. These changes are reflected in the Methods section (lines 1146-1160) and the Results section (pages 14-16 and Fig. 5). As suggested, we also visualized the distribution of the relative abundances of the significant genes with boxplots to confirm that the enriched genes are not driven by outliers (Supplementary Notes pages 94-99).

Though we feel more confident in this improved methodology, we wish to note that some of the enrichment patterns detected in the previous analysis with DESeq2 are similarly observed with the

current method. For instance, in both analyses, many antibiotic resistance and Sus genes are highly enriched in the modern-day samples relative to the paleofeces. This consistency strengthens our confidence in these enrichment patterns.

Comment: Additional Specific Points:

-The authors describe how DNA damage patterns can be identified by mapping reads to a reference genome. Can the authors comment on how this would impact analyses such as assembly, read- mapping, and the generation of phylogenetic trees? Is it possible that ANI distances are being overestimated because damaged bases are contributing significantly to contig consensus sequences? If so, possibly modeling damage on simulated metagenomic data derived in silico from sequenced genomes (where the gold standard is known) could be used to correct ANI calculations, or establish a revised threshold.

Response: The damage levels in our samples are very low and the coverage per MAG is high. For the medium- and high-quality filtered genomes, the average coverage level is 90x (sd=108.4). Therefore, we expect that damage in the terminal bases of the reads would be masked after assembly and the MAGs should not contain a significant amount of damage in the consensus sequence. To more quantitatively test this, we performed a simulation using short-read sequencing data with varying GC contents, aDNA damage levels, sequencing depths, and DNA fragment lengths (lines 216-220 and pages 86-90). The results show that there is minimal effect of aDNA damage on the number of mismatches between the assembled genomes and the original reference genomes. Additionally, there is no enrichment of C-to-T substitutions (typical aDNA damage pattern) compared to other substitutions in the assembled contig consensus sequences.

Comment: -Line 94: Please provide more detail regarding what constitutes a “poor

assembly result". **Response:** We noticed a bimodal distribution in the number of MQ/HQ genomes reconstructed from our samples (Supplementary Table 1). For the eight final samples included in our analyses, the average number of reconstructed MQ/HQ genomes is 62 (sd=68). Meanwhile, for the four samples we excluded due to "poor assembly", the average number of reconstructed MQ/HQ genomes is 3 (sd=2). These four samples have only either 0 or 1 HQ genomes and ≤5 MQ genomes per sample. Therefore, we decided to include samples with high number of reconstructed genomes and exclude samples with low number of reconstructed genomes. We have now specified what we considered a "poor assembly result" in the Results (lines 95-97): "We excluded four paleofeces with poor assembly results (≤1 high- quality genomes and ≤5 medium-quality genomes reconstructed; Supplementary Table 1)".

Comment: Also consider providing metrics such as mean read length following preprocessing, assembly N50, # contigs bigger than the minimum size used for MetaBAT2 binning, # base pairs in contigs bigger than min size for MetaBAT2 binning, etc.

Response: We have now incorporated these details into the manuscript as follows.

- We have now provided details on the mean read length following preprocessing in Supplementary Table 1 under the column titled "mean read length after pre-processing (bp)".
- Assembly statistics per metagenome are now shown in Supplementary Table 1 and is described in the Methods section (lines 1028-1030): "Assembly statistics (number of contigs, number of base pairs in contigs, contig N50, contig L50, and the longest contig) were calculated using BBMap's (version 38.86) statswrapper.sh function (https://sourceforge.net/projects/bbmap/) with default parameters (Supplementary Table 1)."
- We have now included details on the number of contigs bigger than the minimum size used for MetaBAT2 binning in Supplementary Table 1 under the column titled "n_contigs>= 2500bp (min contig size for binning)".
- We have now added details on the number of base pairs in contigs bigger than the minimum size for MetaBAT2 binning in Supplementary Table 1 under the column titled "total bp in contigs with length >=2500bp".

Comment: -Line 259: It is stated that novel ancient genomes have long phylogenetic branches, and this does appear to be supported in Fig 4. It would be nice if this could be quantified (e.g. using a metric like "added branch length").

Response: As Referee #2 pointed out, technical issues (e.g. incomplete genomes,

chimera, misalignment) could contribute to long phylogenetic branches. Our visual inspection of the multiple sequence alignment files suggests that these long branches are not due to misalignment (described in pages 90-91). However, we agree with Referee #2 that we cannot exclude all possible artefacts, so we have now modified the text to not overemphasize on whether the long phylogenetic branches suggest that the novel genomes are truly ancient. Specifically, we modified these previous sentences "For *Prevotella* (Fig. 4b), as well as the other genera (Fig. 4a, Extended Data Fig. 9), the novel ancient genomes have long phylogenetic branches. This supports our findings that these genomes are highly distant from the reference genomes." to "Phylogenetic trees for genera that are assigned to many ancient bins (*Bacteroides*, *Prevotella*, and *Ruminococcus*) (Fig. 4b, Extended Data Fig. 9) show that the novel ancient genomes do not cluster closely with the reference genomes." (lines 286-288).

To more quantitatively assess distance between the ancient genomes and the modern-day reference genomes, we performed a tip dating analysis for *M. smithii* using two filtered (contigs <1% damage were removed) ancient *M. smithii* genomes from samples UT30.3 (1947 +/- 30 BP) and UT43.2 (1994
+/- 26 BP) as tip calibrations. The results indicate that using ancient genomes for calibrating *M. smithii* phylogenies, we could evolutionarily match previous studies on one of its sister species, *M. oralis* (Weyrich et al., Nature, 2017). This further supports the quality of our reconstructed genomes and the potential of utilizing ancient genomes reconstructed from paleofeces to study the evolutionary history of the gut symbionts. These results are described in lines 290-312 and shown in Fig. 4c and Extended Data Fig. 10, and the methodology is described on pages 54-55.

Comment: -The paper would benefit from a more comprehensive analysis of genes relevant to complex carbohydrate degradation (e.g., dbcan) since the authors discuss enrichment of genes relevant to carbohydrate binding and import (susC,susD) in Bacteroidetes but do not discuss differential abundance of other relevant genes or gene clusters. Previous papers comparing traditional to industrialized microbiota highlight that plant polysaccharide-related CAZymes are more abundant in traditional populations, while mucin-degrading CAZymes are enriched in industrialized. A more comprehensive analysis along these lines could test whether similar findings hold in ancient samples and thus contribute to the overall breadth and relevance of the findings.

Response: We performed a CAZyme analysis as suggested. The methods are described in lines 1172- 1183 and the results are shown in Fig. 5c and described in lines 360-370: "To delve into differences in these carbohydrate degradation genes, we predicted CAZy

enzymes[66] from the PROKKA output files (see Methods), performed two-tailed Wilcoxon tests with FDR correction, and manually curated the CAZy families with broad substrate categories. We excluded CAZy families enriched in the soil samples relative to the modern samples. The results reveal a similar enrichment pattern in the paleofeces and in the non-industrial samples compared to the industrial samples (Fig. 5c). For instance, starch- and glycogen-degrading CAZy families are enriched in the paleofeces and the non-industrial samples, while mucin- and alginate-related CAZy families are enriched in the industrial samples. Altogether, our data reveals molecular functions that are distinct between the ancient and the modern gut microbiome and the taxa they come from within our samples. The results indicate more shared features between the paleofeces and the non-industrial gut microbiome relative to the industrial gut microbiome."

Comment: Minor points

-Line 93: "Assembled" should read "binned"

Response: We have made this correction.

Comment: -Line 214: Were the Nayfach et al. bins included in the 385,236 bins used for MASH comparison? The total number of bins indicates that it was, but that paper is not cited in that instance. **Response:** The reference genomes were collected from Almeida et al. (bioRxiv, 2019), which catalogues human gut MAGs reconstructed in Almeida et al. (Nature, 2019) and Nayfach et al. (Nature, 2019). We have clarified this in the text (lines 236-239 and lines 1060-1063): "The reference genomes included all human gut MAGs reconstructed in Almeida et al.[13] and Nayfach et al.[12] (catalogued in Almeida et al.[48]), all MAGs reconstructed in Pasolli et al.[14], 80,990 genomes from NCBI GenBank database used as reference in the study, and MAGs from non-human primate gut metagenomes[49]."

Comment: -Line 229: "Out of these 71 bins, 20 have low variance." It is unclear what this means. **Response:** We were referring to low damage variance across contigs, as described in lines 244-245: "Further, 62 of the 181 high-damage genomes have low damage variance across contigs (see Methods)." However, we have decided to remove the sentence "Out of these 71 bins, 20 have low variance." from the text since our subsequent analyses focus on all of the high-damage genomes, and not only the genomes with low damage variance across contigs.

Comment: -Line 1142: should read "milled maize" rather than just "milled"

Response: We have corrected this.

Comment: -Figure 1, sub-panel B: report confidence intervals of mean corrected dates

Response: We have included the confidence intervals in Fig. 1b and in Supplementary Table 1, Tab1.

Comment: -Figure 3g: edit x axis to not include half values, can’t have 0.5 or 1.5

Response: We have edited the x axis to not include half values.

Comment: -Extended Data Fig. 10: There is a random letter “e” floating to the right of “Autoinducer-2 kinase"

Response: The Figure has been replaced with plots from the new functional analysis results.

Referee #4 (Remarks to the Author):

Wibowo et al. report a de novo assembly of the ancient gut microbiome from 8 human paleofeces 1-2 thousand years old. They report that these are more similar in microbial composition to the gut microbiome of non-industrialized humans than to of industrialized humans. If true this is very important and interesting given recent years research showing the importance of our gut microbiome relative to varies health conditions. However, this type of reseach face some specific challenges of which some, but not all, are convisingly addressed by the authors:

1. The sequences are endogenous and ancient: The damage pattern reported by the authors suggest its the case, although the length distribution is unusual for ancient DNA. The reads (<100bp) are unusually long. Still, I buy this argument given we know very little on DNA preservation in microbes under varies preservation conditions.

Comment: 2. The sequences are from the human gut and not from the surrounding environment: This is a crucial part as feces will not only be contaminated by contemporary DNA but with DNA from environmental microbes occupying the faces shortly after its delivered. Thus you will find a mixture of ancient microbial sequences of environmental and gut origins. How can you separate these? The authors argues some are typical of gut but not of environment, but what do we know about the surrounding environment microbes at the site back in time (we have only poor understanding of present settings)- nothing. Furthermore, with short reads how do we know that a given sequence if from a given gut microbe and not from a closely related environmental microbe of the past - We don't.

The authors needs too convincingly show that they can address point 2. A number of peoples have considered doing what the authors do, but have abandoned the idea simply because there has been no way to convincingly address point 2. For the authors to make a strong claim they need to solve this problem.

Response: We thank the referee for this thorough feedback. To check for possible contamination from environmental microbes of the past, we have now compared our paleofeces to both modern soil samples and a diverse set of archaeological sediment samples from different time periods, as shown in our SourceTracker analysis (Extended Data Fig. 1) and clustering analysis (Fig. 1c). As described in the Methods (lines 994-996), "These environmental samples include the 3 soil samples collected in this study, 40 Pleistocene sediment samples[77], and 7 Holocene human-associated sediments (which overlap in age with our paleofeces) from CoproID[25]." (also see Supplementary Table 9). The results show that the taxonomic composition of the paleofeces is significantly different from both the modern soil samples and the archaeological sediment samples (Fig. 1c, Extended Data Fig.1). In contrast, compared to a large set of non-industrial (371 samples from five countries) and industrial (418 samples from three countries) gut microbiome samples, our taxonomic analysis reveals that the paleofeces falls within the diversity of the non-industrial gut microbiome (Fig. 2d). The large number of samples originating from various populations strengthens the validity of these results.

We agree with the reviewer that with short reads, determining whether a given sequence is from a gut microbe or an environmental microbe may be difficult. However, with our medium- and high-quality *de novo* assembled genomes, we have long contigs (mean=20,311 bp, sd=20,683 bp) with very high coverage depths (mean=90x, sd=108.4), and we have higher confidence in these results. Our results reveal that compared to 388,221 reference microbial genomes from various sources, the majority of our reconstructed ancient genomes (97%) are most closely related to gut bacteria (lines 239-242, lines 1068-1071, Fig. 3a, Supplementary Table 6). Many of them also belong to typical human gut microbiome genera, as described in lines 266-269: "The most abundant annotated genera for the 181 high-damage gut genomes include *[Eubacterium], Prevotella, Ruminococcus, Treponema,* and *Blautia* (Fig. 3f). These are typical human gut microbiome genera, which supports that the curated genomes, including the novel SGBs, are gut microbes." Even though we do not know the exact genomes of soil microbes of the past, we do not expect those to resemble gut genomes more than soil genomes. These *de*

novo reconstructed genomes provide stronger evidence that our paleofeces contain many true gut microbes, despite the small amount of potential contamination.

Moreover, we have now directly compared pairwise Mash distances for two comparison groups: 1) between our ancient genomes and 286,933 reference gut microbial genomes (Almeida et al., bioRxiv, 2019), and 2) between our ancient genomes and 1,442 reference soil microbial genomes (all genomes in JGI GOLD labeled as soil). To match the sample sizes, we randomly sub-sampled 5,000 Mash distances 1,000 times for each comparison group. We calculated the proportion of Mash distances less than 0.10 for each permutation and performed a Wilcoxon two-tailed test to compare the two groups. The results show that the proportion of Mash distances less than 0.10 is significantly lower in the first comparison group (between the ancient genomes and the soil genomes) (mean=4e-06) relative to the second comparison group (between the ancient genomes and the reference gut genomes) (mean=0.0011714, $p<2.2e-16$). This further supports that our ancient genomes are significantly more similar to gut microbes compared to soil microbes

Overall, we took careful steps to ensure that the samples we analyzed were minimally contaminated and strictly removed potential environmental contaminants from our analyses. First, the paleofecal materials we processed for DNA extraction and sequencing were taken from the core of the paleofeces, which would have been least impacted by soil contamination (lines 876-878). Second, we excluded paleofeces with high environmental contamination levels according to SourceTracker (lines 97-98, Extended Data Fig. 1). Third, to ensure the enriched genes and species are not soil contaminants, we removed genes and species enriched in the soil samples relative to the modern samples (Fig. 2d, Fig. 5a, Fig. 5c). Finally, we explicitly labeled our MAGs as gut or soil bacteria based on their genetic distances to modern gut and soil bacteria (97% of the filtered MAGs are most closely related to gut microbes) (lines 239-242, lines 1068-1071, Fig. 3a, Supplementary Table 6). Altogether, we believe we have comprehensively performed multiple layers of filtering steps to minimize the effect of soil contamination on our results. We have also transparently shown that our samples contain some environmental contamination, yet the level of contamination is quite low. The final set of paleofecal samples, genomes, and genes have been extensively curated, and even though there could still be minimal soil contamination, the results show that there is still much we can learn about the ancient gut microbiome from these samples.

Reviewer Reports on the First Revision:

Referee #1 (Remarks to the Author):

I think this is a great study in regards to studying the field of paleofeces (and studying bacteria in old samples in general) and my main concerns in regards to the interpretation of the data have been addressed by taking a much wider look. Additional analyses and figures have really added a lot to the manuscript.

Points of interest:
In figure 5c, it can be seen that that the chitin CAZyme category is enriched in paleofeces compared to both non-industrial and industrial samples. This looks like an interesting finding worth mentioning yet I do not see this anywhere in the text connecting this to their specific diet. Initially I thought of a higher consumption of insects (like I find in some monkey-poo research I'm affiliated with). A higher consumption of insects is indeed something that is somewhat substantiated by the microscopic analysis (lines 1387-1388 & 1457) but after reading the dietary analysis results (1367 and onwards) and googling some more it is probably very safe to say that this is largely, or in addition, caused by the consumption of Ustilago maydis fruiting bodies (huitlacoche), and perhaps (to a small degree?) to the consumption of mushrooms (lines 1465-1466). I think it worth connecting these chitin CAZyme findings & microscopic analyses & local culinary customs (Pueblo cuisine) which have been around for a long time.

Secondly, you see a shift of the starch/glycogen CAZyme category, going from medium to high to low (paleofeces/non-industrial/industrial). It seems logical that in non-western agricultural societies that the consumption of starch/glycogen will be higher than in these paleofeces samples (as the diet of the producers of paleofeces was probably more diverse, see chitin above and various bits from the "dietary analysis" part in the methods section and lines 1367-1461) and it was already known that it is much lower in industrial societies (where you see a higher consumption of simpler substrates).

Can these Chitin CAZy families btw be linked/associated with specific microorganisms? Can a link be made with the number of Ustilago maydis spores found in samples? Different datasets become more interesting if you can show that they are part of a larger pattern.

I think you should allow yourself some room in interpreting this "sequencing dietary" and "microscopic dietary" data and also mention it in the discussion. Especially the chitin-finding really links it up with old local traditions, again showing that what is reported is "real".

Minor comments:
The Spirochaetes/Treponema finding is great/convincing but lines 173-175 and lines 203-204 about Spirochaetes and Treponema being lost almost effectively report on the same thing. It's a bit double (like this sentence). Perhaps not mention Spirochaetes being lost at the phylum level but mention them together with Treponema when you arrive at the species level?

Differences were found at the phylum and family level for Verrucomicrobia / Verrucomicrobiaceae yet nothing is mentioned at the species/genus level. Where is Akkermansia, or are we dealing with multiple Verrumicrobiaceae?

Lines 354-358. You mention Bacteroidetes here (phylum), but should you not be going down to the family level and say Bacteroidaceae, or Bacteroides, as mentioned in lines 383-385? While Prevotellaceae might have many of the same qualities they are not enriched in industrial samples; I think it is better to also be more specific here.

Lines 1489-1490: Calling something "prebiotic" just because it increases the number of Bifidobacteria, especially in such as varied diet as the people at Zape had, is kind of a miss in my

book. Many things they ate could probably be called “prebiotic” by industrial standards. Perhaps replace “prebiotic” by “bifidogenic”? Or, “… would have a bifidogenic, and by todays standards, prebiotic effect on the gut microbiome.”

Line 1498: Again, perhaps replace prebiotic with “bifidogenic” or “bifidogenic/prebiotic”?

Possible minor errors (though I might be wrong):
Line 397: “is” attainable?
Line 398: remove “the”?
Line 410: add “the” in front of absence?

Referee #2 (Remarks to the Author):

Overall, I’m satisfied with the authors responses to my comments. I have a few additional minor comments / suggestions for the authors consideration (revised ms line numbers):

Line 199: The species level analysis of enriched taxa in the paleofaeces made me remember Bifidobacterium longum subsp. infantis, a species thought to be important for the normal development of the infant gut microbiome which has become “extinct” in the modern westernised gut – see https://www.ncbi.nlm.nih.gov/pmc/articles/PMC7352178/
Was this species present in the paleofaeces?

Lines 349-352: Are these antibiotic resistance genes chromosomally encoded? Plasmid borne AbR genes are notoriously difficult to associate to their hosts via MAGs, so this caveat should be noted in this analysis. Are there other AbR genes in the metagenomes not associated with MAGs pointing to potential plasmid borne genes? And are such untethered AbR genes mostly absent in the paleofaeces?

Lines 529-530, ref 50: This preprint is now published: https://www.nature.com/articles/s41587-020-0603-3

Line 1066: What alignment fraction (AF) was used with fastANI? This is the other important parameter setting for ANI calculations and it should be mentioned somewhere.

Line 1519: I thank the authors for performing the %read mapping analysis against the different faecal samples showing the expected relative mapping, however, why is there such a low % of reads (<4%) mapping to the MetaPhlan2 database? Typically 50-90% of faecal shotgun reads map to reference genome databases. Presumably this is a function of the database itself and because MetaPhlan2 doesn’t map to whole genomes, but this should be explained here (even so, this % read mapping seems remarkably low).

Referee #3 (Remarks to the Author):

The authors have conducted a nice revision, addressing our major points related to read-mapping and relative abundance methods, as well as with calculating and reporting species novelty.

New aspects of functional analysis
• In the heat-map there appear to be relative abundance values at 1 - were these values scaled at all prior to plotting? If so, please include that in the methods section.
• The methods section states that gene relative abundance was calculated in a way similar to RPKM (reads per kilobase million), but potentially without the same units. Because RPKM is a common measure in bioinformatics literature, it would be beneficial to calculate relative abundance in terms of RPKM and report the values as such (have the heatmap show actual RPKM values on a

log scale, not unitless scaled values).
• The analysis in Figure 5b is very confusing. Is each column an organism, or a taxa? It appears that some organisms are being counted multiple times at different taxonomic levels (e.g. in the figure on the left, the species and genus of Prevotella all have the same count). Please either remove this sub panel (which would be fine- the figure is already large without it) or add significantly more explanatory text to the figure caption / results / methods.
• The enrichment of transposases is super interesting. Would it be possible to determine if any genes are enriched near these transposases? Is anything known about the particular transposase families that were recovered?

The authors present a new analysis of M. smithii MAGs. Not sure this analysis is appropriate or required for this paper. Specifically, the authors should clarify (i) how the ancient genomes change what the analysis would have yielded otherwise (ii) any interesting implications of this analysis for the ancient genomes, M. smithii, human migrations, microbial evolution, etc.
The results presented seem to require a bit of clarification:
L..297-99 "and that M. smithii began to diversify ~85,000 years ago with a 95% highest posterior density (HPD) interval 51,000 ~ 128,000 years (Fig. 4c). This timeline is moderately earlier than the one of its sister species M. oralis (HPD: 112.000 - 143.000 years)"
• Don't the authors mean later rather than earlier? (after)
• How do ancient strains, which may be intermediates of others fit into a tree like this, where divergence is implied?

Referee #4 (Remarks to the Author):

By carrying out comparisons between the microbial composition of sediment samples from the study site and those of the coprolites I think the authors have done what is currently possible addressing my concern regarding environmental contamination of the past. One thing I think is worth reporting is a comparison of the damage and length distribution of the ancient soil microbes and the ancient microbiome from the same site (can't see the authors have done this). These are expected to be fairly similar and if this is indeed the case it would put some strength to their argumentation that they can indeed use the comparative approach for separating the two fractions and that the soil microbes they compare are indeed ancient.

Referee #5 (Remarks to the Author):

In this study, Wibowo and colleagues reconstruct a collection of ancient microbial genomes from several ~1000yr-old human paleofeces samples. In this review, I focused only on the SNP and molecular clocking analyses that the authors have added in their revision. I think these are largely in the right direction, though I have a few technical suggestions for improvement:

(1) Since the two ancient M smithii genomes were obtained from paleofeces from the same site, it seems like it would be difficult to exclude the possibility that they come from the same person --- or at the very least, from individuals that share a common household. As such, I think it is important to allow for the possibility that the two M smithii genomes share a recent common ancestor for epidemiological (rather than evolutionary) reasons. In this light, the SNP analysis on p.92 is still very informative for ruling out DNA damage artifacts (which should occur independently in the two samples), but I don't think it supports the conclusion that the ancient samples are particularly distant from the modern strains (at least, not any more than the modern strains are from each other), since the authors' control does not account for the close relatedness of the ancient samples.

I think the basic point could be made in a more robust way by simply re-running some the same

phylogenetic analyses (see point 2 below) using the inferred common ancestor of the ancient strains (i.e., SNPs that are shared by both ancient strains, but not necessarily absent in the modern strains).

(2) One could argue that the phylogenetic model used for the divergence estimates is not quite appropriate for recombining populations of bacteria (see, e.g. Vaughn et al Genetics 2017), though it is an understandable choice to facilitate comparisons with previous divergence estimates from Weyrich et al (Nature, 2017). I think this section could be improved by supplementing the existing BEAST analysis with a more lightweight approach, which simply reports the raw sequence divergence between the different pairs of strains.

Given the results in Fig. 4C, it would be informative to split the M smithii strains into 3 groups: the two ancient genomes (A), the modern genomes plotted above the ancient genomes in Fig. 4C (M1), and the modern genomes plotted below the ancient genomes (M2). One could then plot all sequence divergences between A & M2 strains, M1 & M2 strains, etc. In the strict clock model, the phylogenetic signal used for the dating would then show up as a systematic difference between the d(A,M2) and d(M1,M2) divergences:

d(M1,M2) - d(A,M2) ~ (substitutions per year * ~1000 yrs)

which calibrates the remaining genetic distances in units of years. The results would ideally be consistent with the dates inferred in Fig. 4C, while any large discrepancies from the BEAST model would immediately jump out. Repeating this analysis for the common ancestor of the ancient strains mentioned above would further support the claim that DNA damage is not severely biasing this signal.

Author Rebuttals to First Revision:

Response to Referees' Comments for Manuscript 2020-02-01803A
"Reconstruction of ancient microbial genomes from the human gut"

We thank the referees for their thoughtful feedback and comments towards improving the manuscript.

In the revised manuscript, first, we have included additional discussion about the sequencing results and the microscopic dietary analysis. Second, we have now incorporated damage pattern analysis for the soil samples from Boomerang Shelter. Third, we added details about our tip dating analysis and validated our BEAST2 results by calculating raw sequence divergences between the different *Methanobrevibacter smithii* genomes. Fourth, we converted our gene relative abundance values to RPKM, analyzed whether antibiotic resistance genes are found on plasmids or chromosomes in the MAGs, and identified genes that are often found near transposases in our samples. Lastly, we have modified the text and added or removed details as suggested by the reviewers.
To comply with the formatting guide, we cut down the main text from ~5,000 to ~3,000 words and from five to three figures. We moved a large amount of the text and Figs. 1, 3a, 3c-e, 3g, 4b, and 5b to Extended Data and Supplementary Information. We also removed our sex determination analysis in order to minimize inclusion of human DNA information when not central to the results of the manuscript.

Additionally, as a space-saving measure we removed the phylogenetic tree for *Bacteroides*, because the other two phylogenetic trees (*Prevotella* and *Ruminococcus,* now in Supplementary Fig. 6) are sufficient to convey our message. Following referee #5's comment, we excluded our SNP analysis (in page 92 of the previous submission) from the current revised manuscript.

Below are our specific responses to the referee's comments.

Referee #1 (Remarks to the Author):
I think this is a great study in regards to studying the field of paleofeces (and studying bacteria in old samples in general) and my main concerns in regards to the interpretation of the data have been addressed by taking a much wider look. Additional analyses and figures have really added a lot to the manuscript.

Points of interest:
Comment: In figure 5c, it can be seen that that the chitin CAZyme category is enriched in paleofeces compared to both non-industrial and industrial samples. This looks like an interesting finding worth mentioning yet I do not see this anywhere in the text connecting this to their specific diet. Initially I thought of a higher consumption of insects (like I find in some monkey-poo research I'm affiliated with).

A higher consumption of insects is indeed something that is somewhat substantiated by the microscopic analysis (lines 1387-1388 & 1457) but after reading the dietary analysis results (1367 and onwards) and googling some more it is probably very safe to say that this is largely, or in addition, caused by the consumption of Ustilago maydis fruiting bodies (huitlacoche), and perhaps (to a small degree?) to the consumption of mushrooms (lines 1465-1466). I think it worth connecting these chitin CAZyme findings & microscopic analyses & local culinary customs (Pueblo cuisine) which have been around for a long time.
Response: We agree with the reviewer that this result should be more clearly highlighted and discussed; we have added details about the chitin CAZyme enrichment in the Results (lines 244-250): "Additionally, chitin-degrading CAZy families are enriched in the paleofeces relative to both the nonindustrial and the industrial samples. This is in accordance with our microscopic dietary analysis that identified chitin sources, including remains of *Ustilago maydis*, mushrooms, and insects, in the paleofeces (Supplementary Information section 3). These foods were commonly part of ancient Pueblo and Great Basin diets[36]. The chitin CAZymes are prevalent in MAGs within Oscillospiraceae, Lachnospiraceae, and Clostridiaceae families (Supplementary Information section 14)."

Comment: Secondly, you see a shift of the starch/glycogen CAZyme category, going from medium to high to low (paleofeces/non-industrial/industrial). It seems logical that in non-western agricultural societies that the consumption of starch/glycogen will be higher than in these paleofeces samples (as the diet of the producers of paleofeces was probably more diverse, see chitin above and various bits from the "dietary analysis" part in the methods section and lines 1367-1461) and it was already known that it is much lower in industrial societies (where you see a higher consumption of simpler substrates).
Response: We added this description in the Discussion section (lines 265-267): "In contrast, the paleofeces and the non-industrial samples are enriched in starch-/glycogen-degrading CAZymes (Fig. 3b; likely due to a higher consumption of complex carbohydrates relative to simple sugars) and mobile genetic elements (Fig. 3a)."

Comment: Can these Chitin CAZy families btw be linked/associated with specific microorganisms?
Response: We have now mapped the CAZy families to taxonomies, as described in Supplementary Information section 14 (pages 116-117): "CAZy analysis was performed as described in the Methods section for all of the MQ/HQ filtered ancient MAGs, the MQ/HQ Mexican MAGs, and 1,451 representative MAGs from Pasolli et al.[14] that were reconstructed from the metagenomes used in our study. For each sample type (paleofeces/industrial/non-industrial), we plotted the number of MAGs within each taxonomic annotation that carry the chitin CAZymes (CBM14, CBM18, CBM19, CBM54, CE4, GH18, GH5_11, GH5_44, and GH8) (Supplementary Fig. 21)."

The results are shown in Supplementary Fig. 21 (attached below) and described in lines 249-250: “The chitin CAZymes are prevalent in MAGs within Oscillospiraceae, Lachnosipraceae, and Clostridiaceae families (Supplementary Information section 14).”

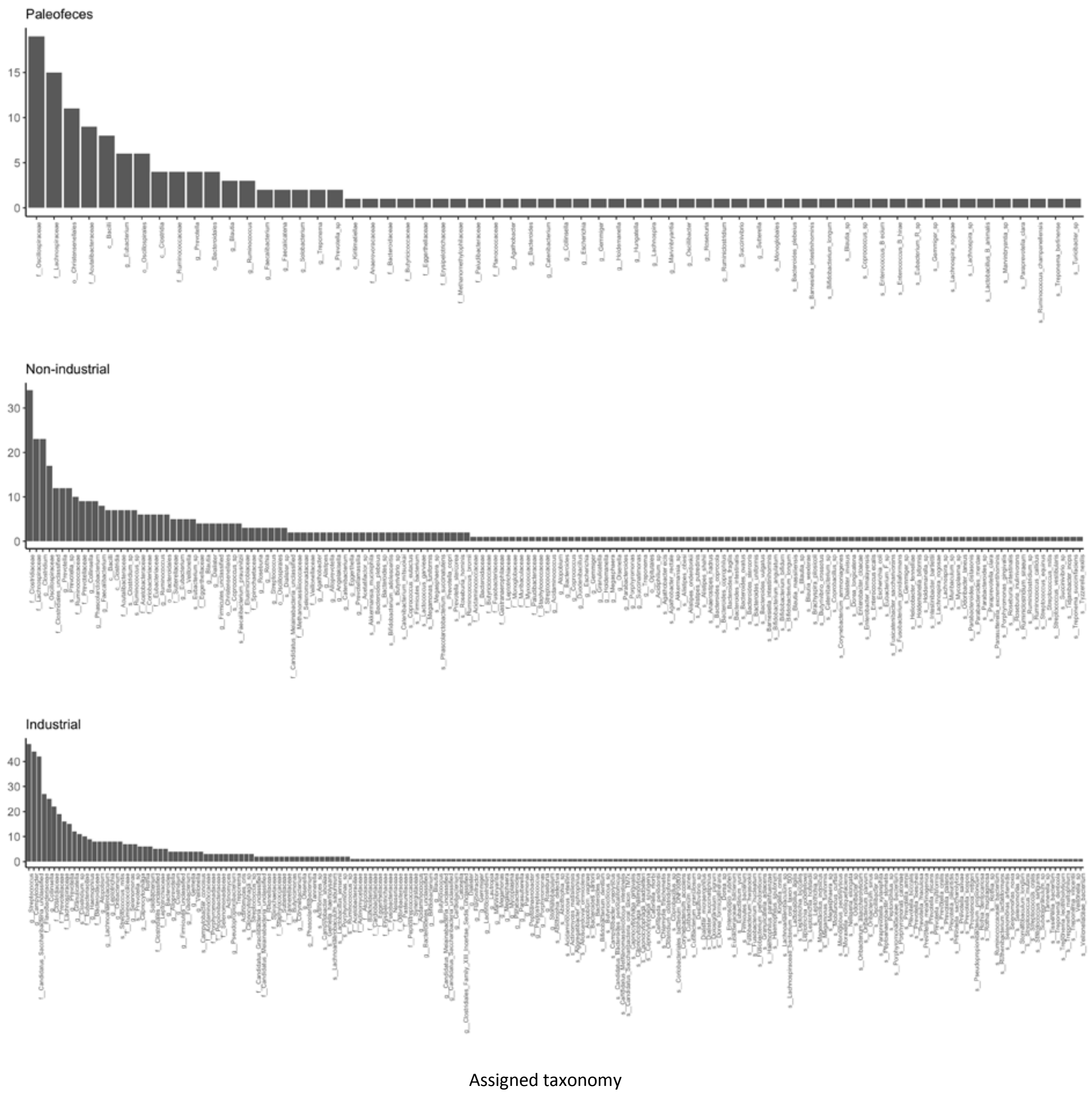

Supplementary Fig. 21. Ancient, non-industrial, and industrial SGBs containing chitin-degrading CAZymes (related to Fig. 3). Plots show the number of MAGs within each taxonomic annotation that carry chitin-degrading CAZymes.

Comment: Can a link be made with the number of Ustilago maydis spores found in samples? Different datasets become more interesting if you can show that they are part of a larger pattern.

Response: For most of the samples, the sizes of the samples used for microscopic analysis were so small that we were not able to determine accurately the number of *Ustilago maydis* spores per sample. We were only able to determine the percentage of samples containing *Ustilago maydis* spores per site (see attached tables below, also shown in Supplementary Table 2). We were not able to use this data to correlate the number of *Ustilago maydis* spores with the abundance of chitin CAZymes.

nature portfolio

Arid West Cave Paleofeces Results of eight samples	
Component	#positive/8
**Plant and Fungal Food**	
Maize, milled	7
Rocky mountain beeweed greens	5
Amaranth, milled	1
Cactus cladode	2
Physalis fruit	1
Maize kernals	2
Lycium fruit	1
Wild grass caryopses	1
Tansymustard leaves	2
Greaswood (*Sarcobatus*) staminate flowers	1
Ustilago maydis	1
**Animal Remains**	
Unidentifiable hair	1
Lagomorph hair	1
Animal bone and lagomorph hair	1
Grasshopper/cricket exoskeleton	1

Boomerang Cave Paleofeces Results of eight samples	
Component	Boomerang Cave % components (17)
**Plant and Fungal Food**	
Achnatherum caryopses	1
Cactus cladode	3
Chenopodium seeds	1
Greaswood (*Sarcobatus*) staminate flowers	2
Maize, milled and whole kernal	1
Maize pollen	3
Mustard greens	2
Rocky mountain beeweed greens	3
Ustilago maydis	1

Total Components Both Sites			
Component	Arid West Cave % components (28)	Boomerang Cave % components (17)	Data for both sites % components
**Plant and Fungal Food**			
Achnatherum caryopses	0	6	3
Amaranth, milled	4	0	2
Cactus cladode	7	18	13
Chenopodium seeds	0	6	3
Greaswood (*Sarcobatus*) staminate flowers	4	13	9
Lycium fruit	4	0	2
Maize kernals	7	0	4
Maize, milled	25	0	13
Maize, milled and whole kernal	0	6	3
Maize pollen	0	18	9
Mustard greens	0	12	6
Physalis fruit	4	0	2
Rocky mountain beeweed greens	19	18	19
Tansymustard leaves	7	0	4
Wild grass caryopses	4	0	2
Ustilago maydis	4	6	3
**Animal Remains**			
Animal bone and lagomorph hair	4	0	3
Grasshopper/cricket exoskeleton	4	0	3
Lagomorph hair	4	0	3
Unidentifiable hair	4	0	3

Comment: I think you should allow yourself some room in interpreting this "sequencing dietary" and "microscopic dietary" data and also mention it in the discussion. Especially the chitin-finding really links it up with old local traditions, again showing that what is reported is "real".
Response: We thank the referee for this suggestion and have added more discussion on the functional analysis and the microscopic dietary results, including discussion on the chitin CAZymes, in the Results (lines 244-250) and Supplementary Information section 14 (pages 116-117).

Minor comments:

Comment: The Spirochaetes/Treponema finding is great/convincing but lines 173-175 and lines 203-204 about Spirochaetes and Treponema being lost almost effectively report on the same thing. It's a bit double (like this sentence). Perhaps not mention Spirochaetes being lost at the phylum level but mention them together with Treponema when you arrive at the species level?
Response: To reduce redundancy, we have modified our description about Spirochaetes and

Treponema succinifiaciens as follows: - In the description about the phyla, we removed the following sentences: “This phylum has been proposed to be a key member of the ancestral human gut microbiome that has been lost in industrialized populations. To date, spirochaetes have been identified in the gut microbiota of nonhuman primates[41–43], non-industrial populations[4,44], and ancient humans[16], but they are absent from most industrial populations.”

- Now, we only mention Spirochaetes briefly, together with the other phyla that are enriched in the paleofeces (lines 113-115): “Firmicutes, Proteobacteria, and Spirochaetes are significantly less abundant in the industrial samples relative to the paleofeces (p=0.003, 0.002, $2.8x10^{-45}$) and the non-industrial samples (p=$2.5x10^{-16}$, $1.7x10^{-30}$, $3.6x10^{-93}$).”

- In the species-level description, we now elaborate on how both *Treponema succinifaciens,* and Spirochaetes in general, are lost in industrial populations (lines 134-138): “The spirochaete *Treponema succinifaciens* (p=$2.4x10^{-14}$, $1.1x10^{-117}$) is enriched in both the paleofeces and the nonindustrial samples relative to the industrial samples. *T. succinifaciens*, and more generally the phylum Spirochaetes (Fig. 1a), have been proposed to be lost in industrial populations[4]. These results support that the industrial human gut microbiome has diverged from its ancestral state[8,9].”

Comment: Differences were found at the phylum and family level for Verrucomicrobia / Verrucomicrobiaceae yet nothing is mentioned at the species/genus level. Where is Akkermansia, or are we dealing with multiple Verrumicrobiaceae?
Response: As the reviewer pointed out, *Akkermansia muciniphila* is enriched in the industrial samples relative to both the non-industrial samples and the paleofeces (Fig. 1d, Supplementary Table 3). We have now added this description in lines 129-132: “Species enriched in the industrial samples relative to both the paleofeces and the non-industrial samples include *Akkermansia muciniphila* (two-tailed Fisher’s test, FDR correction, p=$2.2x10^{-2}$, $9.8x10^{-30}$) and members of the *Alistipes* and *Bacteroides* genera (Fig. 1d, Supplementary Table 3).”

Comment: Lines 354-358. You mention Bacteroidetes here (phylum), but should you not be going down to the family level and say Bacteroidaceae, or Bacteroides, as mentioned in lines 383-385? While Prevotellaceae might have many of the same qualities they are not enriched in industrial samples; I think it is better to also be more specific here.
Response: We mentioned Bacteroidetes because the MAGs containing these glycan degradation genes are not only from *Bacteroides* or Bacteroidaceae, but also from other members of Bacteroidetes, including *Prevotella* and Prevotellaceae. Additionally, glycan-degrading capacity is found in many different members of the Bacteroidetes phylum, not just within *Bacteroides* or Bacteroidetes. To be accurate but more specific as suggested by the reviewer, we have modified our description by mentioning the Bacteroidetes phylum but also specifying *Bacteroides* and *Prevotella* species.
- In the Results, we removed description about previous findings to reduce redundancy (since this is discussed in the Discussion section). We modified the Results (now in lines 237-238) from:

“We confirmed that these genes are mostly found in MAGs within Bacteroidetes (Fig. 5b), consistent with the higher abundance of Bacteroidetes in the industrial samples as detected by MetaPhlAn2 (Fig. 2) and with previous findings that Bacteroidetes species possess many glycandegrading genes[64,65].”
to:
“These genes are mostly found in SGBs within Bacteroidetes, including *Bacteroides* and *Prevotella* species (Supplementary Information section 14).”
- Meanwhile, we modified the Discussion (now in lines 260-265) from:
“The industrial samples are also enriched in mucin-degrading genes (Figs. 5a and 5c), which are mostly from *Bacteroides* species that are highly abundant in those samples (Fig. 5b, Fig. 2d). This

is consistent with previous studies demonstrating enrichment of mucin-utilizing enzymes in the industrialized gut microbiome[1] and the mucin-degrading capacity of many *Bacteroides* species[65]."
to:
"Further, the industrial samples are enriched in mucin-degrading genes (Fig. 3) found in *Bacteroides* and *Prevotella* SGBs (Supplementary Information section 14). This is in line with the higher abundance of Bacteroidetes in the industrial samples (Fig. 1), previous findings that members of the Bacteroidetes phylum possess many glycan-degrading genes[37,38], and the enrichment of mucin-utilizing enzymes in the industrialized gut microbiome[1,38]."

Comment: Lines 1489-1490: Calling something "prebiotic" just because it increases the number of Bifidobacteria, especially in such as varied diet as the people at Zape had, is kind of a miss in my book. Many things they ate could probably be called "prebiotic" by industrial standards. Perhaps replace "prebiotic" by "bifidogenic"? Or, "… would have a bifidogenic, and by todays standards, prebiotic effect on the gut microbiome."
Response: We agree with the reviewer that "bifidogenic" is more appropriate to use here. We have replaced "prebiotic" with "bifidogenic" (now in lines 1418-1419).

Comment: Line 1498: Again, perhaps replace prebiotic with "bifidogenic" or "bifidogenic/prebiotic"?
Response: We have replaced "prebiotic" to "bifidogenic" here as well (now line 1427).

Comment: Possible minor errors (though I might be wrong): Line 397: "is" attainable?
Response: We believe "are" is correct because we are referring to "*de novo* assembly" and "recovery of novel microbes from paleofeces" (now line 278).

Line 398: remove "the"?
Response: This sentence has been removed as an attempt to reduce the length of the manuscript.

Line 410: add "the" in front of absence?
Response: We have incorporated this into the text (now line 290).

Referee #2 (Remarks to the Author):
Overall, I'm satisfied with the authors responses to my comments. I have a few additional minor comments / suggestions for the authors consideration (revised ms line numbers):

Comment: Line 199: The species level analysis of enriched taxa in the paleofaeces made me remember Bifidobacterium longum subsp. infantis, a species thought to be important for the normal development of the infant gut microbiome which has become "extinct" in the modern westernised gut – see https://www.ncbi.nlm.nih.gov/pmc/articles/PMC7352178/
Was this species present in the paleofaeces?
Response: *Bifidobacterium longum* subspecies *infantis* (*Bifidobacterium infantis*) was not identified by MetaPhlAn2 in any of the samples. However, MetaPhlAn2 identified *Bifidobacterium longum,* which is present in 5 out of the 8 paleofeces, 148 out of 370 non-industrial samples, and 325 out of 421 industrial samples (Supplementary Table 3). One MAG was also classified as *Bifidobacterium longum* (Lib4_3_bin.22), but none was classified as *Bifidobacterium longum* subspecies *infantis* specifically (Supplementary Table 6).

Comment: Lines 349-352: Are these antibiotic resistance genes chromosomally encoded? Plasmid borne AbR genes are notoriously difficult to associate to their hosts via MAGs, so this caveat should be noted in this analysis. Are there other AbR genes in the metagenomes not associated with MAGs pointing to potential plasmid borne genes? And are such untethered AbR genes mostly absent in the paleofaeces?
Response: We thank the reviewer for this feedback. The tetracycline resistance genes described in

lines 349-352 (now lines 229-233) are:
- tetracycline resistance ribosomal protection protein Tet(O)
- tetracycline resistance ribosomal protection protein Tet(M)
- tetracycline resistance ribosomal protection protein Tet(Q)
- Tetracycline resistance protein TetO

We performed the suggested analysis and described the results in lines 233-234: “Platon[34] analysis suggests that these tetracycline resistance genes are encoded chromosomally in the MAGs, rather than on plasmids (Supplementary Information section 13).”

We also described the methods and results in more detail in Supplementary Information section 13 (pages 112-113) and Supplementary Figure 18:
“To determine whether the antibiotic resistance (AbR) genes are on plasmids or chromosomes, we first mapped which MAGs and contigs contain the AbR genes of interest. PROKKA (version 1.14.6)[46] was run for all of the MQ/HQ filtered ancient MAGs, the MQ/HQ Mexican MAGs, and 1,451 representative MAGs from Pasolli et al.[14] that originated from the metagenomes used in our study. Platon[34] was run for the MAGs that possess those AbR genes to predict whether the contigs containing those AbR genes are on chromosomes or plasmids. According to Platon, none of these genes are on plasmids in the MAGs.
As shown in Fig. 3a, Supplementary Table 8, and Supplementary Fig. 18, there are additional AbR genes in the metagenomes that are not associated with MAGs. These AbR genes might be plasmid borne. Some of these AbR genes are prevalent in the present-day samples but are mostly absent in the paleofeces. These include most of the tetracycline resistance genes, extended-spectrum betalactamase PER-1, lincosamide resistance protein, and antibiotic efflux pump periplasmic linker protein ArpA. However, there are also antibiotic resistance genes that are more abundant in the paleofeces compared to the present-day samples, such as fosfomycin resistance protein FosX and Daunorubicin/doxorubicin resistance ABC transporter permease protein DrrB (Supplementary Table 8, Supplementary Fig. 18).”

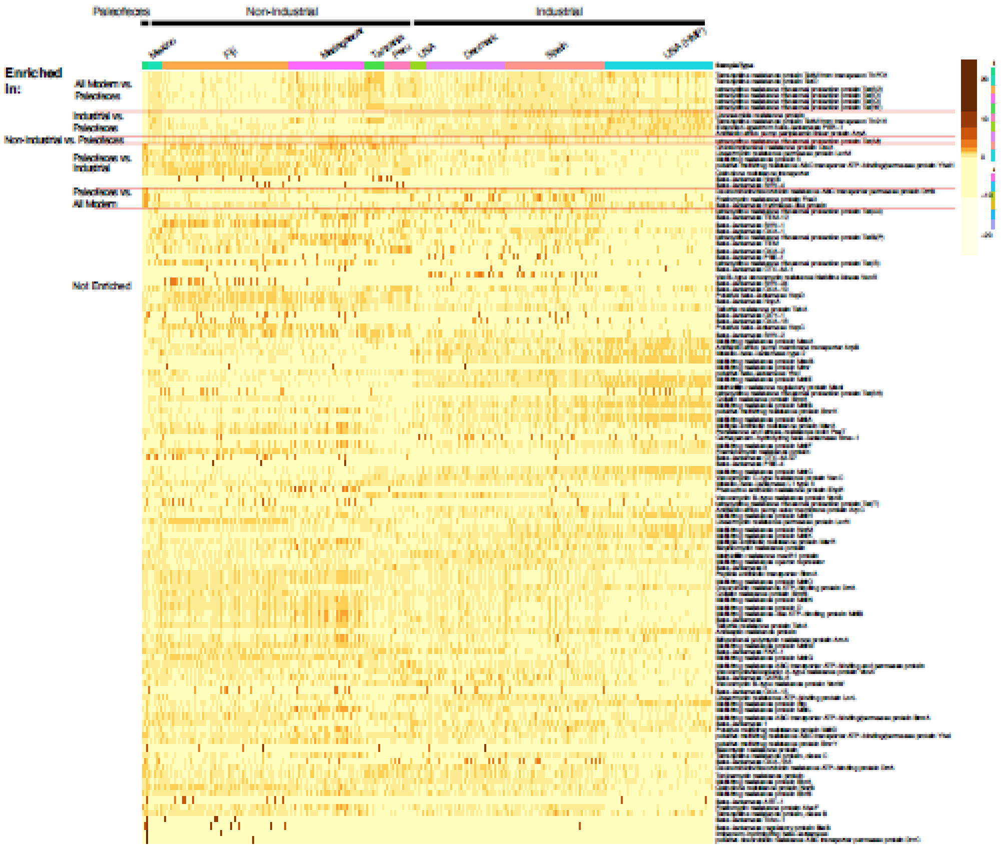

Supplementary Fig. 18. Heatmap of 120 antibiotic resistance genes found in the paleofeces, industrial, and non-industrial samples (related to Fig. 3). Functions were annotated using PROKKA[46] with UniProtKB database. Enriched genes were identified with one-tailed Wilcoxon tests with Bonferroni correction. Non-enriched genes were sorted by fold change. RPKM values are shown on a log scale and scaled by row.

Comment: Lines 529-530, ref 50: This preprint is now published:
https://www.nature.com/articles/s41587-020-0603-3
Response: We thank the reviewer for pointing this out and we have now updated this citation.

Comment: Line 1066: What alignment fraction (AF) was used with fastANI? This is the other important parameter setting for ANI calculations and it should be mentioned somewhere.
Response: We used dRep's default alignment fraction (AF), which is 0.1 (the "-nc'' parameter). We have added this detail in lines 899-900: "The dRep[87] "cluster" command was utilized to run FastANI[88] using default alignment fraction (0.1) and with the following settings: -sa 0.95 --S_algorithm fastANI."

Comment: Line 1519: I thank the authors for performing the %read mapping analysis against the different faecal samples showing the expected relative mapping, however, why is there such a low % of reads (<4%) mapping to the MetaPhlan2 database? Typically 50-90% of faecal shotgun reads map to reference genome databases. Presumably this is a function of the database itself and because MetaPhlan2 doesn't map to whole genomes, but this should be explained here (even so, this % read mapping seems remarkably low).

Response: We have now added this explanation in the Supplementary Information section 5 (page 82): "The percentage of reads aligned to MetaPhlAn2 database per sample is not high, but this was expected. MetaPhlAn2 is based on an average of 184 (⍰} 45) marker genes for each bacterial species[23].
Since an average bacterial genome contains 5,000 genes[134,135], MetaPhlAn2 database would cover about 3.68% of a given bacterial genome. Considering that many microbial species (77%) are still unknown/have never been described before (as shown in Pasolli et al.[14]) and that MetaPhlAn2 does not have the "unknown" species in the database, the ~3% alignment rate for HMP samples is expected. For non-industrial samples (Fijian and Mexican), we expect an even lower rate of alignment because they are composed of more "unknown" species that are not present in the MetaPhlAn2 database. For soil samples, the alignment rate is ~10 times lower because there are probably ~10 times more species that are not in the database, since the diversity of soil microbiome is 10x higher than that of the gut microbiome[136]."

Referee #3 (Remarks to the Author):
The authors have conducted a nice revision, addressing our major points related to read-mapping and relative abundance methods, as well as with calculating and reporting species novelty.
New aspects of functional analysis

Comment: • In the heat-map there appear to be relative abundance values at 1 - were these values scaled at all prior to plotting? If so, please include that in the methods section.
Response: We have now converted the relative abundance values to RPKM and on a log scale. We show the values scaled by row in Fig. 3a and without scaling in Supplementary Fig. 11. We have added these details in the figure legends in lines 632-633 (Fig. 3a): "RPKM values shown are on a log scale and scaled by row. Heatmap with unscaled values are shown in Supplementary Information section 11." and line 1749 (Supplementary Fig. 11): "RPKM values are shown on a log scale."

Comment: • The methods section states that gene relative abundance was calculated in a way similar to RPKM (reads per kilobase million), but potentially without the same units. Because RPKM is a common measure in bioinformatics literature, it would be beneficial to calculate relative abundance in terms of RPKM and report the values as such (have the heatmap show actual RPKM values on a log scale, not unitless scaled values).
Response: As suggested by the referee, we have now converted our calculation to RPKM. We have added this detail in the Methods (lines 993-995): "RPKM values were calculated by multiplying the relative abundance values by 1,000 (for the per kilobase conversion) and 1,000,000 (for the per million conversion)." We show the RPKM values on a log scale after scaling by row in Fig. 3a and without scaling in Supplementary Fig. 11. The boxplots in Supplementary Information section 12 show the RPKM values on a linear scale (pages 106-111).

Comment: • The analysis in Figure 5b is very confusing. Is each column an organism, or a taxa? It appears that some organisms are being counted multiple times at different taxonomic levels (e.g. in the figure on the left, the species and genus of Prevotella all have the same count). Please either remove this sub panel (which would be fine- the figure is already large without it) or add significantly more explanatory text to the figure caption / results / methods.
Response: We have now moved Fig. 5b to Supplementary Information section 14. We have added more explanation about the plots in page 114: "To investigate which taxa in the samples are carrying the genes of interest, we checked for the presence of these genes in the MAGs. First, to map which MAGs possess the enriched genes, PROKKA (version 1.14.6)[46] was run for a total of 1,938 bins, which included all of the representative ancient bins, the representative Mexican bins, and the representative bins from Pasolli et al.[14] that originated from the industrial and non-industrial samples used here. A binary matrix was created with the bin names as columns and the genes as rows. Significantly enriched genes from the functional analysis were searched in the binary matrix. Second, to assign taxonomies to the bins, for the ancient and Mexican bins, the bins were annotated with the lowest taxonomic rank assigned by GTDB-Tk[26]. For the bins from Pasolli et al.[14], taxonomic names were assigned based on the lowest taxonomic rank of their "estimated taxonomy". Finally, for each sample type (paleofeces/nonindustrial/industrial), we plotted the number of bins within each taxonomic annotation that contain those specific genes (Supplementary Figs. 19 and 20). For instance, for the paleofeces, SusD-like protein P2 is found in two bins annotated as g__Prevotella. In the non-industrial samples, SusD-like protein P2 is found in 14 bins annotated as g__Prevotella." The x-axis shows the lowest taxonomic annotation of the SGBs. Some organisms are shown multiple times at different taxonomic levels because the associated MAGs were assigned to different taxonomic levels according to GTDB-Tk, as explained above. For instance, for the paleofeces, SusD-like protein P2 is found in two bins annotated as g__Prevotella and two other bins annotated as s__Prevotella_sp.

Comment: • The enrichment of transposases is super interesting. Would it be possible to determine if any genes are enriched near these transposases?
Response: We thank the referee for this suggestion. We have now identified genes near these transposases and we describe the analysis in Supplementary Information section 10 (pages 101-104): "To identify what genes frequently surround the transposases, for all of the samples, we identified contigs that contain "transposase" or "Transposase" from the PROKKA output files (.gff files). We took genes that are from those same contigs and are within 1000bp (average gene length) from the transposases, and counted how many times each gene shows up per sample type (paleofeces/nonindustrialized/industrialized). From the results, we removed genes with incomplete names (e.g. "n", "in", "ein", '5", "tein", and "0") and genes labeled as "hypothetical protein" or "transposase" (because the results consisted of an overwhelmingly large number of unique transposases). The top 50 genes surrounding transposases per sample type are shown below in Supplementary Fig. 10. We annotated the top genes with functions according to UniProtKB[140] and

found that many of these genes are recombinases, DNA repair proteins, stress response proteins, and pyrimidine biosynthesis enzymes (Table S1)."
The results are shown in Supplementary Fig. 10 (also attached below), Table S1 (also attached below), and described in lines 223-224 in the Results section: "Genes surrounding these transposases are mostly recombinases, DNA repair proteins, stress response proteins, and pyrimidine biosynthesis enzymes (Supplementary Information section 10)."

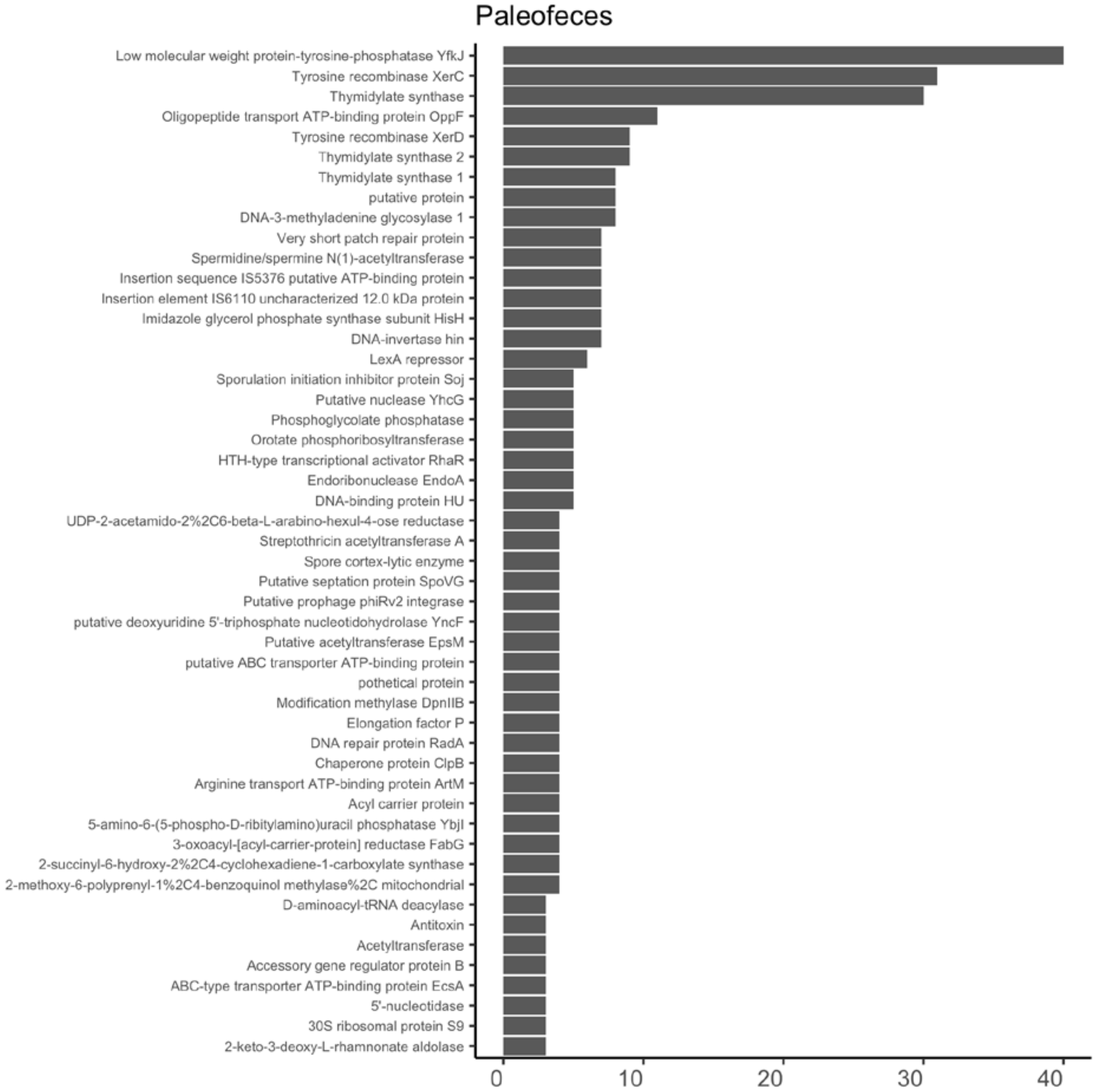

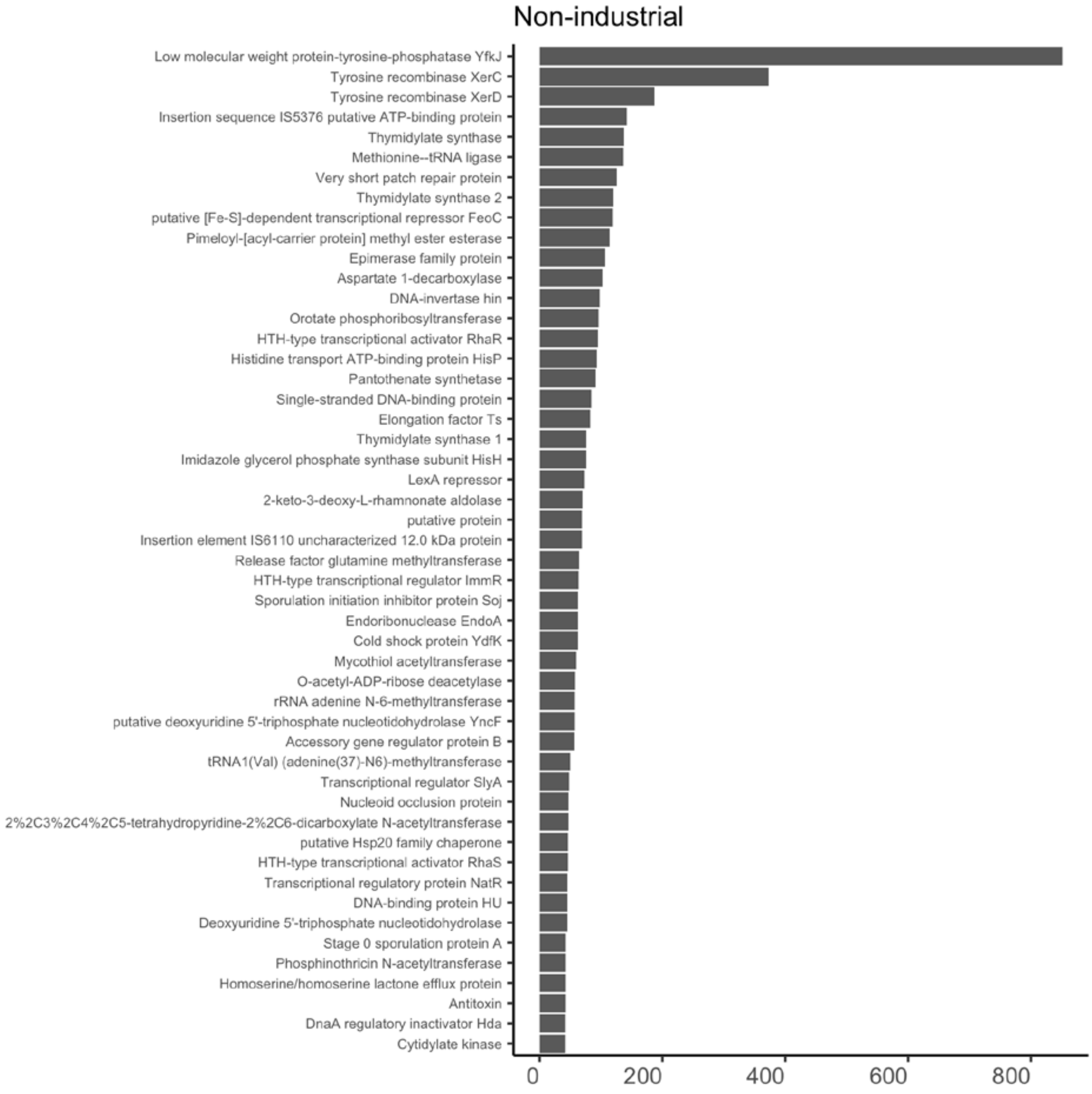

Non-industrial
Low molecular weight protein-tyrosine-phosphatase YfkJ
Tyrosine recombinase XerC
Tyrosine recombinase XerD
Insertion sequence IS5376 putative ATP-binding protein
Thymidylate synthase
Methionine--tRNA ligase
Very short patch repair protein
Thymidylate synthase 2
putative [Fe-S]-dependent transcriptional repressor FeoC
Pimeloyl-[acyl-carrier protein] methyl ester esterase
Epimerase family protein
Aspartate 1-decarboxylase
DNA-invertase hin
Orotate phosphoribosyltransferase
HTH-type transcriptional activator RhaR
Histidine transport ATP-binding protein HisP
Pantothenate synthetase
Single-stranded DNA-binding protein
Elongation factor Ts
Thymidylate synthase 1
Imidazole glycerol phosphate synthase subunit HisH
LexA repressor
2-keto-3-deoxy-L-rhamnonate aldolase
putative protein
Insertion element IS6110 uncharacterized 12.0 kDa protein
Release factor glutamine methyltransferase
HTH-type transcriptional regulator ImmR
Sporulation initiation inhibitor protein Soj
Endoribonuclease EndoA
Cold shock protein YdfK
Mycothiol acetyltransferase
O-acetyl-ADP-ribose deacetylase
rRNA adenine N-6-methyltransferase
putative deoxyuridine 5'-triphosphate nucleotidohydrolase YncF
Accessory gene regulator protein B
tRNA1(Val) (adenine(37)-N6)-methyltransferase
Transcriptional regulator SlyA
Nucleoid occlusion protein
2%2C3%2C4%2C5-tetrahydropyridine-2%2C6-dicarboxylate N-acetyltransferase
putative Hsp20 family chaperone
HTH-type transcriptional activator RhaS
Transcriptional regulatory protein NatR
DNA-binding protein HU
Deoxyuridine 5'-triphosphate nucleotidohydrolase
Stage 0 sporulation protein A
Phosphinothricin N-acetyltransferase
Homoserine/homoserine lactone efflux protein
Antitoxin
DnaA regulatory inactivator Hda
Cytidylate kinase
0
200
400
600
800

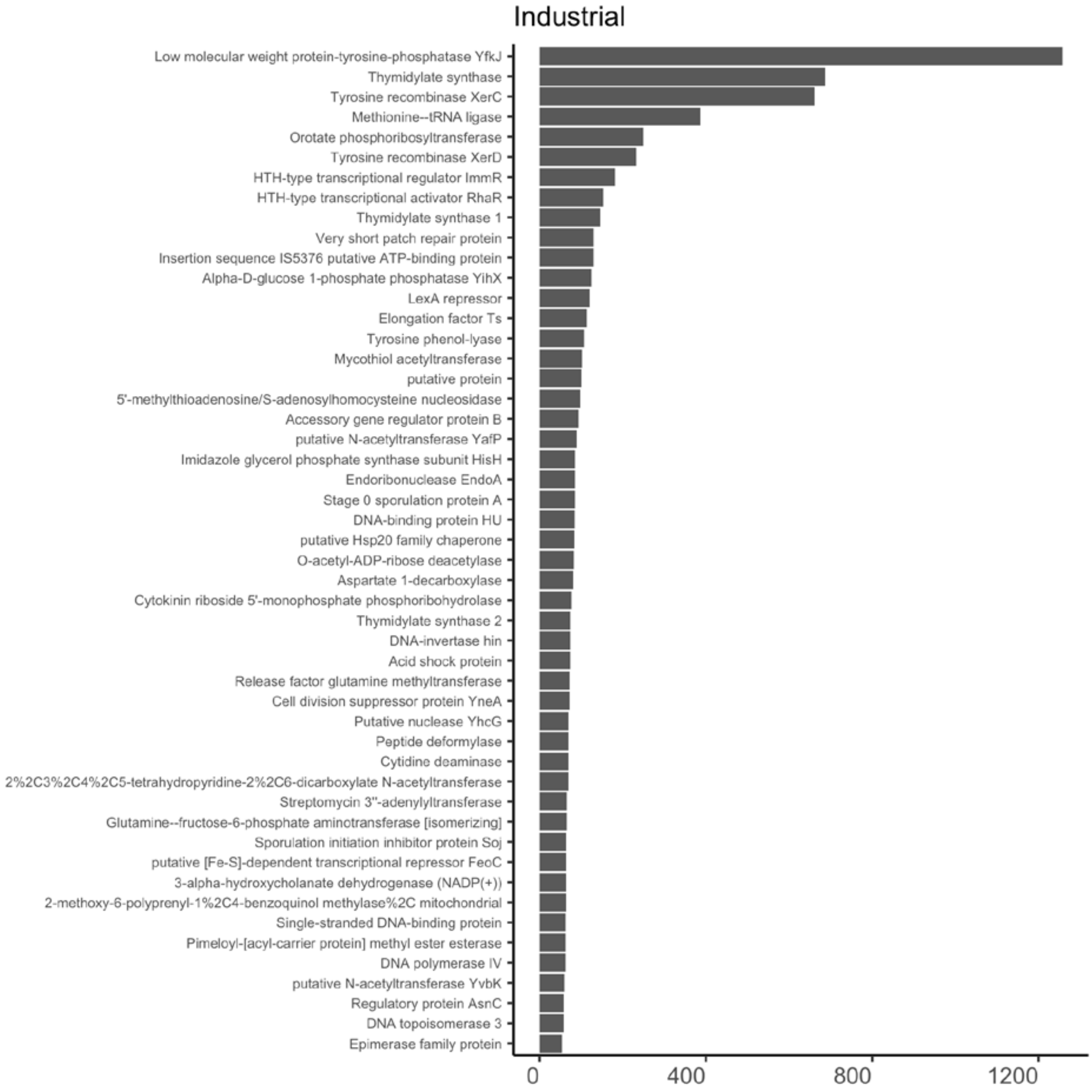

Supplementary Fig. 10. Top 50 genes found within 1000bp of transposases in the paleofeces, non-industrial, and industrial samples (related to Fig. 3).

Table S1. Genes surrounding transposases that are within the top 50 for all three sample types (paleofeces, non-industrial, industrial).

Gene	Function (according to UniProtKB)
Low molecular weight protein-tyrosine-phosphatase YfkJ	Involved in ethanol stress resistance
Tyrosine recombinase XerC	Site-specific tyrosine recombinase
Thymidylate synthase	Pyrimidine biosynthesis
Thymidylate synthase 2	Pyrimidine biosynthesis
Tyrosine recombinase XerD	Site-specific tyrosine recombinase
putative protein	-
Thymidylate synthase 1	Pyrimidine biosynthesis
DNA-invertase hin	Serine recombinase family of DNA invertases
Imidazole glycerol phosphate synthase subunit HisH	L-histidine biosynthesis (amino acid biosynthesis)
Insertion sequence IS5376 putative ATP-binding protein	ATP binding
Very short patch repair protein	DNA repair
LexA repressor	DNA repair
DNA-binding protein HU	Histone-like DNA-binding protein to prevent DNA denaturation under extreme environmental conditions.
Endoribonuclease EndoA	Toxin - stress response
HTH-type transcriptional activator RhaR	Regulation of transcription
Orotate phosphoribosyltransferase	Pyrimidine biosynthesis
Sporulation initiation inhibitor protein Soj	Inhibits the initiation of sporulation
Accessory gene regulator protein B	Pathogenesis, quorum sensing

Comment: Is anything known about the particular transposase families that were recovered?
Response: Many diverse families of transposases were found to be enriched, so it is difficult to pinpoint the specific functions of these diverse families of transposases.

Comment: The authors present a new analysis of M. smithii MAGs. Not sure this analysis is appropriate or required for this paper.
Response: We performed this analysis in response to the request of another reviewer. To address this comment, we provide some clarifications and have inserted some related clarifying text in the article, as described below.

Comment: Specifically, the authors should clarify (i) how the ancient genomes change what the analysis would have yielded otherwise
Response: This analysis indicates that *de novo* reconstructed ancient microbial genomes can be used in evolutionary studies. The age of the paleofeces serves as a calibration point to set the clock and infer the divergence of the whole *M. smithii* radiation. In the absence of a specific prior mutation rate, the ancient genomes are necessary for estimating the divergences of *M. smithii*. This is described in lines 210-213: "Overall, we show that using ancient genomes for calibrating *M. smithii* phylogenies, we could evolutionarily match previous studies on *M. oralis*[27]. This supports the potential of utilizing ancient genomes reconstructed from paleofeces to study the evolutionary history of the gut symbionts."

Comment: (ii) any interesting implications of this analysis for the ancient genomes, M. smithii, human migrations, microbial evolution, etc.
Response: We now describe this in the Results (lines 201-206):
"The two estimates are compatible in terms of HPD overlap, and both occurred within or slightly after the estimated first human migration waves out of Africa ca. 90,000 - 194,000 years ago[28,29]. In addition, the origin of the lineage leading to the two ancient *M. smitihii* genomes are set between 40,000 and 16,000 years ago (mean = 27,000 years ago). These estimates predate (although there is

a certain overlap toward the earlier 95% posterior estimates) the accepted age of human entry to North America via the Beringia bridge (20,000 - 16,000 years ago)."
This is also described in Supplementary Information section 9 (lines 1681-1685): "The inferred divergences indicate that *M. smithii* radiated in a timeframe compatible with the major human migrations. The origin of the lineage leading to the two ancient *M. smitihii* genomes are set between 40,000 and 16,000 years ago (mean = 27,000 years ago). These estimates predate (although there is a certain overlap toward the earlier 95% posterior estimates) the accepted age of human entry to North America via the Beringia bridge (20,000 - 16,000 years ago)."

Comment: The results presented seem to require a bit of clarification:
L..297-99 "and that M. smithii began to diversify ~85,000 years ago with a 95% highest posterior density (HPD) interval 51,000 ~ 128,000 years (Fig. 4c). This timeline is moderately earlier than the one of its sister species M. oralis (HPD: 112.000 - 143.000 years)"
- Don't the authors mean later rather than earlier? (after)

Response: We apologize for this mistake. We now have corrected the sentence (lines 200-201) to: "This timeline is moderately later than the one of its sister species *M. oralis* (HPD: 112,000 - 143,000 years)[27]."

Comment: - How do ancient strains, which may be intermediates of others fit into a tree like this, where divergence is implied?
Response: The ancient strains are not intermediates, but rather belong to an old genetic variant (circa 27,000 years) which has been likely lost in other parts of the world. This is suggested by the long uninterrupted branch leading to the two ancient strains.

Referee #4 (Remarks to the Author):
Comment: By carrying out comparisons between the microbial composition of sediment samples from the study site and those of the coprolites I think the authors have done what is currently possible addressing my concern regarding environmental contamination of the past. One thing I think is worth reporting is a comparison of the damage and length distribution of the ancient soil microbes and the ancient microbiome from the same site (can't see the authors have done this). These are expected to be fairly similar and if this is indeed the case it would put some strength to their argumentation that they can indeed use the comparative approach for separating the two fractions and that the soil microbes they compare are indeed ancient.
Response: To address this comment, we analyzed the damage patterns of the three soil samples from Boomerang Shelter by running DamageProfiler using reconstructed microbial genomes from the samples as reference (1st row in the figure below). The results show damage patterns similar to the paleofeces (2nd, 3rd, and 4th row in the figure below). This is consistent with the reviewer's expectation. These results are now also shown in Extended Data Fig. 3.

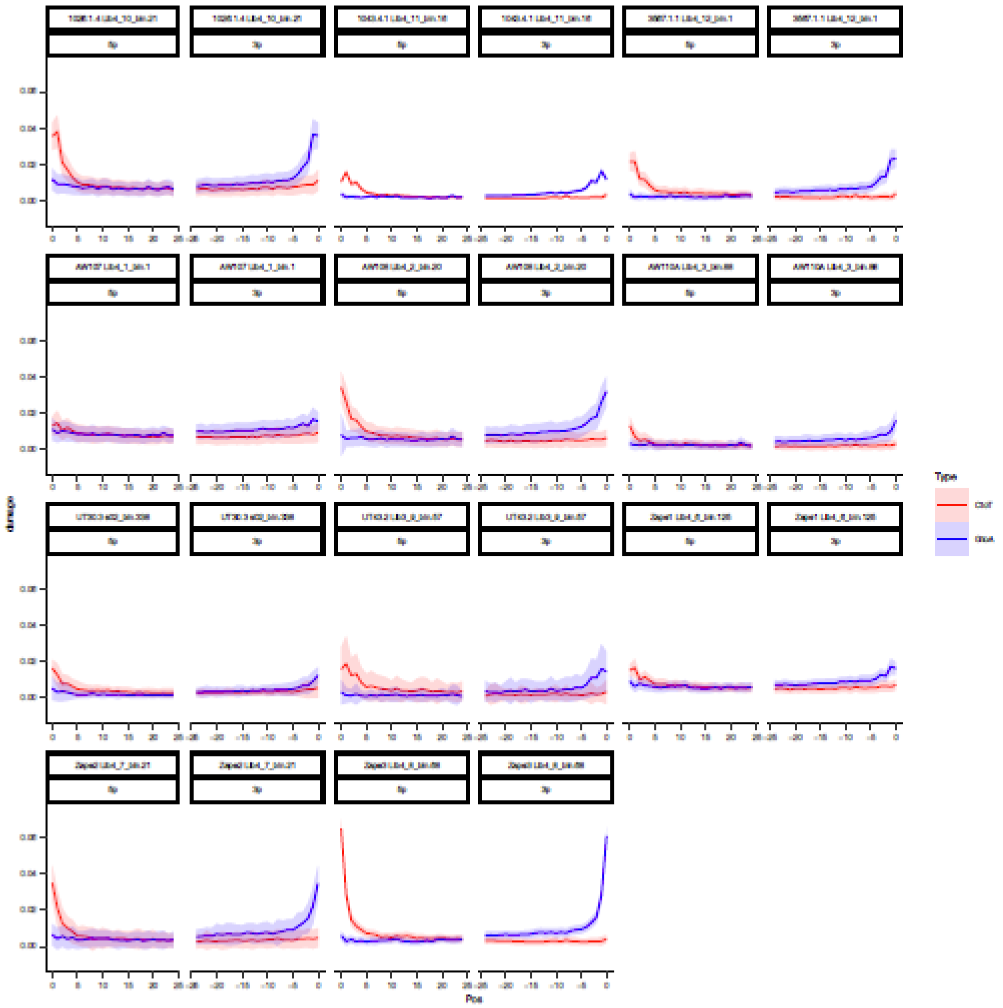

Extended Data Fig. 3. DNA damage patterns of the paleofeces and the Boomerang soil samples as identified by DamageProfiler[93]. The first row shows the Boomerang soil samples, while the other rows are the paleofeces. All bins used as reference genomes for the paleofeces are of known gut microbial species. Red line indicates the average frequency of C to T substitution across all contigs per bin, and the blue line indicates the average frequency of G to A substitution across all contigs per bin. The shaded areas show standard deviation. Contigs with <1,000 reads aligned were removed from the analysis.

For length distribution, we do not have data regarding fragment length distribution per sample for all samples, hence it is challenging to compare the distribution accurately. We only have fragment length distribution data per library, and each library contains between one to three samples. For two of the soil samples (1026.1.4 and 3567.1.1), BioAnalyzer was run together with one paleofeces (AW116), as shown below.

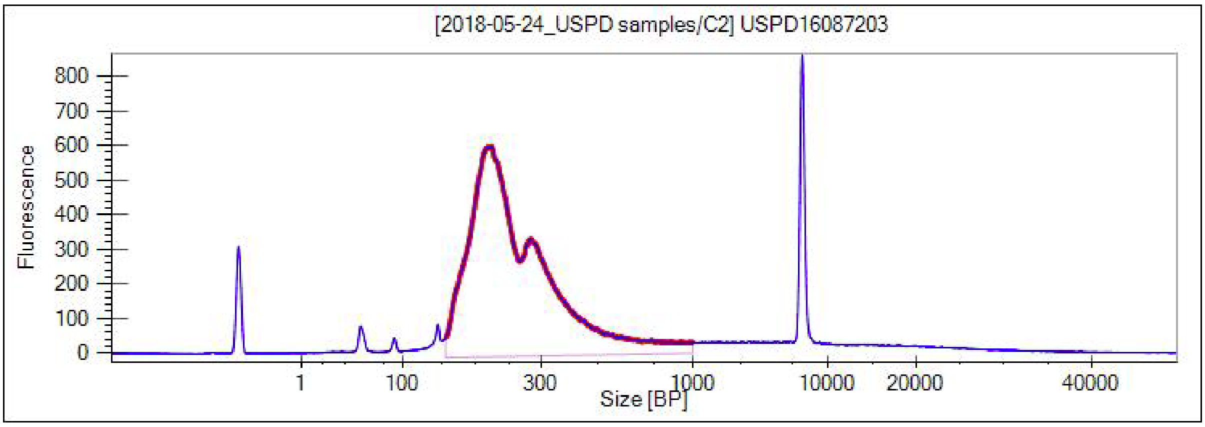

The other soil sample (1043.4.1) was run together with two paleofeces (AW113 and TS902), as
shown
below.

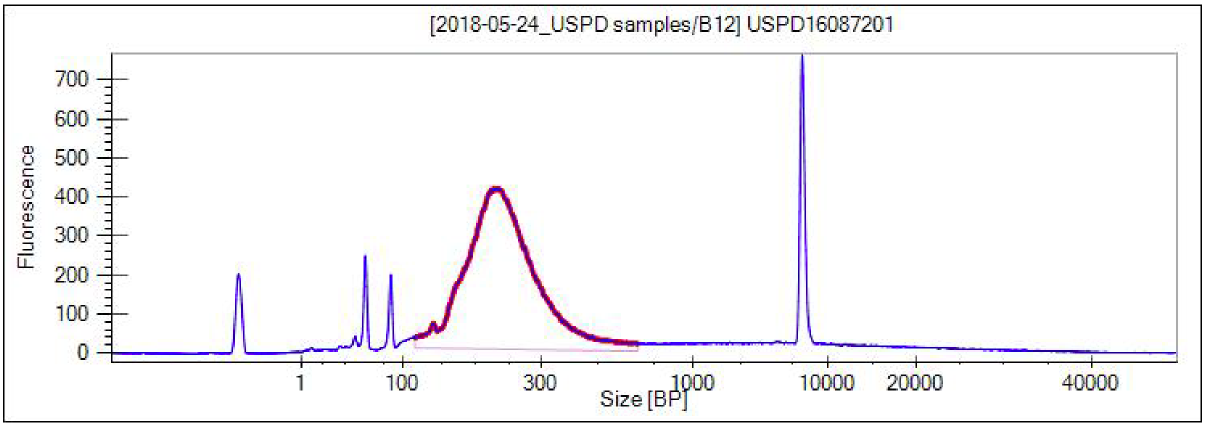

Meanwhile, below is the fragment length distribution for the paleofeces:

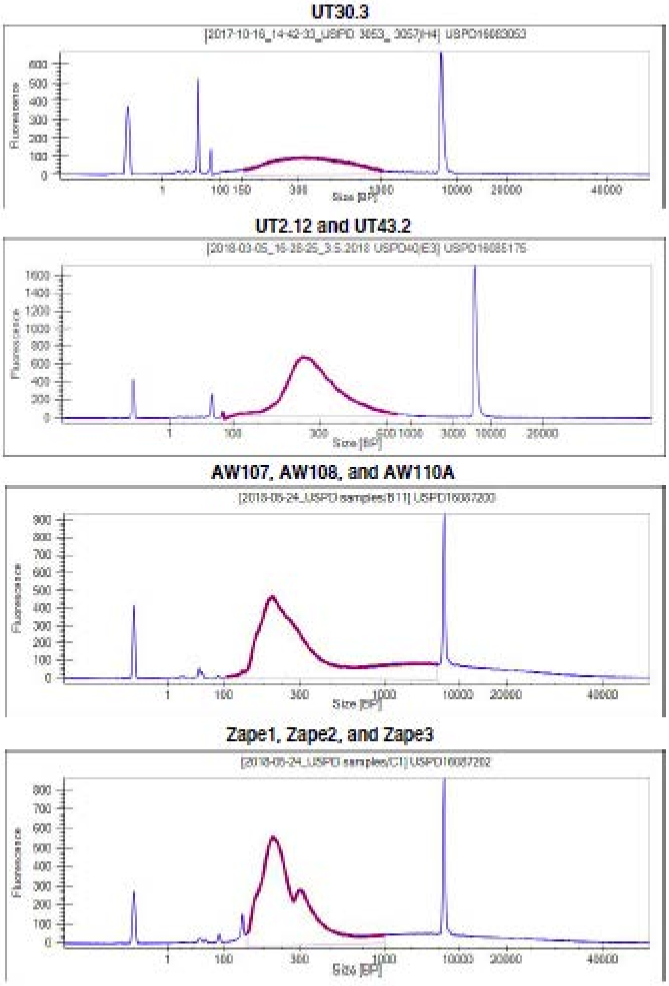

Overall, fragment length distribution appears to be similar between the soil samples and the paleofeces. All of these plots are now shown in Extended Data Fig. 6 (page 60).

Referee #5 (Remarks to the Author):
In this study, Wibowo and colleagues reconstruct a collection of ancient microbial genomes from several ~1000yr-old human paleofeces samples. In this review, I focused only on the SNP and molecular clocking analyses that the authors have added in their revision. I think these are largely in the right direction, though I have a few technical suggestions for improvement:

Comment: (1) Since the two ancient M smithii genomes were obtained from paleofeces from the same site, it seems like it would be difficult to exclude the possibility that they come from the same person --- or at the very least, from individuals that share a common household. As such, I think it is important to allow for the possibility that the two M smithii genomes share a recent common ancestor for epidemiological (rather than evolutionary) reasons. In this light, the SNP analysis on p.92 is still very informative for ruling out DNA damage artifacts (which should occur independently in the two samples), but I don't think it supports the conclusion that the ancient samples are particularly distant from the modern strains (at least, not any more than the modern strains are from each other), since the authors' control does not account for the close relatedness of the ancient samples. I think the basic point could be made in a more robust way by simply re-running some the same phylogenetic analyses (see point 2 below) using the inferred common ancestor of the ancient strains (i.e., SNPs that are shared by both ancient strains, but not necessarily absent in the modern strains). **Response:** We thank the reviewer for this detailed feedback. These two paleofeces must have been deposited by different individuals because the haplogroups inferred

from these two samples are different (Supplementary Table 1). However, it is possible that those individuals shared a common household. Therefore, as the reviewer suggested, we validated our analysis reported in the manuscript by re-running the phylogenetic analyses described below using the inferred common ancestor of the ancient strains, as elaborated in point (2).
Due to the possible relatedness of the two ancient *M. smithii* genomes, we also decided to remove our SNP analysis (previously on page 92). We believe the analysis suggested by the reviewer, together with our BEAST2 analysis after trimming the first and last 5 base pairs of the reads (page 46, Supplementary Fig. 8b), are enough to rule out the effects of DNA damage artifacts.

Comment: (2) One could argue that the phylogenetic model used for the divergence estimates is not quite appropriate for recombining populations of bacteria (see, e.g. Vaughn et al Genetics 2017), though it is an understandable choice to facilitate comparisons with previous divergence estimates from Weyrich et al (Nature, 2017). I think this section could be improved by supplementing the existing BEAST analysis with a more lightweight approach, which simply reports the raw sequence divergence between the different pairs of strains.
Given the results in Fig. 4C, it would be informative to split the M smithii strains into 3 groups: the two ancient genomes (A), the modern genomes plotted above the ancient genomes in Fig. 4C (M1), and the modern genomes plotted below the ancient genomes (M2).
Response: We acknowledge the reviewer's technical suggestion and performed the analysis as suggested. As described in lines 1598-1608 and shown in Supplementary Fig. 9: "To validate our inferred dates in Fig. 2d, we re-calibrated divergence dates between A & M1 *Methanobrevibacter smithii* strains (Supplementary Fig. 9a) by calculating raw genetic distances. We divided all strains into three groups: M1(n=24), A (n=2), and M2 (n=4) (Supplementary Fig. 9a). To measure raw sequence divergences between A & M2 strains, M1 & M2 strains, and A & M1 strains, we calculated single nucleotide variant (SNV) rates (the number of variant sites divided by the total number of sites considering only those not containing gaps, using the same reconstructed alignment used in our reported BEAST2 analysis) for each pair of strains and plotted the pairwise divergences for all group combinations (Supplementary Fig. 9b). The results show that sequence divergences (SNV/site) between M1 & M2 strains range from 0.0489 to 0.0546 with a mean of 0.0523; those between A & M1 strains range from 0.0187 to 0.0224 with a mean of 0.0205; those between A & M2 strains range from 0.0495 to 0.0514 with a mean of 0.0507."

Comment: One could then plot all sequence divergences between A & M2 strains, M1 & M2 strains, etc. In the strict clock model, the phylogenetic signal used for the dating would then show up as a systematic difference between the d(A,M2) and d(M1,M2) divergences: d(M1,M2) - d(A,M2) ~ (substitutions per year * ~1000 yrs) which calibrates the remaining genetic distances in units of years. The results would ideally be consistent with the dates inferred in Fig. 4C, while any large discrepancies from the BEAST model would immediately jump out.
Response: We agree with the reviewer that re-running the calibration based on a systematic difference between M1 & M2 and A & M2 divergences (d(M1, M2) - d(A, M2)) would be a robust way to validate our inferred dates in Fig. 2d (previously Fig. 4c) under the assumption of a strict clock model. Thus, we re-calibrated the genetic distance between A & M1 strains using systematic differences as phylogenetic signals, and compared the results with our BEAST2 estimates.
First of all, to avoid biases from uneven missing information in different pairs when calculating raw sequence divergences, we performed the analysis using SNV rates (the number of SNVs divided by the number of sites, when only considering sites without gaps). Thus, the formula "d(M1, M2) - d(A, M2) ~ (substitutions per year * ~ 1000 yrs)" was transformed into d(M1, M2) - d(A, M2) ~ (substitutions per site per year * ~ 1000 yrs). For accuracy, we also formalized "~1000 yrs" by using the real age of the two ancient samples, which is 1985 years old (by averaging C14 mean corrected dates of samples UT30.3 and UT43.2, Fig. 1c). Therefore, we carried out the analysis using the formula: d(M1, M2) - d(A, M2) ~ (substitutions per site per year * 1985 yrs). As described in the

manuscript in pages 95-98 and shown in Supplementary Fig. 9: “To calculate systematic differences in substitutions per site per year, we used the formula d(M1, M2) - d(A, M2) ~ (substitutions per site per year * 1985 years), in which 1985 is the C14 mean corrected dates of samples UT30.3 and UT43.2 (Extended Data Fig. 1c, Supplementary Table 1). We started with a rough calibration by calculating d(M1, M2) - d(A, M2) using the average of d(M1, M2) and the average of d(A, M2): 0.0523 - 0.0507 = 0.0016. Based on the formula d(M1, M2) - d(A, M2) ~ (substitutions per site per year * 1985 yrs), we obtained substitutions/site/year = 0.0016/1985 = 8.060453e-07. Thus, the divergence date between A and M1 based on the average sequence divergences can be obtained by: the average of d(A, M1) divided by 8.060453e-07 → 0.0205/8.060453e-07 = 25432.81 years (25.43 thousand years ago), which is quite close with the reported divergence date for A & M1 from our BEAST2 analysis (27.58 thousand years ago).”

Further, we performed the analysis in more detail by using all pairwise sequence divergence values instead of just the averaged values. In detail:

First, we calculated all pairwise systematic differences between M1 & M2 (N = 96) and A & M2 (N = 8) strain divergences, resulting in 768 pairwise values of d(M1, M2) - d(A, M2) (Supplementary Fig. 9c).

Those values range from -0.0025 to 0.0051, with a mean of 0.0016 (negative values indicate the few instances when M1 & M2 divergences are smaller than A & M2 divergences). As a comparison, we calculated systematic differences based on raw sequences (d(M1, M2) - d(A, M2)) with accumulated SNV rates (evolutionary rate * evolutionary time, which equates the product of inferred clock rate and the age of the common ancestor of two ancient strains) based on our BEAST2 analysis. This was performed by calculating the product of rate estimates from all iterations of simulation (Supplementary Table 7) and the inferred age of the common ancestor of the ancient strains (2198 BP, consensus tree file attached). The resulting values range from 0.0004 to 0.0025 with a mean of 0.0014 (Supplementary Fig. 9d). The distribution of results calculated based on the BEAST2 analysis falls well within the range of systematic differences calculated using raw sequence divergences, with a very similar mean estimate as well. These results indicate a preliminary consensus between the raw sequence divergence calculation and the inferred estimates from the BEAST2 analysis.

Second, we converted pairwise systematic differences (substitution/site) to pairwise time-resolved systematic differences (substitution/site/year), which could be used for calibrating the remaining genetic distances, by dividing systematic differences with the average C14 age (1985 years old) of the two paleofeces (Supplementary Fig. 9e). In addition, we plotted the distribution of clock rate estimates (which are the signals in molecular clocking analysis) from the existing BEAST2 results (Supplementary Fig. 9f, Supplementary Table 7). As seen below, the distribution of our existing clock rate estimates is restricted in a narrow uncertainty range and falls within the range of time-resolved systematic differences based on raw sequence divergences (Supplementary Fig. 9e, f).

Third, we re-calibrated the genetic distances between A & M1 using the time-resolved systematic differences (d(A, M1)/time-resolved systematic differences, Supplementary Fig. 9g), which should give a result consistent with the estimated dates of the node leading to the divergence between A and M1 strains in Fig. 2d. To compare the re-calibrated dates with our divergence date estimates, we re-plotted the distribution of all estimated A & M1 divergence dates from our BEAST2 analysis (Supplementary Fig. 9i, which has been shown as the orange violin plot in Fig. 2d as well). The re-calibrated dates based on systematic differences show a few low-frequency outliers (Supplementary Fig. 9g), which are likely noises that were ruled out after millions of iterations in BEAST2 simulation process. This is supported by the fact that our BEAST2 estimates with a narrow uncertainty range fall in the range of the re-calibrated dates based on raw sequence divergence (Supplementary Fig. 9g, i). The mean of our date estimates (27.58 thousand years ago) resembles that of the re-calibrated dates after lowfrequency extreme values were removed (26.01 thousand years ago) (Supplementary Fig. 9h, i), which further strengthened the validity of our divergence date estimation.”

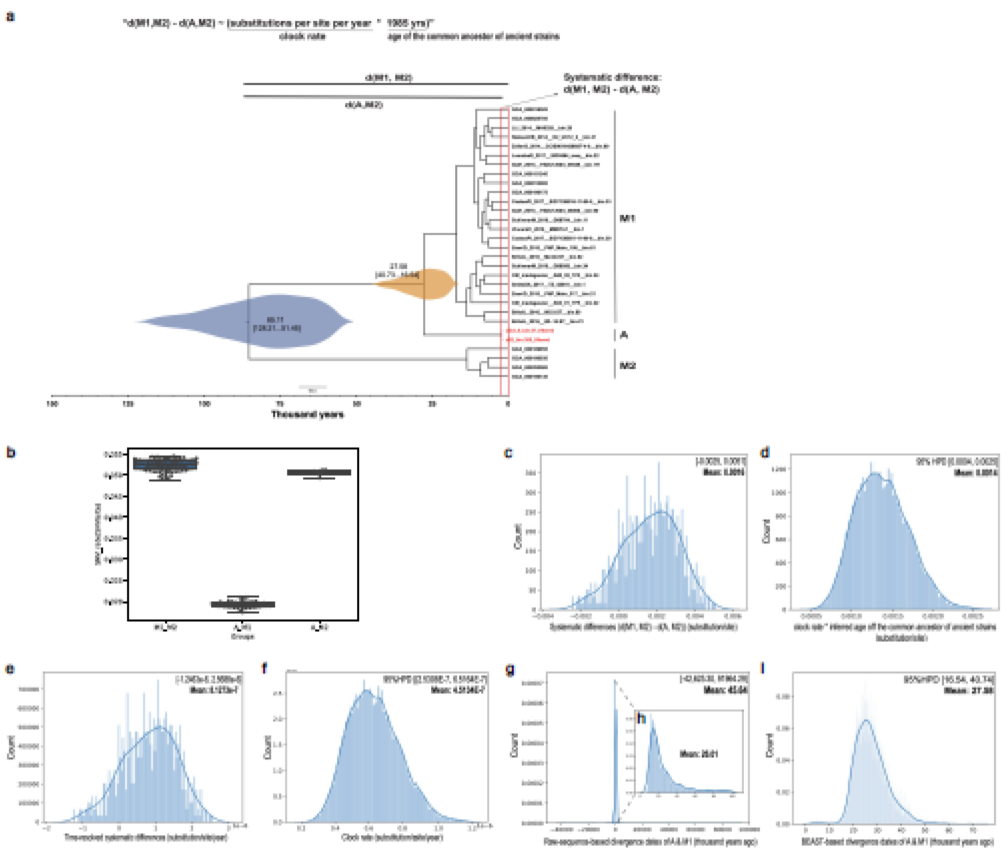

Supplementary Fig. 9. Comparison of *Methanobrevibacter smithii* divergence dates from BEAST2 analysis vs. raw genetic distance calculations (related to Fig. 2).
a, Diagram showing the different *M. smithii* groups and genetic distances calculated.
b, Pairwise sequence divergences between M1 & M2 strains, A & M2 strains, and A & M1 strains.
c-d, Comparison of distribution of systematic differences between M1 & M2 and A & M2 divergences and BEAST2 estimates. **c,** The systematic differences based on pairwise sequence divergences (measured by SNV rate) between M1 & M2 and A & M2 strains. **d,** The products of the clock rates (substitution/site/year) inferred using BEAST2[98] (Supplementary Table 7) and the inferred age of the common ancestor of the ancient strains.
e-f, Comparison of distribution of pairwise time-resolved systematic differences based on raw sequences divergence and the distribution of existing inferred clock rates. **e,** Time-resolved systematic differences calculated by dividing systematic differences (Supplementary Fig. 9c) with the average C14 date of the paleofeces used in molecular clocking analysis. **f,** Clock rates inferred by BEAST2 analysis (Supplementary Table 7).
g, Raw-sequence-based divergence dates between A & M1 strains, re-calibrated using time-resolved systematic differences.
h, Distribution of raw-sequence-based divergence dates when low-frequency outliers are excluded.
i, Distribution of estimated divergence dates between A & M1 strains based on BEAST2 analysis.

Comment: Repeating this analysis for the common ancestor of the ancient strains mentioned above would further support the claim that DNA damage is not severely biasing this signal.
Response: We repeated the same analysis described above using the common ancestor of the ancient strains, as suggested by the reviewer.
As described in pages 97-98: "Moreover, to test whether aDNA damage significantly affected the results, we repeated the same analysis described above using the common ancestor of the ancient strains. We inferred the common ancestor by removing all sites containing SNPs that are different

between the two ancient strains. This is because aDNA damage should occur independently in the two ancient *M. smithii* genomes. We observed and removed 40 such sites (alignment FASTA files and site positions attached). Based on the common ancestor of the two ancient strains, we obtained an average pairwise values of time-resolved systematic differences of 8.2679e-07 (substitution/site/year) and an average of divergences of A & M1 strains of 0.0203 (substitution/site). Thus, the re-calibrated date of divergence between A & M1 strains is 24.66 thousand years ago. The reported divergence dates are not significantly affected by aDNA damage. Instead, the dates shown in Fig. 2d largely reflect the process of mutation accumulation in the course of *M. smithii* genome evolution.
Taken together, the average divergence dates of A & M1 strains re-calibrated using raw sequence divergences, either for the original alignment or the alignment containing only the common ancestor of the ancient strains (26.01 and 24.66 thousand years ago, respectively), is largely consistent with the BEAST2 analysis estimates (27.58 thousand years ago, Fig. 2d). However, compared to calculating raw sequence divergences, the BEAST2 analysis results show a higher confidence in estimation, supported by a narrow range of estimation uncertainty.
The inferred divergences indicate that *M. smithii* radiated in a timeframe compatible with the major human migrations. The origin of the lineage leading to the two ancient *M. smitihii* genomes are set between 40,000 and 16,000 years ago (mean = 27,000 years ago). These estimates predate (although there is a certain overlap toward the earlier 95% posterior estimates) the accepted age of human entry to North America via the Beringia bridge (20,000 - 16,000 years ago)."

Reviewer Reports on the Second Revision:

<u>Referee #1 (Remarks to the Author):</u>

I've mainly focussed on the main text, as I've seen this fine piece of work a couple of times now and found a few minor textual issues and made 2 more scientific comments at the end.

Minor comments:

In addition, we found that microbial 78 composition and functional repertoire of the paleofeces, while possessing many unique features, more closely resemble that of present-day non-industrialized humans compared to present-day industrialized 80 humans.
- add "the" in front of microbial?

107 We analyzed taxonomic composition of the samples with MetaPhlAn223 (Supplementary Table 3), 108 which is a reference-based tool.
- Would perhaps shorten to: We analyzed the taxonomic composition with MetaPhlAn223 (Supplementary Table 3), 108 which is a reference-based tool.

Bayesian inference under a strict clock and the most 197 fitting demographic model (Supplementary Table 7) shows that the ancient M. smithi genomes fall
- add "i" to smiths(i)

In addition, the origin of the lineage leading to the two 204 ancient M. smitihii genomes are set between 40,000 and 16,000 years ago (mean = 27,000 years ago).
- I strongly recommend double checking all instances of M. smithii as M. smitt(i)hii looks like the previous mistake in reverse.

249 The chitin CAZymes are prevalent in MAGs within Oscillospiraceae, Lachnosipraceae, 250 and Clostridiaceae families (Supplementary Information section 14).
- The Lachnospiraceae family would appreciate it to be spelled correctly. Also, should family names not generally be in italics?

Altogether, our data indicate more 251 shared features between the paleofeces and the non-industrial gut microbiome relative to the industrial 252 gut microbiome.
- Sentence is ugly (it feels off). Suggest replacing by: “Altogether, paleofeces appears to have many more features in common with non-industrial gut microbiomes than with industrial ones.”

256 analysis supports that present-day non-industrial human gut microbiomes more closely resemble the 257 paleofeces,
-Remove “the” in front of paleofeces.

Some species, such as Ruminococcus callidus, Butyrivibrio crossotus, and Treponema 259 succinifaciens, are significantly more common in the paleofeces and the non-industrial samples 260 compared to the industrial samples (Fig. 1d, Supplementary Table 3).
- Again, remove “the” 3 times and perhaps replace “compared to” with “than”.

Further, the industrial samples are 261 enriched in mucin-degrading genes (Fig. 3) found in Bacteroides and Prevotella SGBs (Supplementary 262 Information section 14). This is in line with the higher abundance of Bacteroidetes in the industrial 263 samples (Fig. 1), previous findings that members of the Bacteroidetes phylum possess many glycan- 264 degrading genes, and the enrichment of mucin-utilizing enzymes in the industrialized gut.
- Prevotella is not really associated with the industrialized gut, but more with the start/predominance of agriculture (shift from Spirochaetes towards microbiomes more dominated by Prevotella).

269 Our finding supports the hypothesis that mobile genes are important for colonization of the non- 270 industrial gut, perhaps for adaptation to an environment with greater variation, such as seasonal 1 271 variation .
- Or, perhaps you can just find many more elements in a more diverse gut microbiome? —> Seasonal(/other) variation —> Gut microbiome diversity —> More mobile elements? Instead of —> Seasonal(/other) variation —> More mobile elements —> Gut microbiome diversity. Thoughts?

Referee #2 (Remarks to the Author):

I am satisfied with the authors responses to my second round of minor comments. Congratulations on an excellent study and I look forward to its publication.

Referee #4 (Remarks to the Author):

I think the authors have addressed my comments and the comments of the other reviewers well. I cant think of any additional analyses that could improve the manuscript.

Referee #5 (Remarks to the Author):

The authors have satisfactorily addressed all of the points from my last review.

Author Rebuttals to Second Revision:

Response to Referees’ Comments for Manuscript 2020-02-01803B “Reconstruction of ancient microbial genomes from the human gut”

Referees' comments:

<u>Referee #1 (Remarks to the Author):</u>

Comment: I've mainly focussed on the main text, as I've seen this fine piece of work a couple of times now and found a few minor textual issues and made 2 more scientific comments at the end.

Response: We thank the referee for pointing out these issues and we have made changes to the manuscript as detailed below.

Comment: Minor comments:

In addition, we found that microbial 78 composition and functional repertoire of the paleofeces, while possessing many unique features, more closely resemble that of present-day non-industrialized humans compared to present-day industrialized 80 humans.

- add "the" in front of microbial?

Response: We have removed this sentence to save space.

Comment: 107 We analyzed taxonomic composition of the samples with MetaPhlAn223 (Supplementary Table 3), 108 which is a reference-based tool.

- Would perhaps shorten to: We analyzed the taxonomic composition with MetaPhlAn223 (Supplementary Table 3), 108 which is a reference-based tool.

Response: We have now edited this accordingly (lines 110-111).

Comment: Bayesian inference under a strict clock and the most 197 fitting demographic model (Supplementary Table 7) shows that the ancient M. smithi genomes fall

- add "i" to smiths(i)

Response: We have now made this change (lines 195-196).

Comment: In addition, the origin of the lineage leading to the two 204 ancient M. smitihii genomes are set between 40,000 and 16,000 years ago (mean = 27,000 years ago).

- I strongly recommend double checking all instances of M. smithii as M. smitt(i)hii looks like the previous mistake in reverse.

Response: We have now modified this (lines 202-203) and checked all instances of *M. smithii*.

Comment: 249 The chitin CAZymes are prevalent in MAGs within Oscillospiraceae, Lachnosipraceae, 250 and Clostridiaceae families (Supplementary Information section 14).

- The Lachnospiraceae family would appreciate it to be spelled correctly. Also, should family names not generally be in italics?

Response: We have now edited this (lines 239-241) and italicized all bacterial families.

Comment: Altogether, our data indicate more 251 shared features between the paleofeces and the non-industrial gut microbiome relative to the industrial 252 gut microbiome.

- Sentence is ugly (it feels off). Suggest replacing by: "Altogether, paleofeces appears to have many more features in common with non-industrial gut microbiomes than with industrial ones."

Response: We have now modified this sentence to "Altogether, the paleofeces share more features with non-industrial samples than with industrial samples." (lines 241-242).

Comment: 256 analysis supports that present-day non-industrial human gut microbiomes more closely resemble the 257 paleofeces,

-Remove "the" in front of paleofeces.

Response: We are using "the" to refer to the set of eight paleofeces that we analyzed in this study instead of all paleofeces in general (lines 245-247).

Comment: Some species, such as Ruminococcus callidus, Butyrivibrio crossotus, and Treponema 259 succinifaciens, are significantly more common in the paleofeces and the non-industrial samples 260 compared to the industrial samples (Fig. 1d, Supplementary Table 3).

- Again, remove "the" 3 times and perhaps replace "compared to" with "than".

Response: We have removed "the" from in front of non-industrial samples and industrial samples (lines 247-250).

Comment: Further, the industrial samples are 261 enriched in mucin-degrading genes (Fig. 3) found in Bacteroides and Prevotella SGBs (Supplementary 262 Information section 14). This is in line with the higher abundance of Bacteroidetes in the industrial 263 samples (Fig. 1), previous findings that members of the Bacteroidetes phylum possess many glycan- 264 degrading genes, and the enrichment of mucin-utilizing enzymes in the industrialized gut.

- Prevotella is not really associated with the industrialized gut, but more with the start/predominance of agriculture (shift from Spirochaetes towards microbiomes more dominated by Prevotella).

Response: Instead of saying that *Prevotella* is associated with the industrialized gut, we intended to say that among our reconstructed genomes, the mucin-degrading genes are found mostly in our Prevotella SGBs. We have modified the sentence to clarify this (lines 250-251):

"Further, the industrial samples are enriched in mucin-degrading genes (Fig. 4) that are mostly found in our *Bacteroides* and *Prevotella* SGBs (Supplementary Information section 14)."

Comment: 269 Our finding supports the hypothesis that mobile genes are important for colonization of the non- 270 industrial gut, perhaps for adaptation to an environment with greater variation, such as seasonal 1 271 variation .

- Or, perhaps you can just find many more elements in a more diverse gut microbiome? —> Seasonal(/other) variation —> Gut microbiome diversity —> More mobile elements? Instead of —> Seasonal(/other) variation —> More mobile elements —> Gut microbiome diversity. Thoughts?

Response: We compared the proportion of enriched genes that belong to mobile genetic elements (not comparing the number of mobile elements between samples). Therefore, this result was normalized by the total number of elements found in each sample.

Referee #2 (Remarks to the Author):

Comment: I am satisfied with the authors responses to my second round of minor comments. Congratulations on an excellent study and I look forward to its publication.

Response: We are glad that the referee is satisfied with our revision.

Referee #4 (Remarks to the Author):

Comment:I think the authors have addressed my comments and the comments of the other reviewers well. I cant think of any additional analyses that could improve the manuscript.

Response: We are glad that the referee is satisfied with our revision.

Referee #5 (Remarks to the Author):

Comment: The authors have satisfactorily addressed all of the points from my last review.

Response: We are glad that the referee is satisfied with our revision.
